# A Genomic Approach to Examine the Complex Evolution of Laurasiatherian Mammals

**DOI:** 10.1371/journal.pone.0028199

**Published:** 2011-12-02

**Authors:** Björn M. Hallström, Adrian Schneider, Stefan Zoller, Axel Janke

**Affiliations:** 1 Biodiversity and Climate Research Centre (BiK-F) & Senckenberg Gesellschaft für Naturforschung, Frankfurt am Main, Germany; 2 University of Edinburgh, Institute of Evolutionary Biology, Edinburgh, United Kingdom; 3 ETH Zurich, Computational Biochemistry Research Group, Zurich, Switzerland; 4 Goethe University, Institute for Ecology, Evolution and Diversity, Frankfurt am Main, Germany; University of Nottingham, United Kingdom

## Abstract

Recent phylogenomic studies have failed to conclusively resolve certain branches of the placental mammalian tree, despite the evolutionary analysis of genomic data from 32 species. Previous analyses of single genes and retroposon insertion data yielded support for different phylogenetic scenarios for the most basal divergences. The results indicated that some mammalian divergences were best interpreted not as a single bifurcating tree, but as an evolutionary network. In these studies the relationships among some orders of the super-clade Laurasiatheria were poorly supported, albeit not studied in detail. Therefore, 4775 protein-coding genes (6,196,263 nucleotides) were collected and aligned in order to analyze the evolution of this clade. Additionally, over 200,000 introns were screened *in silico*, resulting in 32 phylogenetically informative long interspersed nuclear elements (LINE) insertion events.

The present study shows that the genome evolution of Laurasiatheria may best be understood as an evolutionary network. Thus, contrary to the common expectation to resolve major evolutionary events as a bifurcating tree, genome analyses unveil complex speciation processes even in deep mammalian divergences. We exemplify this on a subset of 1159 suitable genes that have individual histories, most likely due to incomplete lineage sorting or introgression, processes that can make the genealogy of mammalian genomes complex.

These unexpected results have major implications for the understanding of evolution in general, because the evolution of even some higher level taxa such as mammalian orders may sometimes not be interpreted as a simple bifurcating pattern.

## Introduction

While the placental mammalian tree is becoming increasingly better resolved, it has proven difficult to fully resolve several branches of it as a bifurcating tree, despite the availability and analyses of whole genome data [1], [2]. While the sheer amount of genomic data should be sufficient to resolve very short branches within the placental mammalian tree [2], the support for some branches is often ambigious. Interestingly, these problematic branches are characterized by rapid divergences within 1–3 million years (Myr) [2]. This makes it possible that speciation related processes, such as incomplete lineage sorting or introgression, lead to gene trees that differ from the species tree [3], [4]. The complex pattern of retroposon insertion data for the earliest placental mammalian divergences [5] corroborate this idea, suggesting that a network-like evolution instead of a bifurcating tree best depict and interpret the evolutionary process [2]. Other such problematic relationships among placental mammals have been identified by phylogenomic [1], [2] and retroposon insertion data [6]. A case in point is the evolution of the mammalian clade Laurasiatheria, which comprises several orders of placental mammals.

Laurasiatheria include the classical orders Perissodactyla, Carnivora, Pholidota, Artiodactyla, Cetacea, Chiroptera, and Lipotyphla [7], [8]. Initially, morphological and early molecular studies spread these orders to different parts of the mammalian tree or left their position unresolved [9]. More detailed molecular phylogenetic studies grouped these diverse orders into one clade, Laurasiatheria. Early mitogenomic studies suggested a close relationship between carnivores and perissodactyls and this group in turn joined Cetartiodactyla [10], [11]. Later mitogenomic studies added Chiroptera [12] and parts of a then paraphyletic Lipotyphla to the Laurasiatheria clade [13], whereas analysis of nuclear genes placed all of Lipotyphla within Laurasiatheria [14]. Finally, the Pholidota (pangolin) were joined with the carnivores by nuclear and mitogenomic studies [11], [14].

Currently molecular phylogenetic studies generally agree on a (Chiroptera,(Cetartiodactyla, (Perissodactyla, (Pholidota, Carnivora))) branching order [11], [14]. With the exception of the Pholidota, which lack large-scale genomic sequence data, recent phylogenomic analyses generally support this topology. However, the relationships remained only poorly supported despite the use of some 3 million nucleotides of sequence data from 3400 protein coding genes [2].

So far only one study using rare genomic events such as data from retroposon insertions has been made to study the relationships within Laurasiatheria. In contrast to sequence-based studies, analyses of three retroposon insertions support the grouping of Chiroptera with the Perissodactyla/Carnivora and a new name for this unexpected clade – Pegasoferae – has been suggested [6]. Yet, this study found one retroposon insertion event that contradicted the Chiroptera plus Perissodactyla/Carnivora grouping. This one retroposon insertion supports the traditional sequence-analyses based placement of Chiroptera.

A recent phylogenomic study of mammalian relationships involved all tetrapod species from which whole genome data were available. While it is advantageous to increase taxon sampling, this approach leads to the exclusion of large amounts of sequence data when stringent data collection and alignment strategies are employed [1], [2]. In addition, the inclusion of distantly related species in the analyses even make it possible that orthologs are misidentified, and thus excluded, as paralogs by overly stringent data retrieval algorithms such as recursive BLAST [1].

In order to specifically analyse laurasiatherian relationships with a dataset maximized for the amount of phylogenetically informative data, only human and mouse are used as outgroups to root the tree in this study. These species have among the best genomic sequence coverage and annotation. Furthermore, there is an unequivocal consensus that these two species are joined in the clade Euarchontoglires which is the sister group to Laurasiatheria within Boreotheria. Thus, human and mouse are the ideal outgroups for this study. By also utilizing the recently released genome of the giant panda (*Ailuropoda melanoleuca*) [15], this approach allows the collection of a larger number of genes from more species than in previous phylogenomic studies. Therefore, analyses based on concatenated data and single genes allow for a more detailed study of laurasiatherian relationships. In addition, the quality and quantity of the genome data have been steadily improving. This makes *in silico* searches for phylogenetic informative retroposon insertion data feasible for evaluating hypotheses that were based on sequence data analysis. Long interspersed nuclear elements 1 (LINE 1) retroposon sequences were used for these searches, because these elements were active during this time of placental mammalian evolution and have successfully been used in other phylogenetic studies [16]–[18]. These are currently the only known retroposons that are common to different orders, while short interspersed nuclear elements (SINEs) are order-specific [19].

## Methods

### Sequence analysis

The complementary DNA (cDNA) databases for all species included in the study, except the panda, were downloaded from Ensembl (http://www.ensembl.org, release 57). The whole genome sequence of the panda was downloaded from the Giant Panda Database (http://panda.genomics.org.cn/) and the cDNA sequences were extracted using the gene annotation based on homology to dog genes. Table 1 lists the included species. For some comparisons the genome data from the opossum were included in the analyses.

**Table 1:** The names, order and sequence coverage of the species included in this study.

Common name	Binomial name	Order	Coverage
Dog	*Canis familiaris*	Carnivora	100%
Cat	*Felis catus*	Carnivora	67.7%
Giant Panda	*Alluropoda melanoleuca*	Carnivora	98.1%
Horse	*Equus caballus*	Perissodactyla	98.0%
Cow	*Bos taurus*	Cetartiodactyla	92.9%
Bottlenose Dolphin	*Tursiops truncatus*	Cetartiodactyla	91.8%
Pig	*Sus domestica*	Cetartiodactyla	77.7%
Alpaca	*Vicugna pacos*	Cetartiodactyla	66.7%
Large Flying Fox	*Pteropus vampyrus*	Chiroptera	91.7%
Little Brown Bat	*Myotis lucifugus*	Chiroptera	77.1%
European Hedgehog	*Erinaceus europaeus*	Erinaceomorpha	74.8%
Common Shrew	*Sorex araneus*	Soricomorpha	68.1%
Human	*Homo sapiens*	Primates	100%
House Mouse	*Mus musculus*	Rodentia	90.8%
Gray Short-tailed Opossum	*Monodelphis domestica*	Didelphimorphia	84.4%

Note – Coverage give the percent sequence coverage in the 6,196,263 nt alignment.

Data collection and alignment was, with a few exceptions, performed as described previously [2] and is thus only briefly detailed here. Orthologs were identified with the recursive BLAST method [1]. Sequences were translated to amino acids and aligned using MUSCLE [20]. The resulting alignments were then back-translated to nucleotides. Any alignment showing an overall nucleotide difference larger than 30% between any two species was discarded. As an additional filtering step, uninformative quickly evolving sites were eliminated by the program Noisy, version 1.5.9 [21].

Phylogenetic analysis using maximum likelihood was performed using the programs Treefinder (TF) [22] and RAxML 7.0.4 [23], applying the GTR model [24] to nucleotide data and WAG2000 [25] to amino acid data. In both cases, rate heterogeneity was applied using 4 gamma rate categories, 4G+I. Both heuristic searches and exhaustive tree comparisons, under the assumption of monophyletic orders were performed. Divergence times were estimated from overall best amino acid (AA) ML tree using 6 calibration points [26] (Table S1) and the nonparametric rate smoothing method on a logarithmic scale (NPRS-LOG) as implemented in TF [22].

Codon-based tree reconstruction was performed using the Markov Chain Monte Carlo (MCMC) method implemented in BEAST [27], using its BEAGLE library for computing on graphics processing units (GPUs). This decreases computation times by a factor of up to 90 [28]. The analyses were performed using a semi-parametric codon model based on principal component analysis of mammalian sequence data [29]. For each alignment and topology, the model-parameters as well as the branch lengths were optimized with a chain length of 700,000 sampled every 500 tree. Instead of maximizing the likelihood, BEAST allows an estimation of the marginal log-likelihood (mLogL) by integrating over the whole parameter space [30], [31]. Tree and model comparisons can be performed using the Bayes factor [32], which can be approximated as the difference of the mLogLs. For 229 trees, BEAST failed to successfully optimize the parameters. These trees were excluded from further analysis.

In addition to the analyses of concatenated data, all gene alignments with sequence data from all species were analyzed separately. The problem was reduced to resolving the relationships of four orders, leaving 15 possible topologies that were individually evaluated by ML analyses for each of the 1159 gene alignments. The same models as outlines above were used with parameters estimated from individual alignments. Information from likelihood maximizations on the 15×1159 gene trees were analyzed by counting how often each topology was among the most likely trees and how often a topology was rejected by another topology with a significantly higher likelihood. A significantly higher likelihood is defined as one that is larger than two log-likelihood units from the original. When different topologies had the same likelihood for single-gene alignments, they were counted individually for each tree. In addition, all likelihood values for a given topology and data set were added up in order to compare the total likelihoods of the different topologies. This approach corresponds to the “separate” analysis according to the definition in Pupko et al., [33]. Since the mLogLs of the codon-based analysis are expected values and not maxima, so typically no two topologies end up with exactly the same mLogL. Thus, a tolerance of 0.5 LogL units was used, and two values that lay within two mLogLs of each other were considered as being equal. A topology was rejected if its mLogL was 10 units lower than the highest. The ML trees from the single-gene analysis were also used to construct a consensus network using the SplitsTree4 program [34], which is used to illustrate the conflicts of the phylogenetic signal.

For the five most likely tree topologies, the influence of several properties of the sequences on the outcome of the codon analysis was tested. The evaluated factors were alignment length, longest distance among the 15 sequences, sum of all pair wise distances, deviation of the codon usage frequencies from the average over all alignments and deviation of the nucleotide usage frequencies from the average. For each factor, the alignments were divided into two equal-sized groups; those with the largest values and those with the smallest values. It was then counted how often each topology was the only one with highest mLogL. Chi-square tests were performed to quantify the significance of the difference between the two “best” distributions of topologies.

Finally, a multilocus Bayesian analysis using the program BEST [35] was performed. This method attempts to construct a species tree using a multiple estimated gene trees. This is done by utilizing a Bayesian hierarchical model to combine traditional phylogenetics with coalescent theory. 763 genes (1,313,880 nucleotide characters) were selected for maximum alignment coverage and length. This data set was analyzed in BEST, with all parameters unlinked, runnning for 15,000,000 generations, with two simultaneous runs each with one “heated” and one “cold chain. The first 1.500.000 generations (10%) were discarded as burnin.

### Retroposon analysys

For the retroposon insertion analysis intron sequences longer than 300 bp and shorter than 3000 bp were collected from the Ensembl database (version 49) for the cow, dog, horse and microbat genomes, respectively. Between 40,000 and 95,000 introns were identified in each of the species above. Retroposed elements in these introns were identified using the program RepeatMasker version 3.2.8 (http://www.repeatmasker.org/). From all identified repeated elements only LINE1 elements were considered for the search and phylogenetic analysis. In total 47,535 LINE1 elements were identified, of which 22,873 were found in the horse genome, 13,359 in the dog genome, 6,557 in the cow genome and 4,756 in the bat genome. Using these intron sequences the orthologous region in the other three species were identified. The full sequences of orthologous genes were extracted, based on Ensembl orthology data. The relevant intron sequences were located by making local pair wise alignments with 80 bp of exon sequence located upstream and downstream of the intron. In cases where the intron could be located in all four species a four-way multiple sequence alignment was created using MAFFT [36]. This resulted in 19,725 alignments that were guided by 7576 retropson insertions that were initially identified in the horse, 7248 in the dog , 2793 in the cow, and 2108 in the bat, respectively. All four-way alignments were screened for retroposons that were present in either two or three of the species, and absent in the others. These retroposon inserts were considered potential markers for the phylogenetic relationships between the four orders. Finally intron sequences from the remaining laurasiatherian species and, when possible, outgroup sequences were added to the alignments.

Additionally, several hundred alignments in which the insertion was present in either only one or all four species were randomly selected and manually screened for potentially informative retroposon markers for other parts of the laurasiatherian tree. The alignments of the, in total 25, informative L1 retroposon insertions that were used for the tree and network are shown in Figure S2. A consensus tree, was constructed with SplitsTree4 from all partial trees corresponding to the L1 retroposon data for the conflicting hypotheses among the four laurasiatherian orders. The branch lengths are determined by the number of retroposon insertions supporting each topology.

## Results

### Sequence analysis

The final alignment consisted of 6,196,263 nucleotide characters (translating to 2,065,421 amino acid characters) from 4775 genes, represented by 12 ingroup species and two outgroup species; human and mouse. Table 1 provides a list of all included species and their sequence coverage in the alignment. After eliminating potentially homoplastic sites, the alignment length was reduced to 4,314,195 characters for the nucleotide data and 1,476,398 characters for the amino acid data. The average sequences coverage of the alignment was 85.3%.

**Figure 1:**
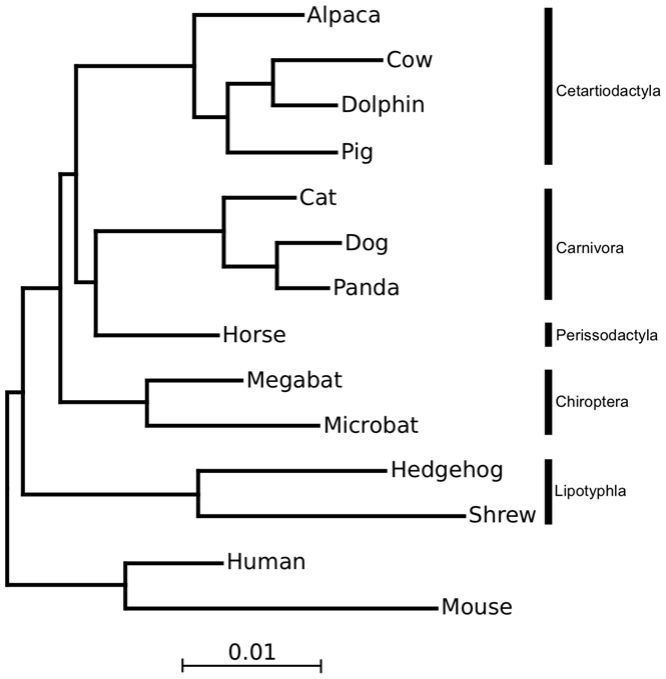
Best ML tree based on concatenated amino acid data.

In heuristic analyses and RAxML parametric bootstrap analysis the relationships within the orders were unanimous, but some inter-ordinal relationships received only limited support. Figure 1 shows the best-supported tree and branch lengths based on maximum likelihood (ML) analysis of concatenated amino acid (AA) data. This topology was also the best or among the best supported in other analyses. For further evaluating the topology and the support assigned to it, exhaustive analyses were performed on the relationships among the five laurasiatherian orders, testing all 105 possible rooted topologies. Among these 105 trees any topology where Lipotyphla was not the first diverging order received significantly lower support in all analyses. Thus, in the following only the remaining 15 proposed trees among Chiroptera, Cetartiodactyla, Perissodactyla, and Carnivora were analyzed in more detail.

**Figure 2:**
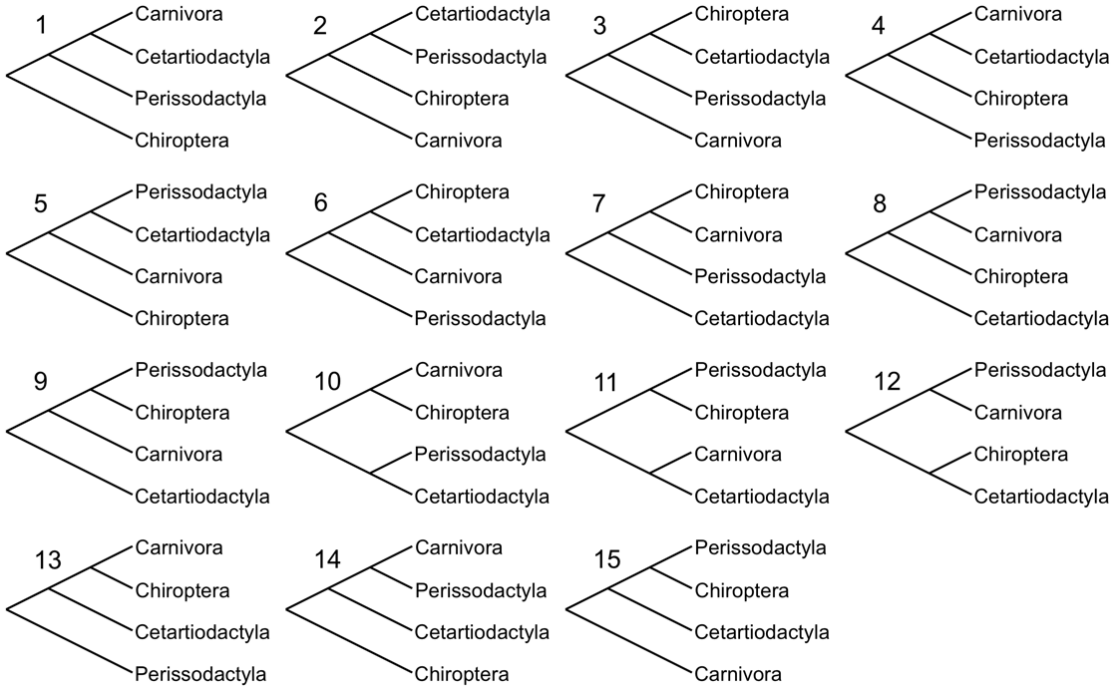
Overview of the rooted topologies among four orders that have been individually tested.

The 15 topologies are shown and numbered in Figure 2. The Shimodaira-Hasegawa probabilities (pSH) [37] for these 15 topologies are shown in Table 2. Tree 14 is favored by most analyses and not rejected by any analysis. This tree corresponds to that shown in Figure 1. While the AA and NT12 (nucleotides, first and second codon position) datasets do not provide conclusive support for a single tree, tree 14 is significantly supported by NT123 (nucleotides, all codon positions). Removing the most distant outgroup, opossum, from the analysis generally increases the support for topology 14 relative to the others, illustrating the importance of using a close outgroup for phylogenetic analyses.

**Table 2:** pSH values for 15 topologies regarding the relationship among Chiroptera, Perissodactyla, Carnivora, and Cetartiodactyla.

	TF pSH(AA)		TF pSH(NT12)		TF pSH(NT123)		RAxML (w/o opossom)		
Tree	*w opossum*	*w/o opossum*	*w opossum*	*w/o opossum*	*w opossum*	*w/o opossum*	AA	NT12	NT123
1	0.00025	0	0	0	0	0	*	*	*
2	**0.1125**	0	0	0	0	0	*	*	*
3	0	0	0	0	0	0	*	*	*
4	0	0	0	0	0	0	*	*	*
5	**1.0**	**1.0**	**1.0**	**0.87255**	0	0.0019	**N/R**	**BEST**	*
6	0	0	0	0	0	0	*	*	*
7	**0.2779**	**0.39115**	0	0	0	0	*	*	*
8	**0.2663**	**0.3562**	0.00095	0.0001	0	0	*	*	*
9	0	0	0	0	0	0	*	*	*
10	**0.135**	**0.26545**	**0.1078**	0.0136	0	0	**BEST**	*	*
11	0	0	0	0	0	0	*	*	*
12	**0.066**	0.0014	0	0	**0.0911**	0	*	*	*
13	0.02895	0	0	0	0	0	*	*	*
14	**0.48995**	**0.58705**	**0.8414**	**1.0**	**1.0**	**1.0**	**N/R**	**BEST**	**BEST**
15	0	0	0	0	0		*	*	*

Bold typeface indicate that the topology is not rejected. RAxML does not provide probability values and instead shows only if the topology is the most likely (BEST), not rejected (N/R) or rejected at the 0.05 significance level (*).

Note – “w opossum” and “w/o opossum” denotes whether or not opossum was included as an outgroup. 0 denotes a probability below 0.0001.

The estimation of divergence times was complicated by the lack of distinctive outgroups with a well-defined maximum age. Using the soft lower bounds (Table S1) yielded unexpected ancient divergence times among all groups. By constraining the deepest divergence to 92 Ma [1] divergence times that are in agreement with previous phylogenomic studies were estimated. Thus the radiation among the different order occurred 87–60 Ma. While the absolute dates may be debatable, the relative divergence times of short branches that are problematic to resolve were in the order of 2 Myr (Figure S1).

Topology 5 receives the second best support, joining Perissodactyla and Cetartiodactyla [38], to the exclusion of Carnivora. This hypothesis is the best supported in AA analyses, but clearly rejected by NT123 data. There is no majority consensus among the analyses or data sets. Interestingly, topology 8, termed Pegasoferae [6] was favored in an earlier retroposon insertion analysis, but receives low support by AA data using TF, and is significantly rejected in all other sequence analyses. In general, the lack of clear support for a single topology mirrors the results and conclusions on the most basal placental mammalian divergences [2]. Therefore, network analysis methods were employed to investigate the conflict in the data.

**Figure 3:**
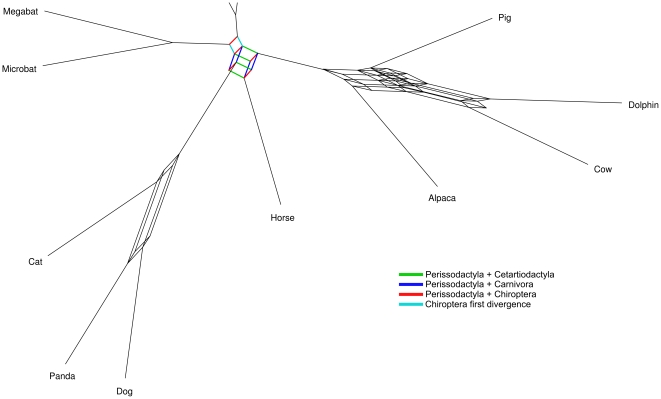
Consensus network of 1159 trees based on alignments with sequence for all species, using a threshold value of 8%.

Figure 3 shows a consensus network based on 1159 trees calculated from ML analyses of the alignments of single genes for which sequence data for all 14 species were available. The relationships between the four orders Carnivora, Chiroptera, Cetartiodactyla, and Perissodactyla are largely unresolved in this analysis, as represented by the cube-like structure in this part of the network. For the lack of an acceptable name this clade will be abbreviated by the initial letters of the orders as “CCCP-clade”. The cube-like structure illustrates the roughly equal support for placing an order in either topology along parallel branches. In other parts of the network, i.e. within Cetartiodactyla and Carnivora, the relationships are depicted by elongated structures, indicating that one of the topologies is preferred over the other by this analysis.

The results of the individual gene trees were also analyzed, with the summary shown in Table 3. The table shows, for each data set (AA, NT12, NT123 and codon) and each of the 15 topologies, the number of times the topology was among the ones with the maximal likelihood value, how often it was rejected, and the sum of the log-likelihoods. As in the analysis of the concatenated genes, no consensus is found among methods and data sets, but a few trends can be observed. Tree 5 always has the highest log-likelihood sum and is rejected the fewest numbers of times in the NT12 and NT123 data sets. It is also the most frequent ML tree in the NT123 and codon data sets. For most of these combinations of data and methods, tree 14 follows in second position. For certain analyses on the AA and NT12 data set, trees 2, 10 and 12 have the highest support, but all three of them are firmly rejected by other analyses. It is also noteworthy, that the NT analyses, in particular NT123, allow for a much stronger separations among the topologies. In the AA data set, the number of times a tree is among the best ranges from 157 to 175, whereas for the NT123 data set, it ranges from 86 to 181, a span that is more than five times as large. Also, the highest log-likelihood difference for AA data is 233.0 compared to 977.5 for the NT123 data. This may be an indication that the rate of amino acid substitution is often too low to distinguish between the very short branches separating the orders, while the numerous synonymous third codon positions may still allow to better resolve some branches, despite their advanced state of randomization at 80 Ma.

**Table 3:** Analysis of 1159 gene trees.

	AA			NT12			NT123		
Tree	Best	Rejected	ΔlogL	Best	Rejected	ΔlogL	Best	Reject	ΔmLogL
1	154	423	−95.3	208	450	−148.6	103	106	−369.1
2	**175**	405	−91.9	222	*426*	*−12.3*	95	*88*	*−149.2*
3	159	418	−166.7	168	481	−350.8	76	117	−572.6
4	158	407	−158.0	191	478	−429.9	90	122	−591.4
5	163	402	**0.0**	*239*	**418**	**0.0**	**120**	*88*	**0**
6	164	414	−195.3	181	481	−452.4	97	118	−663.1
7	147	422	−233.0	186	476	−361.8	86	129	−836.9
8	167	*397*	−148.7	204	439	−114.1	92	105	−521.4
9	161	404	−142.7	205	459	−218.0	92	110	−617.8
10	168	413	−129.7	**240**	435	−135.2	99	92	−176.2
11	149	407	−132.5	208	449	−227.3	92	106	−356.1
12	*173*	**387**	−35.7	213	439	−143.2	101	108	−349.8
13	161	412	−206.2	202	466	−371.2	96	116	−671.6
14	157	409	*−22.6*	220	430	−32.4	*104*	**87**	−199.3
15	160	416	−154.9	194	472	−256.4	94	100	−399.5

For each of the 3 data types (AA, NT12 and NT123) and tree topology, the number of times the topology was among the best (ML), the number of rejections and the difference of the sum of the log-likelihoods to the best one are reported. Bold numbers indicate the best values in a column, while numbers in italics indicate the respective second best values.

The analysis of the influence from sequence and alignment properties on the resulting best topology is shown in Table 4. To exclude irrelevant changes among less likely trees, only the five most likely trees were compared. Some factors have an influence on the results. Tree 10, for example, gets almost double the support from long alignments than from short ones, whereas trees 12 and 14 find more support when longer distances separate the sequences. Overall, the sequence distance has the largest effect on the distribution of supported trees. Alignments with long distances favor tree 14 and disfavor trees 2 and 5. Although this shows that sequence specific aspects can influence the topology, none of the chi-square tests indicate a significant difference between them.

**Table 4:** Analysis of the influence of five aspects of the alignments on the frequency of the five most likely trees.

Measure		Trees					χ^2^
		2	5	10	12	14	
Alignment length	short	22	25	**14**	20	25	2.86
	long	23	26	**22**	14	24	
Longest distance	low	26	27	17	16	**20**	3.11
	high	19	24	19	18	**29**	
Sum of distances	low	19	26	18	15	**20**	1.57
	high	26	25	18	19	**29**	
Codon usage bias	average	25	25	18	21	**29**	2.08
	extreme	20	26	18	13	**20**	
Nucleotide usage bias	average	24	27	17	**20**	24	1.19
	extreme	21	24	19	**14**	25	

The numbers indicate how often each topology was the only highest supported topology. With four degrees of freedom, none of the χ^2^ values are significant. The bold number pairs indicate the largest change for each measure.

The Bayesian species tree reconstruction, using the program BEST, from 764 alignments selected for length and sequence coverage did not yield a clearly supported bifurcating tree. Posterior probabilities above 0.05 were calculated for three trees: the probabilities were 0.43 for tree 15, 0.26 for tree 9 and 0.19 for tree 11.

### Retroposon analysis

**Figure 4:**
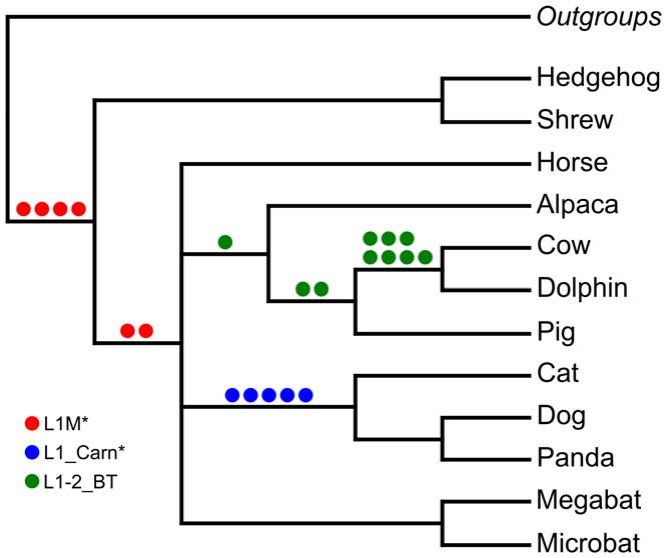
Non-controversial retroposon markers shown on a tree.

The alignments of the informative retroposon inserts are shown in Figure S2. For the monophyly of the uncontroversial laurasiatherian, Carnivora, and the cow-dolphin clade (Cetruminantia) [39] four to seven retroposon insertions were identified. In addition, non-significant support, i.e. less than three retroposon insertions [40] were found for the monophyly of the uncontroversial Cetartiodactyla, the CCCP-clade and the pig-cow-dolphin-clade. The retroposon insertions that support uncontroversial groupings are summarized in Table 5A and shown in Figure 4. For these clades no contradictory signal from retroposon insertion marker were identified.

**Table 5:** Number (#) of retroposon markers supporting relationships among Laurasiatheria.

a) Uncontroversial relationships	#
Carnivora monophyly	5
Laurasiatheria monophyly	4
Lipotyphla first divergence in Laurasiatheria	2
Dolphin-Cow	7
Dolphin-Cow-Pig	2
Cetartiodactyla monophyly	1

b) Incompatible relationships	#
((Carnivora+Chiroptera+Perissodactyla), Cetartiodactyla)	3
((Carnivora+Chiroptera+Cetartiodactyla), Perissodactyla)	3
((Perissodactyla+Chiroptera+Cetartiodactyla), Carnivora)	2
((Carnivora+Perissodactyla), Chiroptera, Cetartiodactyla)	2
((Carnivora+Chiroptera), Perissodactyla, Cetartiodactyla)	1

**Figure 5:**
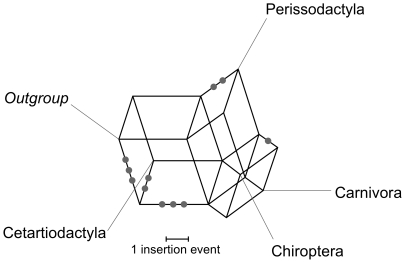
Network of relationships supported by retroposon insertion data.

Apart from these non-controversial markers, a number of mutually incompatible retroposon insertions were found that support different inter-ordinal relationships of the four orders Carnivora, Chiroptera, Cetartiodactyla, and Perissodactyla. Table 5B summarizes the support from retroposon insertion data for different topologies and Figure 5 depicts the network that can be reconstructed from it. An equal number of three markers support the hypotheses that Perissodactyla or Cetartiodactyla represent the first divergence among the four orders, while two marker support Carnivora as the first divergence. In addition two markers support a grouping of Carnivora and Perissodactyla and one marker group Carnivora with Chiroptera.

## Discussion

Compared to phylogenetic analyses that were done in the 1990s and were based on single genes or a combination of a few sequences, the advance in genome sequencing now make it possible to analyze thousands of sequences, which promises a huge increase in the accuracy of the reconstructed tree. In this study 4775 protein-coding sequences were used to reconstruct the evolutionary history of a major clade of placental mammals, the Laurasiatherian.

The best-supported tree in the phylogenomic analyses on concatenated data among laurasiatherian orders (Figure 1) conforms to that of previous mitogenomic and nuclear gene analyses [11], [14]. All analyses agree that Lipotyphla represent the first divergence within this clade. Also, most sequence analyses can significantly reject some hypotheses, such as the recently proposed Pegasoferae hypothesis [6]. However, the support for bifurcating inter-ordinal relationships is surprisingly limited. Two incompatible hypotheses of laurasiatherian relationships cannot be ruled out and were even estimated to be the best ML tree in some analyses. This indicates conflicting phylogenetic signals from the sequence data.

It has been suggested that separate analysis in which each gene is evaluated individually is preferable to the analyses of concatenated sequences, because this approach improves the estimation of likelihood parameters [33], [41]. Yet, separate analyses of single genes lead to the same phylogenetic conclusions as the analysis of concatenated data. The single gene analyses do not favor a single tree but find support for alternative hypothesis, as illustrated in the network of Figure 3. This point is nicely depicted in the network of topologies reconstructed from single genes. Short sequences, however, by their nature often do not contain enough information to significantly distinguish between different topologies. A solution to this problem is the combination of likelihood values from single gene analyses [33]. This approach, like the concatenated analyses, favors topologies 5 and 14 in all analyses.

In addition, the influence of key characteristics of the individual sequences (such as sequence length, rate of evolution and composition) on the reconstructed trees was investigated, because different subsets of the data may have different reconstruction biases, such as long branch attraction [42]. Such biases would cause substantial numbers of sequences supporting conflicting topologies. Yet, none of the five tested characteristics had a significant effect on the distribution of the favored topologies. Although there are certainly additional, but untested properties of the sequences or alignments, the outcome of this study supports the idea that the data contain truly conflicting phylogenetic signals rather than subsets of genes that are affected by different reconstruction biases. The conflicting evolutionary signals from single genes cannot be reconciled into a bifurcating tree, even when using reconstruction methods that take coalescence models into account. This method is supposed to allow reconstruction of a species tree despite the presence of incomplete lineage sorting, but does not account for any sort of lateral gene transfer or introgression through hybridization.

With current methods it is difficult to distinguish between incomplete lineage sorting and introgression. However, both processes have a profound effect on the definition of a species at the genomic level, because it causes alleles to be shared between species, which then contradict each other in delineating species or estimate their divergences. Even over long time periods these shared alleles are influencing phylogenetic reconstruction, despite many new mutations, which are unique to each order. Thus, the genomes of today's orders, which started out 70 Ma as different populations and then species, retain information of the past speciation events.

Not only incomplete lineage sorting, but also hybridization occurs more frequently in animals than previously assumed. While hybridization has generally been considered to hinder evolutionary diversification [43], hybridization from distant populations or other species can introduce novel mutation, increasing the possibility for adaptation [44]. Evidence for hybridization that aid adaptation has been described in insects [45] and fishes [46], and is not unexpected to occur in birds and mammals, given the frequency of hybridization in these [47]. A number of hybridization events in mammals have been described, indicating that it may not be a rare process [48] and consequently hybridization in animals gains an increasing interest [49].

Finally, the application of close outgroups has not only increased the amount of data but also yielded more consistent results compared to when a more distant outgroup, the opossum, is used. This agrees with previous observations that suggest using a closer outgroup often increases the level of support for the correct topology [50].

The support for different topologies, as provided by individual loci becomes obvious in the retroposon analysis. This study focused on LINE 1 elements, which were active during this time of placental mammalian evolution [16]–[18]. The conflicts in the resolution of the relationships of the CCCP-clade by retroposon data mirror the sequence-based analyses of these relationships. In particular, divergences within the CCCP-clade for which the inter-ordinal relationships were not clearly resolved by sequence data analyses, were studied in detail by retroposon insertions. A number of retroposon insertions for a possible Pegasoferae relationship ((Perissodactyla, Carnivora), Chiroptera) have been found, but unlike in the study of Nishihara et al. [6], the current study identified numerous conflicting retroposon insertion, supporting alternative relationships (Figure 5). In comparison, well-resolved relationships within Laurasiatheria are unambiguously resolved by retroposon data (Figure 4). For these unambiguous groups no contradictory signals were identified in this survey. The congruent results from retroposon data and sequence based analyses, support the view that the lack of resolution from sequence data is not caused by systematic errors.

Retroposon insertion data are, with very few exceptions, regarded as being homoplasy free [51]–[53]. The rarity and very mechanism of retroposon insertion support the idea that retroposon insertion reversals or parallel events are non-existing or extremely uncommon [54]. However, these and previous findings [5] show that retroposon insertion data can still produce contradictory phylogenetic signals stemming from genomic events that are connected with speciation, such as incomplete lineage sorting or hybridization. In fact, apparently contradictory sequence data and retroposon data in this study, along with that provided by an investigation into the early placental mammalian evolution may be best interpreted as a result of such processes [2]. However this leads to a problem when regarding the statistics of branch support from retroposon insertions.

The premises for the hypothesis that three retroposon insertions are sufficient to significantly support a branch [40] was that these data are homoplasy free and do not produce conflicting data. However, as outlined above evolutionary processes do produce conflicting phylogenetic signals from retroposons, if one interprets the data in a strictly bifurcating tree [2], [5], [6]. Thus, the simple statistics that suggests that three retroposon insertions in one branch yield significant support needs to be revised to include the possibility of conflicting signal.

Sequence data and retroposon insertion data can lead to apparently inconsistent hypotheses, when viewed as a bifurcating tree. A sequence based tree analyses of concatenated sequences represents only an average of the phylogenetic signal. Most phylogenetic information can get lost or distorted. However, the complex pattern from sequence and retroposon-based analyses can better be depicted as networks [34] and easily explain apparent inconsistencies and allow illustrating and exploring conflicting data. This way apparent inconsistencies are naturally resolved by making sense out of the complex evolutionary patterns. By placing all events on separate branches, alternative evolutionary pathways and gene-trees are revealed (Figure 5).

The problem of phylogenentic inconsistencies arises only when one ignores the possibility of complex evolutionary history and tries to force them into a traditional, two-dimensional bifurcating tree. Complex evolutionary patterns or conflict of rare genomic events have now been described for Laurasiatheria, hominoid divergences [55], basal placental mammalian divergences [2], [5], and other mammalian lineages [55]–[57].

In all cases of complex speciations the divergence times among the groups are relatively short [2]. This is also the case for Laurasiatheria in which the estimated times for some groups are within about 2 Myr of each other. This is, as discussed earlier for other divergences [2], within the order of speciation times of divergence and species durations [58], [59], which can lead to the complex pattern of gene trees. Speciation related processes have obviously influenced the evolution of placental mammals to a much larger extent than expected and in many cases do not allow the reconstruction of bifurcating divergences. Stochastic errors of small datasets aside, conflicting trees that were in previous studies based on small data sets may actually reflect alternative evolutionary scenarios of single genes in the genome, resulting in different gene trees [60]. Although prokaryote evolution represent an extreme case of network-like evolution [61], [62], the evolution and speciation of vertebrates may be more complex than previously thought.

With the advent of more genome data becoming available, along with the ability to explore deep divergences in greater detail, it is becoming evident that evolutionary processes are best interpreted as networks. Networks naturally highlight the conflict and difficulties of previous phylogenetic studies to find a congruent bifurcating tree within this group. The hope of phylogeneticists that whole genome data would one day yield a single, stable and bifurcating evolutionary tree [18], [63], [64], is not fulfilled for some parts of the placental mammalian tree. However, it seems that a more valuable lesson can be learned from genome analyses. That is, some divergences are not characterized by bifurcations but rather that the evolution of some placental mammals represent a complex pattern of genealogies of different parts of the genome. Speciation processes that can be revealed from genome data even for deep divergences, define this pattern. The evolution of Carnivora, Perissodactyla, Chiroptera, and Cetartiodactyla (Laurasiatheria) represent such a case.

## Supporting Information

Figure S1 **Chronogram showing the estimated times of divergence.**
(PDF)

Figure S2 **Alignments of all informative retroposon insertions found in this study.**
(PDF)

Table S1 **Upper and lower bounds of the calibration points used in the divergence time estimation.**
(DOC)
