# Supplementary figures and images for: A Genomic Approach to Examine the Complex Evolution of Laurasiatherian Mammals
Source: PLoS One. 2011 Dec 2;6(12):e28199. doi: 10.1371/journal.pone.0028199 (PMC3229520; doi:10.1371/journal.pone.0028199)

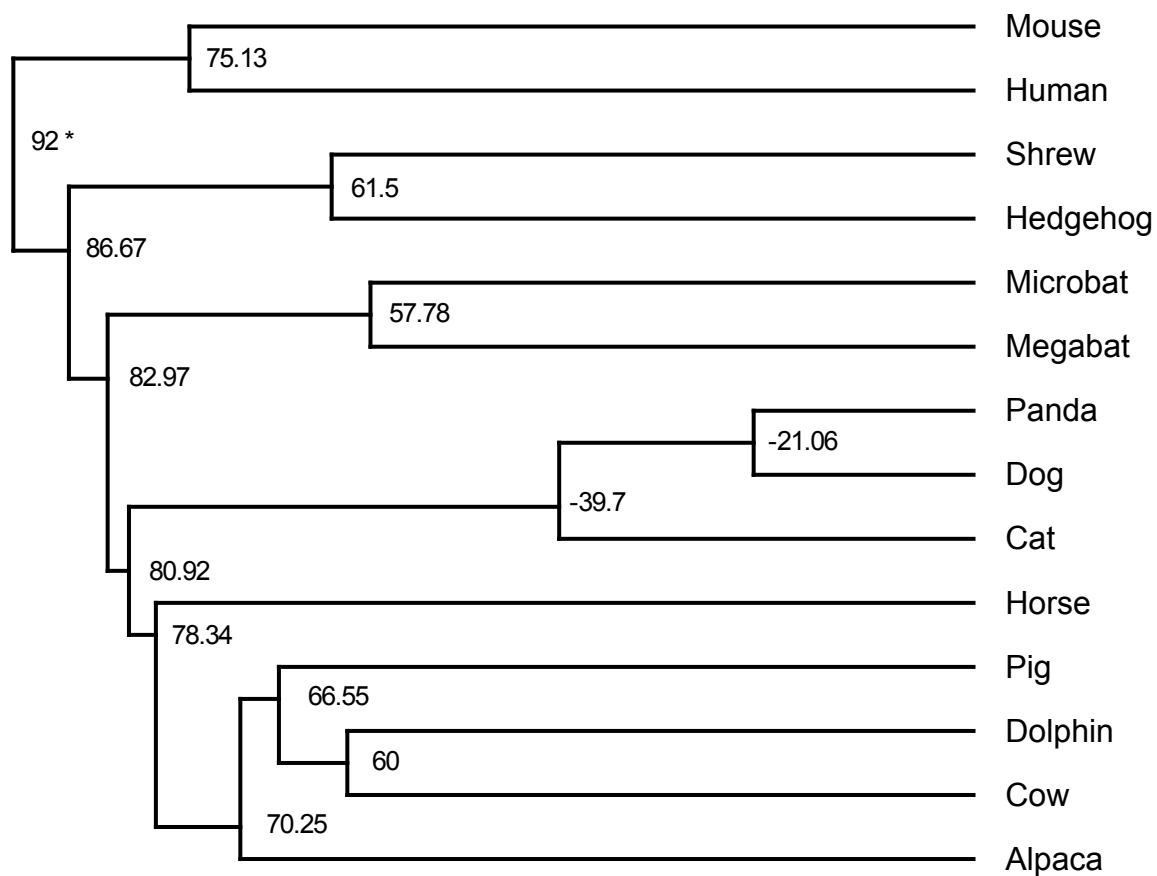

Supplement: Figure S1 — Chronogram showing the estimated times of divergence. (PDF) [file pone.0028199.s001.pdf]
