# Supplementary material for: A Genomic Approach to Examine the Complex Evolution of Laurasiatherian Mammals
Source: PLoS One. 2011 Dec 2;6(12):e28199. doi: 10.1371/journal.pone.0028199 (PMC3229520; doi:10.1371/journal.pone.0028199)
Supplement: Figure S2 — Alignments of all informative retroposon insertions found in this study. (PDF) [file pone.0028199.s002.pdf]

Laurasiatheria monophyly

B-2016 L1MC1

```
cow     GTGTCAATATTTTGC-TATTTTCTCACTTTTTCACGTATTTTT---------------------------------------------------------TTTTTCAGAGCAGTTTTAGATCTACAAC---ATTGGGAGGGAGTTACAGAGATTTCCCATATA-CCCTTACCCTCACAAATGCATAGCCT-CCTCACTATTG------------------ACCAAAGAT
dog     ATATCAATATTTTGCTTATTTTTTCACTCTTTTAT----------TTTTAA-----------------------------------------AGATTTTACTTTTTGGAGCAGTTTTAGGTCTACAACAAAATTG-GAGGGACATTTAGATATTTCCCATATACCCACTGTCTCCACACATGCATAGCCT-----ATTATCA------------------ACCAAGGAT
bat     GTATCAATATTTTCCTTATTTTTTCAC-TTTTTATACATTTTTACTTTTAA-----------------------------------------AGAATTT-CTTTTTAGTGCAGTTTTAGGTTTACAACACAATTGGGAGGGAGGTACAAATATTTCCCACATACCCCCTTCTTCCACACATGCATGGCCT-TCTCATTGTTA------------------ACTAAGAAT
horse   AAATCAATATTTTGCTTATTTTCTTACATTTTTATTTATTTTTATTTTTAA-----------------------------------------AGACAAT-TTTTTTAGACAAGTTTTAGGTTTACAACAAAATTGAGAGGGAAATACAGAGATTTCCCATATACCCCCTGCCCCCTCACATGCATAACCTACCCCATTATCA------------------ATCAAGGAT
shrew   ATTTCTGCCTTTTGACCATTTTTCTCCGTTTCCCTCTTTTTTCACCTTCTGCCTCTAGTAACCATGTGTCTGATATTTATACTCAAGAGTTTAAGATTTTTTTTGCTTTGTGTTTTAGGTTTTACATATAAGTG---AAATCATACAGTGTTTTACAACATCTGACTTGTTACTGGATTTATAGAACCTAACAAACCAATAGAAAAAG...TAAAAACAGTAAAGAAG
human   ATATCAATAATTTGCTTATTTT---------------------------------------------------------------------------------------------------------------------------------------------------------------------------------------------------------

cow     AAACCTACACTAACACACCATGATCCCCCAAA--------------------TTCACTCAGTGTTGCGCATTCTATGGG----------TTTGGACAAATGTGTAATAACATATACCCTTAGTTATAAGGGTATCACATAGAATATTTTCACTGACCTA-AAAATCCTCTGTGCTCTG-CCTTATTTTGCTTATTTTTTTTTTTA--ATTTTTAGGTAGATATGCAAC
dog     GAACCTACATTAACAAATCATAACCACCCAAAGTCCATAGTTTACTTTAGGGTTCACTTGGTGTTGTACATTTTATGGG----------TTTGGACAAATGTGTAATGACATATAACTATCATTACA---GTATCATAGAGAATATTTTCACTGCCCT--AAAATCTGCAATGCCCTA-CTTTATTTTTCTTATTTTTTAACTTA--ACTTTTAGGTAAATAGGCAGC
bat     GAATCTATATTGACATGTCATAATCAATCAAAGTTCATAATTTACCCTGGGGTTCACTCAGTGTTGTACATTCTATGGG----------TTTGGACAAATGTATGGTGACATGTATCCATTATTACA---ATATCATATAGAGTATTTTCACTGCTCTA-AAAATCCTCTTTGCTCTC-CCTCATCTTGTTTATTTTTTAACTTC--ACTTTTAGGTACATATGCAGC
horse   GAACCTACATTGACACATCATTATCACCCAAAGTCCTTTGTTTACCTTAGGGTTCACTCAGTGTTGTACGTTCTATGGG----------TTTGGACAAGTGTATAATAACACATAGCCATCATTATA---TTATCACACAGAGTACTTTCACTGCCCTA-AAAATCCTCCATGCTCTG-CCTTATTTTGTTTATTTTTTAACTTA--ACTTTTAGGTAGATATGCAGC
shrew   AATCCTACATTAATGCATCACCGTGACCTAAGCACCATA--TTACCTCAGTATTTGCTTGATGCTGTGTATTTGATGTGTGGGTTTACATCGGAATGAATTAGGACAAATGTGTATTGTTTATCAAA---CTGTCATGTGGAGTATTTTTATTACCCTAGAAGAATGTCTATACTCTGACATTTTTGTGCTAATTTTTTAACTTAACATTTTTAGGTAAATATGCAGC
human   ------------------------------------------------------------------------------------------------------------------------------------------------------------------------------------------------------------AA--ACTTCTAGGTTAATATACAGC

cow     CTAATTTTGAAAACAACATCTATATGTATTGTTACTTTATTGACTTTATTATCATTGGATTATTTCTT
dog     CTAATTTTGAAGACAACATCTACATGTATTGTTACTTTATCAACTTTGTTATTATTGGATTATTTCTT
bat     CTGATTTTGAAGTGAATATCTACATGTATTGTTACTTTATTGGTTTTATTATCATTGGATTATTTCT-
horse   CTAATTTTGAAGGCAATATCTACATGTATTCCTACTTTATCAGCTTCATTATCATCGGATTATTTCTT
shrew   CTATCTTTGAAGTCAACTTGTATATGTATTGTTACTTTATCATCTTCATTATTATTGGATTATTTTTC
human   CTCATTTTGAAGTCAACATCTACATGTATTGTTACTTTATCAACTTTATTATATTTGGAGTATTTCTC
```

B-3137 L1MA9

```
cow     CACCTATAATAAGGACTCTTTTC----TTTAGATGACTACTAGGTATCATAA-CCACCAAGGGGAA------TAACATTGTCCTTATCAGATTTCTAACTTTTGGTTTCTTCAAGGGCCAA----GTGAGAAAATAATTAAAAAATAATAC-GTTTATATAACTGAAATTTGCTAAGAGAGTATAACTTAAATATTTTCACCAATAAAAGGTTAAACACTGTTTTTTT
bat     CACCTGTTTTAAGGATTCTTTCC----TCTAGATGACTTCTAGATAGCACAA-CCACCATGGCAAATAAAATTAACATTGTTCTTACCAGATTTCTAATTTTTGGTTTCTTCAGGGGCAAACAGAGTAAAGAAA-AAACTAAAAATAATACTGTTTGAAGAATTGAAATTTGCTAAGAGAGTAGAATTTAAATGTTCTCACCAAAAAAAAAGTTAAAAATA-CTGTTT
horse   CACCTGTTGTAAGGACTCTTTCC----TCTAGGTAACTACTAGG-AGCATAA-CCACTGTGGGAAATAAAATTAACATTGTACTTACCAGATTTCTAATTTTTGGTTTCTTCAGGGGCAAACAAAGTAAAGAAAGAAATTTGAAACAATACTGTTTGTATAATTGAAATTTGCTAAGAGAGTAGAATGTAAATGTTCTCACCAAAAAAAAATA---------CTGTTT
dog     CATCTGTTACAAGGACTCTATCC----TCCAGGAGACTGCTAGGTAGCTTAA-CTACTATAGGAAAGAAAAGTAATATCATCCTTATCAGATTTCTAATTTTTGGTTTCTTCAGGGGAAAACAAAATAAAGAAA------AAAACCGGTACTATTTGTATATTTGAAA-TTGCTAAGAGAGTAGAGCTTAAATATTCTCACCAAAAAAAGTAAAAAAATA--CTGTTT
hedgehogCACCTACTATAATGACTCA--------TCTAGATATTTGCTAGGCAGCAAAACCCACTGTGGTTTC------TAACATTGTTCTTAGCAGA-CTCTCACTTTTGGCTTCCTTAAGGGCAAGCAATTAAAAAAAA---------AGTAATATT-TGTGTATAATTAAAATTTGCTAAAAGCAGAA------------CTTACTACTAAAAAATTAAAAATA--CTATTT
human   TATCTGTTTTAAGGATTCTTTTCTTTGTCTAGATGACTGCTAGGTAG-AAA--CCACTGTGGGAAATCAAGTTAACATTGTCCTTAGCAGATTTCTAATTTTTGGTTTGTTCAGGGGCAAAGTTAAAGAAAAAA--AATTAAAACC-----------------------------------------------------------------------AATA--CTGTTT

cow     TAAATT-AGAATTATTAAACATTCATACAGAGTAGCAATAACATAGACAACTTGA-GGGAGA
bat     TAAATT-AGAATTATTAAACATTTATACAGAGTAATAAT---ATATATAACTTAAGGGGAGA
horse   TAAATA-AGAATTATTAAACATTTATACAGAGTAATAATGACATATGCAACTTGA-GGGAGA
dog     TAAATT-AGAATTGTTAAACATTTATACAGAGTGATAATAACATATACAGCTTGA-GGGAGA
hedgehogTAAGTT-ATAA---------ACATATACAGAGTAGTCCTAACACACAAAACTCGA-GGGAAG
human   TAAATTTAGAATTATTAAACATTTATGCAGAGTAATAGTAACATAAACAACTTGA-AGAAAA
```

D-36141 L1MA9

```
cow     AGCTGCATCTCAAATGTAGCAAA-ACAGCCATTGACAGTAAAGTCAGATAGGTACAAAGTGTGTTGAGGTCAGAAATAGTTTTTGAATT-----AGATGGAGATGGGGTG---GGGAAATGGGGAGAAGTTGGTAAAAGGGTACAAACTGTCCATTA--TGAGGTGAATGAGATCTGTGGATCTAATGTA-------------------GTTAACACTGTATTGTATG
horse   AGCTGCATCT-AAATGTAGCTAA-ACAGTCACCAACGACGAAGCCAGGCAGCCTTGGTCAGTTTCAAGGTCAGAAATAGTTTTTGGCTTAAGACAGATGTAGATGGGAGTCG-GGGAAATAGGGAGAAGGTGGTAAAAGGGTACCAACTTTCAGTTA--TAGGTTGAATAAGGTCTGAGGATCTAATGTAGAACATGGTGACTATAGCTGATAACACTGCATTGTATA
dog     AGCTGCATCTCAAACGTAGCAAA-ACAGCCATCAGCAATAAAGTCAGGCGGCTGCCGTCAGTCTTGAGGTTAGAAATCATTTCTGACTTAAGACAGATGTAGACGAAGGTGG------AGAAACAGAAGTGGATAGAAGGACACAGACTTACAGTGA--CAAGATGGATAATGTCTGAGGATTTCATGTAAAACATGGTGAGTTTTGTTGATAATACTGTATTGTCTA
microbatAGCTACTTCTCAGCTGTAACAAA-ACAGCGATCAGTAATAAAGTCAGGCAGCCACAGTTGTCCTGAGA-------------TTTAGTTTAAAACAGATGTAGACGGGTGATGTGGAAAATAGGGAGAAGTTGGTAAAAGGGTACAGACTTTCAGTTA--TAAGATAAATAAGGTCAGAGGATCTAAGGTTTAATATAGTGACTATAGTTGATCACACT--ATTATAGA
shrew   AGCAGTACCTCAAATGCAGCCAG---------------------------AATACAGACAATCTGGAGGTCACAAATGGGTTCTGATTT-TGATGGATGTTGATGGGGATAG--GAGAAATAGGAGGAGTGAGGAATTGTCTACAAATTTTCAGCTACCTAAGATGAATAAAGTCTGAG----TAATGTATAATGTGGTGA-TATGACTGATTGCACT----TGTATA
human   AGCTGCATCTCAGAGGAAGTGAA-ACAGCCACCAACAATAAAGCCACAGTG----------TCTTGAGGTCAGAAACAGTTACTGGCTTAACACAAATGTA------------------------------------------------------------------------------------------------------------------------------------------
mouse   AGCTGCCTTTCAAATGGAGCAAACAAAATAGCTACTAACCAAGCCAGGCAACCTAAGTCAATGTTGAGGTCAAAAATACTTGTTGACAGGAGACAGATGGATA----------------------------------------------------------------------------------------------------------------------------------------

cow     CTTGAAATTTGCTA--AGGGTAGAACTTAACATATTCTCAG-CAAAATAAAGGTAGGGATAAATATGTAAGGTGACAGATATGTTAATG-------------------AAATC-AGTGGAGGCATCACTTCATAATATATACATG-TATCAAATTATCACATTATACATTTTAAATGTCTTGCCATTTTGTCAGTTAA-AATACCTCAGTAAAGCTGAA------AAA
horse   ATTGAAATATGCTAAGGGAGTAGAACTTAA-ATGTTCTCTGCCCCAAAAAGGGGGGGGCATAATATGTGTGGTGATGGATGTGTTATTC-------------------AACTCGAGGGGGGAAATCCTTTCACAGTATACATGTA-TATCAAATCATCACATTGTACACTTTAAATATCTTACAATTTCACCTGTCAGTTATCTCTCTATAAAGCTGAAAAAAACAAA
dog     ATTGAAATTTGCTGAAAGGCCAGAACTTAG-ATGTTCTCAG---AAAAAAAAAAAATGGTCAGTATGCAAAGTGCTGGATGTGCTG-----------------------ACTC-CATGATGGGAACCCTTTCATATATACGTGCA-TGGTAAATAATCACACTGTACACCTCAAGTATATTACAGTTTT----GTCAATTATGCATCAATAAAATTGGA------AAA
microbatGTTGAAATTTGCTAAGAGAGTAGAACTTGA-ATGTTCTCAG------CAAAAGAAGGGGGAAATATGTGAGGTGATGGATCTGTTA-----------------------ATTC-AATGGGGGAATCCTTTCACAATATGTTATGT-ATGCAAATCATCACATTGTACACTTTAAATATCTT-CAGTTTT----GTCAATTAT-CCTCAATAAAGCTTTA------AAA
shrew   ATTGAAATTTGCTCTGAGAGTAGAACTTAA-ATGTTCTCAC-----AAAAAGGATGGGGTAAATAAGTGGGGGATAGATTACCCTGATGTTATTAACACAATATGCATTAAGCTAATGGAGACATCTTTTTGTAATATGTACTCATTGTCAAATCATCACTCTGAACATTTTCAGTAGCTTATCATTCTGTTTGTTAGTTAGACCTCAAGAAAGCTGGA-------AA
human   ----------------------------------------------------------------------------------------------------------------------------------------------------------------------------------------------------------------------------------------------
mouse   ----------------------------------------------------------------------------------------------------------------------------------------------------------------------------------------------------------------------------------------------

cow     AAGACAAACGTACATGTCCCTCTGAGCTGT---------------------------------GTTTCTCCAACTTAGGACAACTGCAAGTATGTGCCAAATTCTGGCCAAGAAGATGCAGACAGGGATGGCATTGGAG
horse   AAGACAGATGTGGATTTCTCTCTGAGCTGT---------------------------------GTTTCCCCCCCTTAGGACAACTGCAAGTATGTGCCAAATTCTGGCCAAGAAGATGCAGACAGAGATGGCATTGGAG
dog     AAGGT---CAGAGACGTCTCTCTGAGCTGT-----------------------------------TTCTTCTCCCTAGGACAACTGCAGGTATGTGCCAAATTCTGGCCAAGAAGATGCAGACGGAGATGGCGTTGGGG
microbatAAGACATTTGTAGATGTCTCTCTGAGCTGT-----------------------------------GTTTCCCCCTTAGGACAACTGCAAGTACGTGCCAAACTCTGGCCAAGAAGATGCAGACGGAGATGGCATTGGCG
shrew   AAAATGAAGATAGTTGTTTCTCTGCATTTTCTATTTTTTTTTCTTTTAATTTCCCCTGAATTTTCTTTTCTCCCTTAGGACAACTGCAAGTATGTGCCAAATTCTGGCCAGGAAGATGCAGATGGAGATGGCATTGGAG
human   -----------GGATGTCACTCGGAACTGC----------------------------------ATTTTCCCCCTAAGGACAACTGCAAATATGTGCCAAATTCTGGCCAAGAAGATGCAGACAGAGATGGCATTGGCG
mouse   -----------GAGCATGACTCTGAACTGT-----------------------------TCCCCTCCCCCCCATCAGGATAACTGCAAGTATGTGCCAAACTCTGGCCAGGAAGATGCAGACAGAGATGGCATCGGAG
```

C-4642 L1M4

```
cow     AAGAAGGGATATTTTTAATGAAAGAATGTATATAAAAGAATTCTTGTTAAATTTAAACG--------AAAACA-AATTCCAGTAGTGAGAGATTACTAT-GTGGTTCCT-GGTGTCTGAGCGTGCTCTCTGTGC----------------CTCCCTGTGAGCCTCTGCCAGTGAACCCTTCACGCTAATGCGGAGAGGAGTTTCAAGAAGCCATGGGCTCTATTTTG
horse   AAAAAGGAATATTTTTAATAGAAGAATGTATATGAGAAATTCTTATTGA-ATTTAAATA--------AAAACAGAATTTCAGTAAGAAGAAATTAGTATTGTGGTTCATGATTCTTTGATCATATTCTCTGTGCTCTGTCTGCTTGAAC-CTCCCTGTTAGCCCCTGCCAAAGATCCTTTTATATTAATGGAGAGAGGAATGTCAGGAAGTCGTGGGCTTTATTTTG
dog     AGAAAGGAATATTTTAAATGGAAGAATATGTATGAAAATAAATTCTTAA-ATTTAAATA--------AAAACAATATTTCAGTACTAAGACGTTAATGTAGTTTGTGAT-TTTTTTTGAGCACGTTCTTTGTATCCTGTCTGCCTGAAT-CTCCCTGTTAGTATCTGCCAATGATCTCTTTACATTAATGGAGAGAGGAATTTCAGGAAGTCATGG-----ATTTTG
megabat AAAAAGGCCTATTTTTAATAAAAGAATGTATATGAGAAAATTTTTCTTA-ATTTAAACA--------AAAACAAAATTCCAATAACAAATATATTGTA--------GTCATGATTTTTAGTATGTT-----AAGTCTATCTACTTGAGCATTTTCTGTTAGTTCCTACCAGTGATCCCATTACATTAATGGAGAGGGGAAT-TCAGGAAGTCACAGGCTTTATTTTG
hedgehogAAAAGGAATTACTTTTAGTAAAAGAATATACAAGAGAAGGATTTTCTTATCATTAAACATA/../ATATAACAGTATTCTAATAGTAAGACATCAGTATTTGATTGATGATATTTCTGAGCATGTTTTCTTTGCTAT-------------CTCTCTTTTCGCTCCTTCCAGTGATTCCTTTGCATTGGT----AGAGGAATTTTATGAATTAATGGACTTTATTTTG
human   GAAAAGGAATATTATTAATGAAGGAATGTATACGAGAAAATTCTTCTTA-AATTTAACA-----------------------------------------------------------------------------------------------------------------------------------------------------------------------------

cow     ACAATTCCCTTAATTGTTGAGTTATTATATATGT-------------------------ATAACATTATGTATTAATGTCATTTATTTCTCTTTCTTCATTTGTCAGTTCACCGAAAAACTCAAGAAGGTTCAGCAATTGAGATGACT-CCAATTGAAGCAGATTTCTCTT
horse   ATAATTTCCTTAATTGTTCAGTCATTATATAT---------------------------ATAAAATTATATATTAATGCCATTTTCTCTTCTTACATCATTTGTCAGTTCACCGAAAAACTCAAGAAGGTTCAGCAATTGAGATGACT-CCAATTGAAGCAGATTTCTCTT
dog     ACAATTTTCTTAATTGTTTAATCATTATTTGT---------------------------ATAATATTATATATTAATGCCATTTTCTCT-------TCATTTGTCAGTTCACCGAAAAACACAAGAAGGTTCAGCAATTGAGATGACT-CCAATTGAAGCAGATTTCTCTT
megabat AC-ATCATCTTAATTGTTCAATTATTATACAT---------------------------TTAGTGTTATATATTAATGTCATTTTCTCTTTTTATTTTATTTGTCAGTTCACCGAAAAACTCAAGAAGATTCTGCAGTTGAGATGACT-CCAATTGAAGCAGATTTCTCCT
hedgehogACAATTTGTTTAGTTGTTCAGTCATTGTATATGTTGTTATATATTGTGCACCATATAATATATATATTATATTAATGCCAGTTCACTTTTTTTC-ACATTTATTAGTTCACCGAAAAACACAAGATGGCTCAGCGATTGAGATGACTCCCAATTGAAGCAGATTTCTCTT
human   ---------------------------------------------------------------------------TAATACCATTTTCTGTTTTTATTTCCTTTGTTAGTTCACCAAAAAACTCAAGAAGGCTCAGCGATTGACATGACT-CCAATTGAAGCAGATTTCTCCT
```

CCCP-clade monophyly

B-554 - L1MA9

```
cow     ---TGGTCCAGCTAAGCA-TATTC-ACAGACCTGACCTATAATAATATTAACCTTTATGTTTATAGTCTTAGTGTAATTGATGATTTTCAGTAATATTTATATAATATTAATTTCTTAGGAGAAA-----------ATAAGCCATATCAAGATATAATAGACACATAACATTGTATTATTTTAAGGTATACAACATAA---------TTAGTATATGTATATATTGTG
horse   AGATTGTCCAGCTGAGCAGTAACA-GTAGACTTGATATATAATAATATTAGCTTTTATGTTTATGGTCTTAATGTGTATGATAATTGTCAATAATATTTGTAAAATATTAATTTTATTTAGGTGAAAACTAA----AATAAGCAAATTAAGATATAATTGATATAAAACACTGTATTAGTTTAAGGTGTACAACATAGTGATTTGATTTGATATATGTATATGTTGTG
dog     TGCTTGTGCAACTGAGCAATATCA-ATAGACTTCATCTA---TAATATTAGCTTTTATATTTATAATCTTTATGTACACAATAAATTTCAATAATATTTATAAAAT----ATTTTCTTTAGATGAAAACCTCAATAAATAAGCAAATTCAGACATAATTTACATATAACA---TATTAGTTTAAAATATACAGCAGAA-----TGATATGATGTATGTATATATTTTG
microbatAGATCATCTAGCTG-GTAGTATTA-ATAGACTTGACCTATAATAG---TGACTTCTATATCTCTAGTCTTAATGTATATGATAATGTTCAGTAATGCTTATAAAATATTAACTTTCTTTAGAGGAAACCTTTTTA-AGTAAGCAAATTAAGATATAACCAGCACATGACTTTGTATTAGATTAAGTTGTACAGCATCA-----TGGTTTGAAATATACATATATTGAG
megabat AGGTGATCCAGTTGAATAGCATCA-ATAGACCTGACTTATACTAATACTAACTTTTATATCTGTAGTCAATGTAT-----ATAATTTTCAGTAGTATTTATAAAATATTAATTTTCTTTAAGAGAAAACCTTAGTAAATAAACAGATTTA----------------AGATTTGTGTTAGTTTAAGATATACAACATAA-----TGATTTGATATATGTATATATGCTG
shrew   AAGTTGTACAACTGACAACCTGTATATAGGCCTGAACTA---TGATATTAGATTTCATATTTACTGTCTTAGTACACAAAATATTCATTAATA-------TGGAATGCT-GTTTTATTTATATTAAACCTT-----AATAAACAAATTAAGATATAACTGAC--------------------------TATAGGATAA------------------------------
human   ATGTTGTCTAGCTGACCAGTATCA-AAAGAACTGACCTATACTAATATTAAC-TTTATATTTGTAGTC--AATGTATGTGATAATTTTGAGTAATAATT-----ATATTAATTTTCTTTAAGAGAAAACCTCAAT-AAAAAGCAGATT--------------------------------------------------------------------------------
```

```
cow     AAATAGTTAGCACAGTAAGTTTAGTTAACATCACCACACAGT-------CACAGGTTTTTTCCTCTTGTGATGAGGACTTAAAAATCAGTCTAT--TTAATGCTTCATTTCTACTTTTTTGAAAACTCCAAA
horse   AAATTA----CACAGTAAGTTTAATTAACACCTATCACCTCATGTAGTTGCAAATGTTTTTCTTCTTCTTTTGAGAACTTAAAAATAAGTCTACAATTAGTGCTTTGTTTTTAG-TTTTTTAAAAGTCCAAA
dog     AAATGACTGCCACAGTATCTTTAGTTAACATCAG---------------ACTGTTTTTTTTTTTCTTATGATGAGAACTTAAAAATCAATCCAT--TTAGAGCTTTATTTCTAC-TTTTCGAAAACTCCAAA
microbatAGATGATTATACAGTCAATTTTAGTTAACATTCATCACCTCA-------GTAACAATTTCTTTTCTTGTGATAAGAATTTAAAAATCAGTTCAT--TTAGTGCTTTATTTATAC-TTTTTCAAAAGCCCGTA
megabat AAATGATTACCACAATAAGTTTAGTTAGCATGCATCAACTCA------AAGTTATAGTTTTCTTCTTGTGATGAGAACTTAAAAATCAGCTTAT--GTAGTCCTTTATTTTTAC-TTTTTGAAAAGTCCAAA
shrew   -------------------------------------------------CATTGTGTTTGTTCCTTTAGCCTTGCTGCCTGGAACTTAGTTTAC-CTTAAAGCAATGTCCGTTC-TGTATGGACACAAGAAG
human   -----------------------------------------------------------------------------TTAAAAAATAAGTCCAT--TTACTGCTTTTTTTCTAC-TTTTTAAAAAGTTCAAA
```

B-485 - L1MA9

```
cow     CAGTCCATGAAGCTCACTCACCTTCCCTGAGCT--TTATCCTCCTTAAAAC----TCTCTTTCCAGCCATGCAAGGCTATTGTAATGATTAAACTAGGTCATGTATGTTGAAGCACT----------TTTTATTTTTTATTAAAATATATTTGACATGTGGCATTGTATAAATTTAAGGTGTGTGAC----TTATTTGACACAGATTGTATCT----TATAATTGTCA
horse   CAGTGCATGAAACTCACTCACCTCCCCTGAGCT--ATATCCTCATTAAAACGCCATCACTTTCTGGCCACACAAGATTATTGTAATGATTAAATGAGGTTATGTATGTAGAAGCACTATTTATTTTCTTTTATTTTCTATTAAAATACATTTGACATATAACA---TGTAAATTTAAGGTGTACAATACGTTGATTTGATAC-ATTTATATATTG--TAATATTGCTG
dog     CAGTCTCTGAAACTCATCTCTCTACCCTGAGCTCCATATCTTCATTAATACTCCCTCTCTTTCCAGCCATACAAGATTATTGTAATGATTGAAAGAGGTCATGTATGTAGAAGCACT----------TTTTATTTTTTTATTGGGATATGTTCAACATATAGTGTTGTATAAATTTAAGGTGTACCACATGTTGATTTGATTC-ATTGAAATTTGGAACATGATGGCTC
microbatCAGCCCCTGAAACTCACTTATCTCCTCTGAGCT--ATATCCTTACTAAAATTCC--CTCTTTCCAGCCACCCAAGGTCATTGTAATGATTAACTGAGGTCATGTATGTAGAAGCACT----------TTTATTTTTTCATTGAAATATATTTGACATATAGCATTGTGCAAGTTTAAGGTGTATAACCTGTTGGTTTGATAACATTTACATATTG--TAATATTGCTG
hedgehog--GTCCATACAAATCACTTACCTCTTATAACT---GTCTCCTCCTTAAATTTCC-TCTCTCTTCAGCCACCCAAGATTATTGTAGTAATTAAACAAGGTCATATATGTACACGTCCT----------TTTCA------------------------------------------------------------------------------------------------
human   CAGTCCGTGAAACTCACTTATCCCTCCTTAGCT--ATAGCCTCTTAAAA--CCCTTCTCTTTCCAGCCACATGAGATTATTGCAATGATTAAATGAGGTCATAAATGTAGAAGCACT----------TTTTA------------------------------------------------------------------------------------------------
```

```
cow     TCATGGCAATAATTAACTCCTCTGTCATGTCACATAATT-TCATTTCTTTTTGGTGATTGGAATAATTTAAGATCTAGTCTCTTAGCAACTTTGATGATTGTAATAC-----TGTTGTTAGAAGCTCTCGTTAAGCAGCAGAGGGATTTTGAAGATGTATGGTAATATTCTTATAAAATTAAATGTCATCTGCAGTATTTTAAAGTGCATTATTTTCCAGCTTTCAGT
horse   TTGTTGCAATAATTAGCACCTCTGTCACATCACATAATTATCGTTTCTTTTTAGTGGTTGGAATAC-TTCATATCTAGTCTCTTACCAAGTTTCATGATTATAGCACAATATTG-TTTTAGAAGCACCTTTTAAACAGCAAAAGGATTTTAAAGGT-----GTAATATTCTTATAAAATT-GGTGTCACATATAATATCTTAAAATTCATTATTACCCAGCTTTTACT
dog     TTGTAGC----AATAGTACTTCTATCACATCATATGAGC---ATTTCTTTTTA--GGTAGAAATAA-TTAAGATAGTGTCTCTTAGCACATTTGAGGGTTCTCATGCAACATTGCTAGAAGCACTTTTTTTTTAAACAGCAAAGAGATTTTAAAGGTGTATGGTAATACTCTTATAAA--TCGATGTCATGT---ATATGTTCAAATGCATCATTACCCAGCGTTTAGT
microbatTTGTAAC----ATAAGCACCTCTATCATGTCACGTAATT---GCTTTGTTTTAGTGGTTGGAATAA-TTAATATCTAGTCTCTTAGCAACTTTGATGATTATAATATATCATTA-AAAGAGAAGCACTTCT-----AAACAACAGATTTTAAAGATCTATGGTAATAGAATTGTAAAATC-GACATCATATGTAATATGTTTAAATGCATTATCACCCAGCTCTTAGT
hedgehog-------------------------------------------------------------------------------------------------------------------------------------GAAAGGAAAGGCATCTT--AGACCTAAGATAATATTCTTATAAAGTT--------CATGTAATATGCTCAAATGTATTACTGTCAAGT-------
human   -------------------------------------------------------------------------------------------------------------------------------------CACAGCGAAGTTATTTTAAAGAT-----GTAGTATTCTTATGAAAATGACGGTCATATATATTATATTTAAATGTATTATTACCCAGCTTTACTG
```

```
cow     AGTGTATGGGCCATGAACTGAGAA
horse   ACTAGATGGTCTGTAAATTGAGAA
dog     ACTG-------TGTAAATTGAGAA
microbatACTGTATAGTTTATAAATTGAGAA
hedgehog-----ACAGTGTATAAATACAGTA
human   TGTA-----TCTATAAACTGAGAA
```

Carnivora monophyly

D-36629 – L1_Carn3

```
dog     TAAATCAAGCCCAGCTC--TTAAAGGTAGATATATATACCAACTAAAA---GTAAGTTATGACATTTGCAGTCAGAGAAAGACAGATACCATATGATTTCACTCATATGTGGGATTTAAGAAACAAAACAAAAAAAGAAACAAACAA----ACAAAAGTACTCTTAAATACAGAGAACAAATTG--GTGGTTGCCAGAGGGGAGGTGGGGGAGTG-------------
panda   TAAATCAAACCCAGCTC--TTAAAGGTAGATATATATGCCATCTAAAA---TTAACTTATGGCATTTGCAGTCAGAGAAAGACAAATACCATATGATTTCGCGCATATGCAGAATTTAAGAAACAAAATGAACAAAGAAAAAAGCGAGACAAAGAAGAGACTTTTAAATACAGAGAACAAACAGATGTGGTTACCAGAGGGGAGGTGGATGGGGGAAATAGATAAAGG
cat     GAAATGAAACCCAGCTCTTTTAAAGGTAGATATGGATATCATCTAAAA-TGAAAACTTATGACATTTTCAGCT--AGAAAGACAAATACCATATGATTTCACTCATGTG--GAATTTAAACAAACGAACAAAGAGAAAAAGAAAC-ACACCAAAAAAAGACTCTTAAAGACAGAGAACAAACTG--GTGGTTGCCAGTGGGGAGGTGAAGGAAGG-------------
cow     TAAGTCAAACTCAACTC--TTAAAGGTAGGT-TGTGTACCATCTGAAA-GGAGAAGTTACATTAT-------------------------------------------------------------------------------------------------------------------------------------------------------------------
horse   TAAATCAAACCCAACTC--TTAAAGGTGAATATATGTACCATCTAAAA-TGAAAAGCTATGATGT-------------------------------------------------------------------------------------------------------------------------------------------------------------------
microbatGAAGTCAATCCCAACTC--TCATCTGTAGATATTTGAACCATCCAAAC-TTAAAAATTATGATAT-------------------------------------------------------------------------------------------------------------------------------------------------------------------
human   GAAATTAAACCCAGCTC--TTAATGGCAAATATGTGTACCATCTAAAAATCAGAAATTATGATAT-------------------------------------------------------------------------------------------------------------------------------------------------------------------

dog     ----------------------------------------------------------------------------GGGATGGATGAAATAGATGAAGGAGATTAAGAGTACACTTATCTTGATGAGCACTAAGAAATGTATAGAATTGTTCAATCATTATATCATATGCCTGAGACTAATATAACACTGT----TCATTATATTTGAATTAAAAACATTTT------
panda   GGATGAGATAGATAGAGGGGATTAAGAGTACACTTAGATGTGGTTACCAGAGGGGAGGTGGATGGGGGAAATAGATAAAGGGGATGAGATAGATAGAGGGGATTAAGAGTACACTTATCTTGATGAGCACTGGGAAATGTATAGAATTGTTGAATCATTATATTGTACAACTGGAACTAAT-TACCACTGTATGTTAATTATACTTGAATTTAAAAAAATTT------
cat     ------------------------------------------------------------------------------GGTGGAAGAAATAGATAAATGAGGTTAAGGG----CTTATCTTGAAGAGCACTGAGA---------AATCATTGAATCACTATATTGTATGCCTGAAACTAATACGACACTGTGTGCTAACTATACTTGAATTTAAAACAACTTAAAAAA
cow     ------------------------------------------------------------------------------------------------------------------------------------------------------------------------------------------------------------------------------------
horse   ------------------------------------------------------------------------------------------------------------------------------------------------------------------------------------------------------------------------------------
microbat------------------------------------------------------------------------------------------------------------------------------------------------------------------------------------------------------------------------------------
human   ------------------------------------------------------------------------------------------------------------------------------------------------------------------------------------------------------------------------------------

dog     -----------TTTTGGCTCTGAAACTATATTATTGTACATTCAGGCCTC--CCCCC
panda   -----ATGACATTTTGGCTCTGATAATATAGAATTG-AAATTCAGGCTATA-TTCCC
cat     GAATTATGACACTTTTGGCTCAAAACTGTGTAATTGTAAATTCAGGCCACACCCCCC
cow     -----------TTTTGGCTAT--AACTATGTAATTATAAATTCAGGCCTTG-CCCTC
horse   -----------TTTTGGCTATGAAACTATACAATTGTAAATTCAGGCCATA-TCCCC
microbat-----------TTTTTGCTGTGAAACTATATAATTGCAAATTTAGTTCACA-TCTCA
human   -----------TTTTGGCTATGAAACTATATGATTATAAATTCAGGCCATG-AACCC
```

D-38269 – L1_Carn2

```
dog     TACTGTACGGTGGCTAT-AAAAATCATTTCTTGAGGCCACTAGTAACCTAGTATTTTCTA---------------------------TTTTATTTTTCAAGTTTTTATTTAAATTCCAG----TTAACATACAGTGTAATATTCCTTCCAGGTATAGAATTTAGTGATTCAACACTTCCTTAAAACACTA-GTATTTTTTAATGAAAAATAATTCTCAAAGGCAAGGG
panda   TTTTGTATGGTGGCTAC-AAAAATCATTTCTTGAAGCCACTAGTCACCCAGTATTTCGTATCTTATTTTAT---------------TTTTTATTTTTCAAGTTTTTATTTAAATTCCAGTTAATTAACACACAGTGTAATATTAGTTTCAGGTGTAGAATTTAACGATTC-ACACTTACTTTACACACAG-GTATTTATTGATAAAAAAGAATTCTCAAAGACAAGGC
cat     TTTAGTATGGTGGCTATAAAAAATCATTTCTTGAAGCCACTAGCAACCCAGTATTTTATTTATTTTATTTTATTTTATTTATTGTATTTTTATTTTTCAAGTTTTTATTTAAATTCCAGTTAATTAACATGCAGTGTAATATTAGTTTGAGGTCTAGAATTTATTGATTCAACACTTATATACAACACCAAGTATTAATTAATGAAAAAGAATTCTGAAAGGCAAGGC
cow     TTATTTCTTATGGCTGT-AAAAACAATTTCTTGAAACCAGTAATAGCACA---------------------------------------------------------------------------------------------------------------------------------------------GTATTTATTAATGAAAAATAATTCTCTAAAACTCAGC
horse   TTTTATCTTATGGGTGT-CAAAATCATTTCTTGAAGCCGCTAATAACCCA---------------------------------------------------------------------------------------------------------------------------------------------ATATCTATTAATGAAAAATAATTCTCTAAGGTAAGGC
megabat TTTTGTCTGGTAGCTAT-AAAAATCATTTCTTAGAACCACTACTAACCCA---------------------------------------------------------------------------------------------------------------------------------------------GTATTTATTAATGAAAAATAATTCTCTAAGGCAAGGC
human   TTTTGTCTGATGACTGT-AAAAATCATTTATTGAGGCCACTAATAACCCA---------------------------------------------------------------------------------------------------------------------------------------------ATATTTATTTATGAAAAATAATTCT-TAAGGCAAGGC

dog     TATGATGAATATATATA
panda   TATGATGAATATTTATA
cat     TCTGATAAATATATGCA
cow     TATGATAAATATACATA
horse   TATCAT-AATACATATA
megabat TATGTTAAATATACATA
human   TATG----GTATATTTA
```

D-41109 - L1_Carn3

```
dog     GCAGCAAGCCATATGAAAAGGTCTTTT--TTTTTTTTCTTTT-----GTAATA-TTTTAACCACTTTAAAAAAATTGTAATTCTAGTGTAGTCAACATACAGTGT----TATGTTAGCTTCATGTGTACAATATAGTGATTCTACAATTCTGTATATTACTCAGTGCTCGTCAAGAGAAGTGTACTCTTAATTCCATTCACCTATTTCACCCATCTCCCTGCCCACCT
panda   GCAGCAAGCCATATGAAAAGGTCTTTTTTTTTTTTTTCTTTTTTTTTGTAGCA-TCTTAACCACTTAAAAAAATTTTTAATTCCAGTGTAGTTAAGATACAGTGT----TATATTAGCTTCAGGTGTACAATATAGTGATTAAACAATTCATTGTATCACTCAGTGCTCATGAAGATAAGTGTACACTTAATTCCCTTCATCTGTTTCACCTATCTCTCTGTGCACCT
cat     GCAGCAAGCCATATGAAAAGGTATTTTGTTTTTTATTTTTTG-----GTAGCATTTTTAACCACTTAAAAAATGTTTTCATTGCAGTGTAGTTAACATACAGTGTCGGCTACATTAGCTTCAGGTACACAGAGTAGTGACTCAGCAGTTCTGT-------TACGTGCTCATCAAGAGAAGTGGACTCTTAATCCCATTCACTTACTTCCCTCATC-CCCCGCCCACCT
cow     GCAGCAAGCCATATGAAAAGGTCATTT--TACATACTTTCTT-----AGAGCA-TGTA--------------------------------------------------------------------------------------------------------------------------------------------------------------------------
horse   GCAGCAAGCCATATGAAAAGGTCATTT--TTAATACTTTTAT-----ATAGCA-TGTAA-------------------------------------------------------------------------------------------------------------------------------------------------------------------------
microbatGCAGCAAGCCATATGAAAAGGTCATTT--TAAGTATTTTCTT-----ACAGCA-TGCAA-------------------------------------------------------------------------------------------------------------------------------------------------------------------------
shrew   GCAGCAAGCCATATGAAAAGGTCATTT--TTGATACTTTACA-----GTAGTA-TGTGA-------------------------------------------------------------------------------------------------------------------------------------------------------------------------
human   GCAGCAAACCATATGAAAAGGTCATTT--TTAATACTTTGTT-----GTAGCA-TGCAAGTAATAGTTATCAGCTGTGTTTTGGA-----------------------------------------------------------------------------------------------------------------------------------------------
```

```
dog     CCCCTCTGGTAA----CCATCTATTTTCTATATTAAAAGTTTGTTTTTTGGTTTGTCTGTAAAAGGTTATCTTTAGTGTTTTTTTTTATAAGTAGTTATTGTAAGTAGTTATTTATAAGTAGTTATTAAGGATGTTTTGGGGGAAAAAAGAGGTTTTGAAAATATGTTAGGTGTATTGAATTAGTTTC----
panda   CCCCTCTGGTAA----CCATCTTTTCTCTATATTAAGAGTCTGTTTCTTGGTTTGTCTAGAAAAGGTCATTCTTATTGCTTTTTTATAGTAGTAA------------------AATACGTAGTTAACG------------------AAGGTTGTTTTGAAGATAGGTTAGGTGGATTGATTTGGTTTCC---
cat     CCCGTCTGGTAATTATCTATTTGTTCTCTATATCAAGAGTCCGTTTTTTGGTGTGTCTTGAAAAGGTCATTCTTAATACTTTCTTACAGTAGTAA------------------AATAAGTAGT-----------------------AACGGTATTTTGAAAATGGGTTAGGTGTATTGATTTGGTTTCCAAT
cow     ---------------------------------------------------------------------------------------------------------------------AATAGTTAAG-------------------AAGGATGTTTTTAAAATAGGTTAGATGTATTGATTTGATTTT----
horse   -----------------------------------------------------------------------------------------------------------------AATAAGTAGTTAGG-------------------AGGGATATTTTAAAAATAGGTTAGATGTATTAATTTGATATCC---
microbat-----------------------------------------------------------------------------------------------------------------ATTAAGTAGTTCTG-------------------AAGGATATTTTGAAAATAGGGTG-----------TTAATTTCA---
shrew   -----------------------------------------------------------------------------------------------------------------AATAAGAAAATG---------------------------ATGGTTTGGATTGGTTCTGTATACTGAAATAATTTTC---
human   -----------------------------------------------------------------------------------------------------------------AATAAGTAGTTATG-------------------AGCTATGTTTTGGAAACAGTTTAGCTG-AGTGATTTGATTTCC---
```

D-42560 - L1_Carn5

```
dog     CTGCAAAAGTTTTGTAGCAGTACCTCA-TTATATTTTATGAAAAATAGTTGAAAATATGACAGATTGATGATGT--TGTCCTCCTAAAATTTTTAGTTTATTCATTTTTATTTTTATTTATTGAGTATAGTTGACACACAATATTAC---TTTAGTTTCCAGCGCACCACATAGTGATTCATCAGTTCTATACAGCCAAGGGTGACTACCATCTGCCACCACATCATG
panda   TTG--AAAAGTTCATAGCAATACCTCA-TTACATTTTATGGAGAGTAGTTGAAAATATGGCAGATTGAGTGCTTTCTGTCTTCCCAAAATTTTTACTTTATTCACTTTTATATTTATTTATTGAGTATAGTTGACACACAGTGACACACAGTAGTTTCAGGGTGTACGACATAGTGATTCATCAACTCTATACTGCCAAGTGTAACTACCACCTGCCACCACATAATG
cat     TTGCAAAAGTTTCATAGCAGTACCTCATTTCCTTTTTATGGAAAATAATTGAAGATATGGCAGATTGATGGCTTTCTGTCTTCCCCCCGTTTTTATTTTATCTATCTTTATTTTCGTTTATCTTTTGAGTATAGTTCGCACCAGTGTTGCATGGGTTTCAGGTGCACAACATAGTAATTCATCAACTCTGTACCG-CAAGTGTAACTACCACCAACC----------C
cow     TTGCAAAAATTTCATAGCAGTAACTCA-TTATATTTTATAGAAAACAATTGAAAACATGGCAGATACATGGCTT----------------------------------------------------------------------------------------------------------------------------------------------------------
horse   TCGCAAAAGTTTCATAGCAGTACCTCA-TTATATTTCATGTAAAATAATTGAAAACATAGCAGGTTGATGGCTT----------------------------------------------------------------------------------------------------------------------------------------------------------
megabat TTGTAAAAGTTACATAGAAGTACCTGA-TTATATTTTATGGAAAATGAT--GAAACATAGCAGATTGATGGCTA----------------------------------------------------------------------------------------------------------------------------------------------------------
shrew   TTATACAA-TTTCATAGGGTTATGTTA-ATATATGTTATGAAAAATATTTGAAGACATGGAAAGCTTGTGGTTT----------------------------------------------------------------------------------------------------------------------------------------------------------
human   TTGCAAAAGTTGTGTATCAGTAACTCA-TTATACTTTATGGAAAATAGTCAAAAACATGGCACATTGATGGGTT----------------------------------------------------------------------------------------------------------------------------------------------------------
```

```
dog     CTATTTCAGTACCATTAACTATATTCTTGATGCTGTACCTTTTATTACTGTGACTTACTCATTTCAT-ACTGGAAGCCAGTATCTCCCAGGTCCGTTCACTTGTTTTGCTGATACCACCACCCACCCGCCTCTCCCTCCTGTCTTCCCACAA-TTTTAGAGCCTGTAAAACTCTAAGTGGACATTTGAGAAA
panda   CTATTACAATACCACTGACTGTATTCCCTATGCTGTACCTTTTATTCCTGTGGCTTACTCATTTCAT-CCTGGAAGCCTCTATCTCCTAACCCCTTCACCTGTTTTTGCTGATTCTCCCG-------CCTTCTCCCTTCTGTCTTCCCACAATTTTCAGAGCCTGTAAGACTCGAAACAGACATTT--TTAG
cat     CTGCTACAACACCACTGACGATATTCCCTACGCTGTACCTTTGACTCCTGTGATTTCTTTACTCCCTACCTGGAAACCTATCTCTCCCA----CTTGCCTTCATTTTGCTGATTCCCCCA-------GCTCCTCCCCTCTATCTTCCCACAATTTTTAGAGCCAGTAAGACGCCAAGCAGTCATTT---TGG
cow     -----------------------------------------------------------------------------------------------------------------------------------------TCTGTCTTCCCACAACTTTTACAGACAATAAAACTCTTAGCAGTCATTT---TGG
horse   -----------------------------------------------------------------------------------------------------------------------------------------TCTGACTCCTGACAATTTTTAGAATCAATAGGACTCTAAGTAGCCATTT---TGG
megabat -----------------------------------------------------------------------------------------------------------------------------------------TTTATCTTCCCACAATTTTTAGAGCCAACAGGATTCTAAGCAGTCATT----TGG
shrew   -----------------------------------------------------------------------------------------------------------------------------------------TCTGTCCTCAAACAAGTTTTAGAGCTAATAAAACTCCAAGCCA---------TGT
human   -----------------------------------------------------------------------------------------------------------------------------------------TCTGCCTTTCTACAATTTCTAGAGCCAATAGGGCTCTAGGCAGTCATTT---TGG
```

D-43671 - L1_Carn5

```
dog     TTTTCCATGTGCTTT----------GAGGCCTTTAAAAAGGATCCAAATTGAAAAAAAAAAAAAAAAAAAGGATCCAAATTGTAAAAGCATTGTGTGGTGACAGATGGTAGCTACACTTGTAGTGAGCATAATGTAACACAGAGATGTTGAATCACTCTGTTGTATACCTGAAACTAACAT-AGCATTGTGTGTCACCTACACTCAAAACCACAAAAAAACAAAAAAT
panda   TTCTCCATGTGCTTT----------GAGTCCTTT------------------------------AAAAAAGGATCCAAATTGTAAAAGCGTTGTGTGGTGACAGATGGTAGCTACACTTGGGGTGAGCACAGCGTAACAGAGAGATGCTGACTCACTGTGTCGTATACCTGAAACTAACATAAGCGTCGTGTGTCAGCTCTACTCGCATTAAAAAAAACCCAAAAAAC
cat     TTTTCCATGTGCTTT----------GAGGCCTTA------------------------------AAAAAAGGATCCAAATTGTAAAAGCATTGTGAGGTGACAGATGGTAGCTACACTTGTGGCGAGCATAGCATAA-ATAGAGATGACGAATCACTGTGTTGCGCACCTGAAACTAACAT-AACACTGTGTGTCAACTATACTCGAAT-------------------
cow     TTTCCCATTTTCTTT----------GAGATTTTA------------------------------AATAAATAATCAAAAT----------------------------------------------------------------------------------------------------------------------------------------------------
horse   TTCTCTGTGTGCTTT----------GAGGCCTTA------------------------------AAAAAATGATCCAAAT----------------------------------------------------------------------------------------------------------------------------------------------------
microbatTTTTCCATGTGCTTT----------GAGGCTTTT------------------------------AAGCAATGATTTAAAT----------------------------------------------------------------------------------------------------------------------------------------------------
hedgehogTTTCCCATTTGCTCT----------AAGTCCTTT---------------------------------AAAAGCTCCAGTT----------------------------------------------------------------------------------------------------------------------------------------------------
shrew   GTTTTCCTTTTTTTATGGCTTTGAGGAGGCCTTA---------------------------------AAATGATCGAAAT----------------------------------------------------------------------------------------------------------------------------------------------------
human   TTTTCCATGTACATT----------GAGGCCTTA-------------------------------AAAAAGGATCCAAAT----------------------------------------------------------------------------------------------------------------------------------------------------
```

```
dog     AAAAAACAATAGCAACAGAAAAATGGATCCAAATAGAGAGGTTGTGTTTAGTGGTTAGACCAA-TTTG-----CCATTCCTCCTATTTTCAAGTATTTAGAGCTTTTTT--
panda   AGA---------------AAAAATGGATCCAAGTAGAGAGGTTGCATTTAGTGGTTAGACCGA-TTTT-----CCATTCCTCTTATTTTCAAGTGTTTAGATCATTTTTTA
cat     --AAAAAAAATTTAACAAACAGAAGGATCCAAGCAGAGAGGTTGCGTTTAGTGGTTAGACCAA-TTTG-----CCATTGCTCCTATTTTCAGGTGTTTAGATCCTTTTTAA
cow     ------------------------------------GGAGACTGTGTTTAGTGGCTAGGCTGC-TTTATTTACCCATTCCTCCTATTTTCAAATGTTAAGAGTGTTTTTTG
horse   ------------------------------------GCAGACTATGTTTAGTGGCTAGACTGA-TTTATTTACTCATTTCTCCTATTTGGAAATTTTTAGATTGTTTTTAA
microbat------------------------------------GGAGACTGCATTTAGTGGCTAGACTGA-TTTAATTACCCATTCCTCCTA-TTTCAGATGTTTAGATTGTTTTGAA
hedgehog------------------------------------GGAGACTGTGTTCAGTGACTAGATTGAGTTCATTTACCCATGTTACCTATTATAAAATGTTTGCTAATTTTTAAA
shrew   ------------------------------------GGAGTCTCTGTTTAGTGACTGGATTAG-TTTATTTGCCCAGGCTTCTTATTTTCAAATATTCG------TTTAAA
human   ------------------------------------GAAAACTGCATCTAGTGGCTCTTCTGA-TTTACTAACCCAGTCCTCCTATTTTTAAATGTTTGGGTTGTTTTTAA
```

Cow-dolphin monophyly

C4-520 - L1-2_BT

```
cow      TTTCCCCAAGCTTTTGGTGACTTTT-TTTGAAAA---------------TATG---AATATCTGGTATAAATTGGCAACTTTATTTTTTAAATTTATTTTATTAACATATAGTTGATGTACAA---TGTATCTATTTCTAATGTATAGTAAAATAATTTAGTTATACATATGTACACATATTACTTTTCATATTCTTTGCCATTA-----TTTCACAGGATATTGAATA
dolphin  TCTTCCTAAGCTTTTGGTGACTTT--TTTGAAAA---------------TGTGATTAATAT-TGGTATAAATTGGTA--------------------TAAATCAAAGTATAGTTGATTTACAGTGTTGTGTTCATTTCTACTGTACAGTAAAGTAATTCAGTTATACATATATATAC--ATTCTTTTTCATATTCTTTGCCATTATGGTTTATCACAGGATATCGAATA
alpaca   TTTTCCGAAGCTTTTGGTGACTTTA-TTTAAAAA---------------TATGATTAATATCTGGTA------------------------------------------------------------------------------------------------------------------------------------------------------------------
pig      TTTTCCTGAACTTTTAGTGACTTAA----AAAAAGACCC/.../AGACCCATGAATAGTATCTGGTA------------------------------------------------------------------------------------------------------------------------------------------------------------------
horse    TTTTCCTAAGCTTTTGGTGACTTG--GTTTTAAA---------------AACAATCAATAAATGGTA------------------------------------------------------------------------------------------------------------------------------------------------------------------
dog      TTTTCCTAAGATTTTGATGACTATTTTTTTTAAA-------------ACCATGATAA----------------------------------------------------------------------------------------------------------------------------------------------------------------------------
microbat TTTTCCTAAGCTTTTGATGACTTAT-AGTTTTAA---------------TGTGATTAATATAT----------------------------------------------------------------------------------------------------------------------------------------------------------------------
megabat  TTTTCCTAAGCTTATGATGACTTATTTTTTTAAA---------------TGTGATTAACATATGGTA------------------------------------------------------------------------------------------------------------------------------------------------------------------
human    TTTTCCTAAGCGTTCAGTGACTTTTTTAAAAAAA---------------CATGATTAATATATGGTA------------------------------------------------------------------------------------------------------------------------------------------------------------------

cow      TAGTTTCCTATACTGTACAGTCAGACCTTCTTGTTTATCCGTTCTACATAAGAAATTTTGCATCTGTTAGTCTCAAACTCCTAGTTCTTCCCA-CCCCAATCTCCCTCCCTCTAGGCAATCACAGTTCTGTGCTCCATGTCTGTGAGTCTTAAACAGGCAACTTTATAAACAGTGTGACCAT----------GCTAAGCAAAGTATTTGGGGATTTGTTTGTTTTTAA
dolphin  TAGTTTCCTGTGCTGTACAGTAGGACCTTGTTGTTTATTCATTCTATGTATAACAGTTTGCATCTGCTAATCCCAAACTCCCAATTCTTCCCGCCCCCAACTGCCCTCCTCCTAGGCAACCACAGGTGTGTTCTCTATGTCTGCGAGTATTAAACAGGCAACTTTATAAAGAGTATGACCGT----------GTTAAGCAAAGTGTTTTGGGTTTTGTTTATTTTTAA
alpaca   ------------------------------------------------------------------------------------------------------------------------------------------------------TAAACAGGCAATTTTATAAAGAGTATGACCGT----------GCTAAGCAAAGTGTTTGGGGTT----TTATTTTTAA
pig      ------------------------------------------------------------------------------------------------------------------------------------------------------TAAGTGGGCAACTTTAAAAAGACTGTGACCAT----------GCCAAGCAAAATGGTTTGGGTTTGGTTTATTTTTAA
horse    ------------------------------------------------------------------------------------------------------------------------------------------------------TAAATGGGCAACTTCCTACAAAGTATGACCGT----------GCTAAGCAAAGTGTTTTGGTCTTTGTTTATTTTTAA
dog      ------------------------------------------------------------------------------------------------------------------------------------------------------TAAATAGGCAACTTTCTAAAGCAAAACGTTTTTGGTTTTAAAACTAAGCAGAATGTTTTGATTTGGGTTTATTTTTAA
microbat--------------------------------------------------------------------------------------------------------------------------------------------------------AAGGGGCAACTTTCT--AGAGTTCACCTGC----------GCTAAGCTAGGTGTTTTGTTTTTTGTCTATTTTTAA
megabat  ------------------------------------------------------------------------------------------------------------------------------------------------------TAAATGGGCAACTGTCTAAAGAATTTGCCTCT----------GCCAAGCAAAGTGTTTTAGTTT--------------
human    ------------------------------------------------------------------------------------------------------------------------------------------------------TAAA-GGGCAACT-TCTGAAGAGTATAACTGT----------GTTAGGCAAGATGTTTTGGTTTTTGTTTA-TTTTAA
```

C4-613 – L1-2_BT

```
cow      GTGGCTGGCTCTGGAGACAGAATTTCTACCACCCTGAGATACGACTGAGTTTTCTGCTTGGGGGTTTTCCCTATCTCTGCCTCCAAGAGTCTGGTTGATTTCCAAAACTTTCC---------CCTGTGACTTTTTTTTTTAATTTTTATTGGAGTCTGGTTGAGTTACAATGTTGTTTTAGTTTCAGGTATACAGCAAAGTGAATCAGTTGTACATGTACTTATATAC
dolphin  GGGGCAGGCTCTGGAGTTGGGGTTTCTACCACCCTGACACATGACTGGGTGTT-TGCTTGAGGGTTTTCACTACCTCTGCCACTAAGAGTCTGGCTGGCCTCCAAAACTCCCCTGGGACTTCTTTTTTTTTTTTTTTTTTAATTTTTATTGGAGTATAGTTGATTTACAATGTTGTGTTAGTTTCAGGTGTACAGCAAAGTG-ATCAGTTATACATATACATATATCC
alpaca   GTGGCTGACTCTGGAGGTGGGATTTCTACGGCTCTGATATATGACTGGGTTTT-TGCTCAGGGGTTTTCACTAC-TCTGCCTCCAAGAGTCAGTTTAGCCTCCAGAACTTTCC-------------------------------------------------------------------------------------------------------------------
pig      -----------TGGAGATGGGTTTTCTACGGCTCTGATGAATGACTGGGTTTT-TGCTTGGGGCTTTTCGCTACGTCTGCCTCCAAGGGTCTGGTTGGCTTCCAAAACTTTCC-------------------------------------------------------------------------------------------------------------------
horse    GTGGCTGGCTCTGGAGACGG-ATTTGTGTGGCTCTGATGTATGACTGGGTTTT-TACTTGGGGGT-TTAACTGCCTCTGCCCCCAGGAGTCTGGTTGGCCTCCAGAACTTTGT-------------------------------------------------------------------------------------------------------------------
dog      GTGACTGGCTCTGAAGATGGGGTTTCTATGGCTCTGTTGTATGACTGGGTCTT-------------TTCACAACTTATGCCTCCACAAGCCTGGTT-GCTTCCAAAACTTTCC-------------------------------------------------------------------------------------------------------------------
bat      ------AGTTCTGGAGATGGGATTCCTGTGTCCCTGATGCATTACTGGGTTTG-TGCCTGCGGGTTTTCAGT-------------------------CCCTCCCAAACTTCCT-------------------------------------------------------------------------------------------------------------------
human    GTGGCTTGCTCCGAAGATGGAA----------------ATATGGCTGGGTGTT-----TGGGAGTTTTCACTGCCTCTGTATCTGAGAGTCTGGTTGGCTTCCAAAGCTTTCC-------------------------------------------------------------------------------------------------------------------

cow      ACACTCTTTTTACATTATTTTCCCATATAGATCATTTCAGAGTATTGAGTAGAGTTCCCTATGCTATGCAGTAGGTCCTTATTAGTTATCTGTTTTATATATAGTAGTGTGTATCGTGTGTATATATGACTACTTTTTTATTTTCACACTCTCATGCAGCTCAGAGTCCTAGCTGTGA-CTTTGGGACCCTC
dolphin  AC-TCCTTTCTAGATTCTTTTCCCATATAGACCATTACAGAGTACTGAGTAGAGTTCCCTGTGCCATATAGTAGGTCCTTATTAGTTATCTATTTTCTATATAGTCGT-------GTGTTTATGTGTGACTACTTCTTTATTTTCACACTCTCACTCAGCTAGGAGCACTAGGTGTGAACTCTGGGATCCTC
alpaca   --------------------------------------------------------------------------------------------------------------------------CCTGTGACTACTTGTTTCCTTTCA-----GC---CAGCTAGGTG-------TGTCATCTCTGTGATCATT
pig      --------------------------------------------------------------------------------------------------------------------------CCAGCGACTCCTTACTTATTTTCACACTCTCACTCGGCCAGGAGGGCTTGGTGTGATCTGTGAGATGCTG
horse    --------------------------------------------------------------------------------------------------------------------------GCTGTGACTCCTTTTTTGTTTTCACACTCTCTCTCAGCTAAGAGTGCCCAGTGTGACTTTTGTGATCCTT
dog      --------------------------------------------------------------------------------------------------------------------------CCTATGATTCTTTTTCAATTTTCACTCTCTCA----GCTTAGAGAGCTGGGTATGACCTTTGTGATCCTT
bat      --------------------------------------------------------------------------------------------------------------------------CCTGTGACTCTGTTTTCATTTTCACACTCTCACGC-CCTAAGAGGGCGAGGTAAGACCTTTGTGATCCCT
human    --------------------------------------------------------------------------------------------------------------------------CTTATGACTCCAGTTTTACCTTCACTCTCTCACTC-GGTAGGAATGCAAGGTGTGACCCTTGTGATCCTT
```

C4-627 - L1-2_BT

```
cow      CTTAAAAATAAGTTTGGAATAAATGGCATTAAACGAAACTTAAGCATGAAGCTTTTGTTTCCTAGTTGGTTGG----------TTGGTTTTTTTTTTTGAAGTATAGTTGATTTACAATGTTACGTTAGTTTCAGGTATACAATACAGTGATTCAGTCTTTATATAATATGTAGTTTTTCAGATACTTTTCCCTTATAGGTTATTATAAAATATAGATTATAGTTCCC
dolphin  CTTAAAAATAAGTTTTCAATAAATGGCAGTAAATGAAACTTAAACATAAAGCTTTTGTTTATTAGTTTTCTGGGGTTTGTTTTTGTCTTTGCTTTTTTGAAGTATAGTTGATTTACAATGTTGTGTTAGTTTCTGGTGTACAGCACAGTGAAATCAGTCATATAT-ATATATTCGTTTTCAGATCCTTTTCCCTTACAGGTTATTATAGAATATTGAGTATAGTTCCC
alpaca   CTTAAAAGTAAGTTTTCAATAAATGAC-----ATGAAACTTAAACATAAAGTTTTTGTTTA-----------------------------------------------------------------------------------------------------------------------------------------------------------------------
pig      CTTAAAAATAAGCTTTCAATAAATGGCTTTAAACAAAACTTAAACATGAAGCTTTTGTTTA-----------------------------------------------------------------------------------------------------------------------------------------------------------------------
horse    CTTAAAA-TACTTCTTCAGAAACTGTCATTAACAAAAACTGAAACATGAAACTTGTGT--------------------------------------------------------------------------------------------------------------------------------------------------------------------------
dog      CTTAAAA-TATGTTTTTGTAAA--GGATGTTAATTAAACTTAAACATGAAGCTT-TGT--------------------------------------------------------------------------------------------------------------------------------------------------------------------------
bat      CTTAAAA-TACTTTTTCAAGAAATGGCGTTAAATAAAACTTAAACATGAAGTTTTTGT--------------------------------------------------------------------------------------------------------------------------------------------------------------------------
human    CTTAAAA-TGGTTTTTCAATAAACAGCATTAAATAAAACTTAAACATAAAGCTTTTAT--------------------------------------------------------------------------------------------------------------------------------------------------------------------------

cow      TGTGCTGTGTAGTAGGTCCTTGTTGGTTATCTGTTTTATATATAGCAGTGTTTATTCATTTTTGATACATGATATAGAGCTCAAACCTAAA-TAATTTTTTGACTTTGGTTGATACATGT
dolphin  TGTGCTATACAGTAGGTCCTTGTTGGTTATCTATTTTATATATAGCAG-GTGTATTAGTGTTTGATGCATGAAATAAAGTTCAAATCAAAAATAAGTTTTTGGTTTTGGTTGGTGCATGT
alpaca   ------------------------------------------------------TTAGTTTGTGATGCATGAAGTAGAACTCAGATGTAAATAAGTTTTTTAGTTTTGGTTGGTACATGT
pig      ------------------------------------------------------TTAGTCTTTGATGCATGAAATAGAGCTCAGATCTAAG-TAAGTTTTTTGTTTTGGTTGGTACATGT
horse    ------------------------------------------------------TTAGTTTTTGATGCATGTCACAGAGCTCAAACTTAGA-----GTTTTGGGTGTTGCTTGTATCTGT
dog      ------------------------------------------------------TTAGTTTTAGATGTGTGCCATAGAGCTTAACCCTAGG-TAACATTTTGGTTTTGGTTTATACATGT
bat      ------------------------------------------------------TTGGTTTTTGATGCATGTCATAGCATTCAAACCTAAA---------TAGTTTTTGGTTGTACATGT
human    ------------------------------------------------------TTAGTTTTTGATG-AAGTCATAGAGTTCAAACTTAAA-TAAGTTTGTACTTATT-TAAGTACATGT
```

C4-643 – L1-2_BT

```
cow     ATGCACTGCTTATCAAAGACGTGAGTCCTTTCTGCTGCTCTGTAAAGAATTCTCAGTGCATAACCCCTTTCC------------ATAAAATAT--------------ACACACACACAAAAAGCAGTCTTATGACATGAAAATTC--TCTCTTGTCTTCTATATGTAGTATTGAAACTATTGGAGGATGAGGGGAAGGGATAGTTAGGGAGTATGGGAATGACATGTA
dolphin ATGCACTGCTGATCAAAGACGTGAGACCTTTCTGCTGATCTGTAAAGAATGTTCCATGCAT-ACCCCTTTCC------------ATAAAATATACAGACATATACACACACACTTGAAAAGCAGTCTTATGGCATGAAAATCCTATCTTTTGTTTTCTACGTGTAGTATTGAAATTATTGGAGGGTGTGGGGAAGGGATAGTTAGGGAGTGTGGGATTGACATGTA
alpaca  ATGCACTGCTCATCAAAGATGTGAGTTCTTTCTGCTGCTCTGTAAAAAATGCTCCATGCAT-ACCCCTTATTTTGTTTGTGAAAACAAAATACAGAAACATACATACACATAGACTTGAAAAGCAGTCTTATGGCATGAAAATCCTCTCTT--ATCTTCTTTGTGTAGTATTGAAACTAA------------------------------------------------
horse   ATGCGCTGCTTATTAAAGATGTGAGTTCTTTCTGATGCTCTGTAAAGAATACTTCATGCAT-ACCCCTCCCT------------------GCAAAAACATAC--AGACACATATCCTTAAAGCAGTCTTATAGCATGAAATTCCTATCTT--GTCTTTTGTATGTAGTATTGAAACTAA------------------------------------------------
dog     ATGCACTGCTTATCAAAGATGTGAGTCAGCTT------CTTTTAAAGAATGCTCTGTGCAT-ACCCCTCCCC------------------ACAATAT---------------------------------ACACAAAAGACATACTCCCTACTATCTG---------------------------------------------------------------
microbatATGCACTGCTTATCAAAGATGTGAGTTCTTTTTGCTGCTCTGTAAAGAATATTCCATGTAT-AACCCTCCCT------------------GCAAA---------ACAGACACAATTGATAAGTAGTCTTATAGCATGAAAATCCTATCCCAGATCTTCTGTATGTGCTATTGAAACTAA------------------------------------------------

cow     CACACTGCTGTAGTTTAAATGGATAACCAATAGGGATATACTATATA--ACAAGGAAATCTGCTCAATATTATGGAACAACCTAAATGGGAAAATAAATCTGATAAAGAATAGATACATGTGTATGTATAACTGAATCACTTTGCTGTGCACCTGAAA---CTCACAACATTGTTAATCAACTATACTCCAATATAAAATAAAAAGTATTTTAAAAAGAAAAGAAACT
dolphin CACACTGCTGTATTTAAAATGGATAACCAATAAGGGCCTACTGTATAACACAGGGAACGCTGCTCAATATTCTGTAACAACCTAAATGGGAAAA-GAATTTGAAAAAGAATAGATACATGTATATGTATAACTGAATCACTTTGCTGTACACCTGCAAACTATCACAACACTGTTAATCAACTATACTCCAATGTAAAATTAAAAGTTTTTAAAAAAGAAAGAAACC-
alpaca  -------------------------------------------------------------------------------------------------------------------------------------------------------------------------------------------------------------
horse   -------------------------------------------------------------------------------------------------------------------------------------------------------------------------------------------------------------
dog     -------------------------------------------------------------------------------------------------------------------------------------------------------------------------------------------------------------
microbat-------------------------------------------------------------------------------------------------------------------------------------------------------------------------------------------------------------

cow     ATTGGGATCTGGTCATCAAGATTCTTTTTATCTCTCTGTGCAGTACCCCTGGCCAAAG--TTTTCACAA--GTC-TACCTGAACTCTTCGAAATGTCTTCAAGCTCACTTTATCTTCTCTCTAGGTTCCTGCTACCCGTGAAGT
dolphin -TTTGGACCTGGTCATCAAGATCCGTTTGATCTCTCTGTGTAGTACCCCTGGCCAAAG--TTTCCTAAC--TTTGTACCTGGACTCTTTCAGATGTCTTCAAGCTCACTTTATTTTCTCTCTAGGTTCCTGCTACCCGTGAAGT
alpaca  -TTGAGACCTGGTTATCAGGATCCTTTTTA--TCTCTGTGCAGTACCCCTGGCCAG----TTTCCTAATG-TCTACACGTGGACTCTTTCAGATGTCTTCAAGCTCACTTTATCTTCTCCCTAGGTTCCTGCTACCCGTGAAGT
horse   -TTGGGACCTGGTCATTAAGATCCCTTTTATCTCTCTGTGCAGTACCCCTGGCCAGAG--TTTCCTAGCA-TCTACACTTGGACTCTT--AGATGTCTTTAAGCTCACTTTATCTTTTCTCTAGGTTCCTGCTACCCGTGAAGT
dog     -----------------------------------CTGTGCAATA--CCTAATCAAAG--TTTTGTGGCATTCTATACCTGAACGCTTTTAGAAGTCTTCAAA--CACTTGATCTTCTGTCTAGGTTCCTGCTACCCGTGAAGT
microbat-TTGTAACCTGATTATCAAGA-------------TCTGTGTGATACCCCTGATCAATGTTTTTCTTAGCA-TCTACACCTGAACTCTTTTAGATGTCTTCAGACTCACTTTACCTTCTCTGTAGGTTCCTGCTACTCGTGAAGT
```

C4-1124 – L1-2_BT

```
cow     TTAACCGTGCTAATGGGAAGGTAAGAATGCTAACCCTGGAGACCTGTAATGACAGTTGTTTCAATTGGGAC----------AGTTTTCTGAATGTTAAAATCTGCATTGTAGAAA------GACTCAGACAGAAAACAAA--CACTGTTATCAAAAGGGAAAGGGGCGAGGAGGGATACATTAAGAATATAGGGTTAACAGATACACACTACTTTATATAATATAGAT
dolphin TTAACCGTGCTAATGGGAAGGTAAAAACGCTAACCCTGAAGACCTGTAAGAAGTGTT-TATCAGTTGGGAC----------AGTTTTCTGAATGTTAAAATTTGCATTTCGGAAATAGACTCACAGACATAGAAAACAAATTTATGGCTACCAAAAGGGAAAGGGGTGGGGAGGGGTAAATTAGGAATTTGGGGTTAACAGATACACACTACTAAATATAAAATAGAT
alpaca  TTAACCGTGCTAATGGGAAGGTAAGAACGCCAGCCCTGAAGACCTATAGGAAGTGTT-TATCGCTTGGGAC----------AGTTTTCTGAATGTTAA-------------------------------------------------------------------------------------------------------------------------
pig     TTAACCGTGCTAATGGGAAGGTAAGAACGCTAACCCTGAAA--CTGTACAAAGTGTT-TATTAGTTGAGAC----------AGTTTCCTGAATGTTAC-------------------------------------------------------------------------------------------------------------------------
horse   TTAATCGTGCTAATGGCAAGGTAAAAATGTTAATCCTAAAGACATATGAGAGGTGTT-TATCAGTTGGGACAGTCATCTTTAGTTTTCTGAATGTTAA-------------------------------------------------------------------------------------------------------------------------
dog     TTAACCGTGCCAATGGGAAGGTAA-CACATGAACCCTGAAGACCTGTAAGGAGTGTT---------GGGACAGTCATCTTCAGTTTTCTGATTATTCA-------------------------------------------------------------------------------------------------------------------------
bat     TCAACCGTGCGAACGGGAAGGTAAAG--GCTCACCCCGCAGACCCG-GGGACGCGTT-TGTCGGCTGGGACCGTCCCTGT-CATCTTCCGAATGTTAA-------------------------------------------------------------------------------------------------------------------------

cow     AAACAGCAAATTCCTACTGT-------AAGGAACTATATTCAATGTCTTGTAATAATCTATAATGGGAAAGAATCTGAAAAGAAATATGTACACA-------------GAGTGTATGTATAACTGGATCACTTTGCTGTATGTTTGAAACTAAC---ACATTATAAATCAGCTATACATCAAAAATTTTTTTGCATTTCATATTCTCTGCAGAGTTACTGTGAAGGAA
dolphin AAACAATAAGGTCCTACTGTATAGCACAGAGAACTATATTCATCATCTTGTAATAATCTATAACAGAAAAGAATCTAAATATATATATATGTGTGTGTGTGTGTGTATGTGTATGTATAACTGAATCACTTTGCTCTGTG-CTGAAACTGACACAACATTGTAAATCAACTATACTTCAAGAA--AATTTGCATTTCGTATACTCTACAGAGTGATTGTGAATGGA
alpaca  ------------------------------------------------------------------------------------------------------------------------------------------------------------------------------AATTTGCGTTTCATATTCCCAGCAGAGTTACTGTGAAGGCA
pig     ------------------------------------------------------------------------------------------------------------------------------------------------------------------------------AATTTGCATTTCATATTTTCTGCACAATTATTGTGAAGAGA
horse   ------------------------------------------------------------------------------------------------------------------------------------------------------------------------------AATTTGCATTTCTTTTTCTTGGCAGAGTTATTGTGAAGGAA
dog     ------------------------------------------------------------------------------------------------------------------------------------------------------------------------------GATTTGCATTTTATATTCTCTGTAGAATTCCTGTACAGGAA
bat     ------------------------------------------------------------------------------------------------------------------------------------------------------------------------------CGTTTGCATTTCACGTTCTCAGCGGAGTGAGTGTGAGGGGC

cow     GCTATTTTCT-TTCGCCTTTAAGC
dolphin GCCGTTTTCT-CTTACCTTTAAGC
alpaca  GCTATTTTCTCTTTATCTTTAAGC
pig     GCTAT------TTTATCTTTAAGC
horse   GCTATTTTCC-CTTACCTTTAAAA
dog     GCTGTTTCCCCTTAACCTTTAAGC
bat     GCTGTCTC---CCAGCCTGTAAGG
```

C4-1234 – L1-2_BT

```
cow     AACAGTGTTCATACTTAAGTTGGAGACATAAACTTCTATTGAAGTGTTATCTTTCAGTGTTT----TTCACTATATACTTAACTTTCTTTTTC-TGTTTTTTAAAATTTA--TTACTGAAGTATAGTTGATTTACAGTGTTGTGCTAGTTTCTGGTATACAGCAAAGTGATTGAGTTATACATATATATACATTCTTCTTCATACTTTTTTCCATTGTGGTTTATCAT
dolphin AATAGTGTTCATACGTAAGATGGAGACATGGGCTTCTGTTGAAGTGTTAGCTTTCAGTGTCT----TTCACTCTATACTTAACTTTCTTTTTCTTTTTTTTTTAAATTTATTTTATTGATGTATAGTTGATTGACAATGCTGCGTTCGTTTCTGATGTACAG-GAAGTGATTGAGTTATAC--AAATATACACTCTTTTTCATACTCTTTTCCATTATGGTTTATCCC
alpaca  GAAAGTGTTCATACATAGGATGGCGACATGGGCTTCTATTAAAATGCTGGCTTTCAGCGTTT----TTCACTCTATAGTTAAC-------------------------------------------------------------------------------------------------------------------------------------------------
pig     GATAGTGTTCATACATAAGATGGAGACATAAGCTTCTGTTGAAATGTTAGCTTTCAGCATTT----TTCACTCTATACTTAAC-------------------------------------------------------------------------------------------------------------------------------------------------
horse   AGTGGTGCTAATACATAAGATGGAGACAGGGGCTTCTGTTGGAATGTTGGCTTTTAGTGTTT----TTCACTCTAAAC------------------------------------------------------------------------------------------------------------------------------------------------------
dog     AGTACTGCTTATACATAAGATGGAGACATGGGCTTC---TGGAATGTTTGTTTTTAGCATATCAACTTCTCACTGTACTTAAT-------------------------------------------------------------------------------------------------------------------------------------------------
bat     AATAGTGCTTACACATAAAACAGAGAGATGGGCTCCTATTGGAATGCTAGCTTTTAGTGTTT----TTCAC--TATATTTAAC-------------------------------------------------------------------------------------------------------------------------------------------------

cow     GGGATATTAAATATAGCTCCCTGTGCTACACAGTA------TATTGTTTATCCATTCAATATATAATAGTTTGCATCTGCTAATCCCAAACTCCCAGTCCATACCTCCCTCA--------------CCCACTCCCTTGGCAACCACAAGTGTGTCT-------------GTCTGTTC---TTTCATAGATACGTTCATATGTGTCGTATTTTAGA-TTCCACATATAA
dolphin AGGATATTGAATATAGCTCCCTGTGCTATACAGTAGGACCTTGTTGTTTATCCATTCTATACATAATAGTTTGCATCTGCTAATCCCAACCCCCCAATCCATACCTCCCTTACCCACCCACCCACCCCCACCCCCCCGGCAACCACAAATCTGTTCTCTATGTCTCTGAGTCTGTTTCTGTCTCGTAGAGAAGTTCATTTGTGTCATATTTTAGATTTCCACATATAA
alpaca  ------------------------------------------------------------------------------------------------------------------------------------------------------------------------------------------------------------------------------------
pig     ------------------------------------------------------------------------------------------------------------------------------------------------------------------------------------------------------------------------------------
horse   ------------------------------------------------------------------------------------------------------------------------------------------------------------------------------------------------------------------------------------
dog     ------------------------------------------------------------------------------------------------------------------------------------------------------------------------------------------------------------------------------------
bat     ------------------------------------------------------------------------------------------------------------------------------------------------------------------------------------------------------------------------------------

cow     GTGATTGTACTTAACTTTTCTTTTTGCCTTCTTCTAGAATGGAGAGAGACCATCCCATGAAGTACAGATAAT
dolphin GTGATTATACTTAAC-TTTCTTTTTGCCGTCTTCTAGAACGGAGAGAGACCATCTCATGAAGTACAGATAAT
alpaca  ----------------TTTCTTTTTGCCTTCTTCTAGAATGGAGAGAGACCATCTCATGAAGTACAGATAAT
pig     ----------------TTTCTTTTTGCCTTCTTCTAGAATGGAGAAAGACCATCTCACGAAGTACAGATAAT
horse   ----------------TTTCTTCTTGCCGCTTTCTAGAATGGAGAGAGACCATCTCATGAAGTACAGATAAT
dog     ----------------TTTCTTTTTGCC---TTCTAGAATGGAGAACGACCATCTCATGAAGTACAGATAAT
bat     ----------------TTCCTTTTTGCC-TTTTCTAGAATGGAGAGAGGCCATCTCATGAAGTACAAGTAAT
```

C4-1341 – L1-2_BT

```
cow     AATACAAGGTGTTGCACGTAGTTGGCATATTTAAAAGGAGTTGACC----TGTTGTTGGAGAAGGTGTTT-------AATCTGTGGTAT--------ATTTTTTATTTTTTTTAATTTTTATTGGAAAATAGTTGGTTTACAGAGTTGTATTAGTTTCAGGTATTCAGCAAAGTGAATCAGTTATACATATAAGTATATCCACTCTTTTTTTAGATTCTTTTTTCACA
dolphin GATATAAAGTATTGCACGTAGTTGGCATATTTCAAGGGAG-TGACC----TGTTTTTGGAGAGAGTGTTT-------AATCTCTGGTATTTTTTTTTATTTTTTTTATTTTTTTTAAATTTTTATTGGAGTATAGTTGATTTACAATGTTGTGTTAGTTTCAGGTGTACAGCACAGTGAGTCAGTTATATATATACATATATCCAGTCTGTTTTTAGATTCTTTTCACCTA
alpaca  AATGTAAGGTGTTGCATGTTAGTGACGTATTTCAAAGGGAGTGACC----TGTTGTTGGAGAGGATGTTT-------AATCGCTGGTAT-------------------------------------------------------------------------------------------------------------------------------------------
pig     CATATAAGATATTGCACGTAGTTGGCATATTTCAAGGGAG-TGACC----TGTTTTTGGAGAGAGTGTTT-------AATC--TAGTAT-------------------------------------------------------------------------------------------------------------------------------------------
horse   GATAGAAAGTGTTGCATGTAGTTGGCATATTTCAAGGGGG-TGATG----TGTTGTTGGGGAGGGTGTTT-------AATCTCTGGTAT-------------------------------------------------------------------------------------------------------------------------------------------
dog     TAAAAATAGAGTTTCATGTAGTTGGCATTTTTGAAGGGGG-TGATGTGTTTGTTGTTGGGGAAGGTGTTT-------AATCTCTGAAAT-------------------------------------------------------------------------------------------------------------------------------------------
bat     AATATAAGATAGTGCATGTAGTTGCCATATTTCCAAGGGG--CATG----TGTTGTTGGAGAGGGTGTTTTTACTTGAATCTCTGGCAT-------------------------------------------------------------------------------------------------------------------------------------------

cow     TAGGGCATTACTGAGTATTGAGTAGAGTTCTTTGTGCTGTACAGTAGATTCTTACT-GTTGTCTGTTTTATATGTAGT--TGTGTATATGTCAATTCCAGTCTCCCAATTTGGTATATTTTTTTTAATGTATAGAGTAATAGGGGCATTTTATATCAGCATGAGTG--CCATTAGCA
dolphin TAGGCCATTACAGAGTATTGAGTAGAGTTCCCTGTGCTATACAGTATGTCCTTATTAGTTACCTATTTTATACATAATAGTGTGTATATGTCAATCCCATTCTCCCAATTTGGTAT-ATTTTTCAATGTTTAGAGTAATGGGTTCATTTCTTACCAGCAGGAGTG--CCATTGGCA
alpaca  ---------------------------------------------------------------------------------------------------------------------ATTTTTTAATGTATAGAGTAATGGGCTCATTTCTTACCAGCAGGAGTG--CCATTGGCA
pig     ---------------------------------------------------------------------------------------------------------------------ATTTTTTACTGTATAGAGTAATGAG-----TTCTTACCAGCAGAAGTA--CCATTGGCA
horse   ---------------------------------------------------------------------------------------------------------------------ATTTTTTAATGTATAGAGTAATGGGTTAATTTCTTACCAGCAGGAGTG--CCATTAGCA
dog     --------------------------------------------------------------------------------------------------------------------ATTTTTTTAATGTATAGAGTAATGGGTTTGTTTCTTGCCAGCAGAAAGGGCCATTTGGAA
bat     ---------------------------------------------------------------------------------------------------------------------ATTTTTTAAT--ATGGAGATATGGGTTTATTTTTTACCAGCAAGAGTG--CCATTGGTA
```

Cow-dolphin-pig monophyly

C4-644 - L1-2_BT

```
cow     TGTCAATAATATATCAATAAGGCTCGAAT----AAAAAC-----CCAAATAAAAGGTAAAACGCTGTATTCAGCATC--TTGTAATAATTATTAGTGGAAAAGAATATGAAAAAGAATACATATAT---------------AACTGAATCATGTTGCTGTTTACCAGAAACTAACACAACATTGTAAATCAACTATACTAGAATTTTTTGAAAA--------------
dolphin TGTCAATAATATATCAATAAAGCTGGAAT----AAAAAC-----CCAAATAAAAGGTAAAGCTCTGTATTCAACATC--TTGTAATTACCTATAAGGAAAAAGAAGTTTTTA---TATATATATAT---------------AACTGAA-----TTGCTGTATACCAGAAACTAACACAACATTGTAAACCAGCTATACTTAAAGTAAAAAAAAGCGGGGGGGGGGGGT
pig     TGTCAATAATATATTGGCAAAGCTGGAAT----AAAAAC-----CCAAATAAAAGGTAAAGCTCTATATTCACTATCTTTTGTAATAACCTATAATGGAGAGGAATACATATATGTGTATATATATATTCTAGAGAGAGAGAATTGAATCACTTTGCTGTATACCTGAAACTAACACAACATTGTAAATCAACTGTACTTCAGATTTTTTTTTTAA------------
alpaca  TGTCAATAATATATCAGTAAAGCTGGAAT----AAAAAC-----CCAAATAAA-----------------------------------------------------------------------------------------------------------------------------------------------------------------------------------------------
horse   GGTCAGTAATATCTCAATAAAGCTGGAAT----GAAAAC-----TCAAAAAA------------------------------------------------------------------------------------------------------------------------------------------------------------------------------------------------
dog     TGTCAATAATGTCCCAATAAATCTGGAAT----AAACCCTCCTCTCAAAAAA------------------------------------------------------------------------------------------------------------------------------------------------------------------------------------------------
bat     TGTTAATA------TCTCAAAGCTAGAAC----AAAGAC------CAAATAA------------------------------------------------------------------------------------------------------------------------------------------------------------------------------------------------
shrew   TTTTATT--TATGTCATTAAAACTAGAGTTTTTAAAAAC-------CACAAA------------------------------------------------------------------------------------------------------------------------------------------------------------------------------------------------
human   TCTAAAAAGTAACGGAGGAGAGGCGGAAC----AAAAGA-------AAAAAA------------------------------------------------------------------------------------------------------------------------------------------------------------------------------------------------

cow     GAGTAAAGCTAATAAATGTTGGACAGACTAGAAG-CAGTATCTCCAAGAAGAG---------------------GAACTGGG
dolphin GGGTAAAGCTAATAAATGTTGGACAGACTGGAAG-CAATAGCTCCAAGAGGAG---------------------GGACTGGG
pig     AGGTAAAGCTAATAAATATTGGACAGACTGAAG--TAACAGCTCCAGGAGG-----------------------GACTTGGG
alpaca  AGATAATGTCAGTAAGTGTTGGACAGATTGGAA--CTGTAGCTCTAAG--------------------------GGACTGGG
horse   AGGTAAAGCTAATAACTGTTGGACAGATTGGAAGCCAGTAGCTCCAAGAGGAAGAACATGTAGTCCCAGAGTAGAGACTGGA
dog     AGATAAAGCTAATAGATGTTGGATAGATTAACAG-CTGTAGCTCCAAGAGGAGGGACATGTAGTGCCAATGTAGATACTGGG
bat     AGGTAAATCTAATAAATGTTGGACAGATTGGAAG-CAGTGGCTCCAAGGGGAGGGACATGTAGTGCCAATATAAGGACTGGG
shrew   AGGTAAAGCCAATGAATGTTAGATAAGAAGTAAA------------AGAGGGA----------------TATATAAACTGGG
human   TAGTATAGCTGAAAACTGTTGGACAGATTAGACG-TAATAGCTCTAAGAAGAGGGACGTGTAGGGCCAATATAGAGATTGGG
```

C4-1343 - L1-2_BT

```
cow     -TCTTGCTG----CTAAGTAGCTTTATTGAATCTAATCAGTAATTGTATAG--AGAGGTCAGTGTGGGCACTGGGATTCTTTTTTATTTTCTCCTT---------------CAATTTTGATTTAGTTGATTTACAATTTTGCATTAGTTTCA-GATGTATAGCACAGTGATTTGGTTATACATATATACATA---AT----------------TGTTTTTCAGATGCT
dolphin -TCTTGCTG----ATAAGTAGCTTCATTGACTCTGGTCAATAACTGTATAG--AGAGGTCATTGTAGGCACTAGGATT--CCCCAACCCCCCCCCC---------------AAATTTTGACTTAGTTGATTTACAATTTTCGGTTAGTTTCAGGGGGTACAGCAAAGTGATTTGGTTATACGTATGTATGTATATGT----------------TCTTTTTCAGATTCT
pig     -TGTTGCTG----CTAAGTAGCTTCAGTGACTTTGGGCAGTAACTGTATAGATAGAGGCCAGTGTAGGTGCTGGGATTCTTTTTTAAAAAAATTTTTTTG/.../AAACAAAAAAAATGACATGGTTGACTCAGAATCTTAGATTAATTTCA-GGTGTACAGCAAAATGATTTGGTTATACTTACATGTATACCTATATGTGTGTGAGTGTATGTTTTTCAAATTATT
alpaca  -TTTTGCTG----CTAATTAGC-TCATTGACTCTGATCAGTAACTATGTAG--AGGGATCAATATAGGTGCTAGAATT------------------------------------------------------------------------------------------------------------------------------------------------------
horse   CTTTTCCTGCTGTCTTAGTACCTTCATTGACTCTGGACCGTAACTGTAGAG--AGAGACCAGCATAGGTGCTAAGATT------------------------------------------------------------------------------------------------------------------------------------------------------
dog     --TTTCCTG----CTTAGTAGCTTCCTTGA--CTGGTCAGTAAC--TAGGG--AGATGCCAGTGTAGGTGCTAGGATT------------------------------------------------------------------------------------------------------------------------------------------------------
bat     -TTTTGCTGGAATCTTAATAGCTTCATTGA--CTGGTCAGTAACTGTATAG--CAAGGCCAGCATAAGTGCTGGGATT------------------------------------------------------------------------------------------------------------------------------------------------------

cow     TTTCCTTTTGGGTTATTGTAAGACATTGAATGTAATTCCCTATGCTATATAGTAAATCCTTTT----AATATGTTTTACTTGTAGTAGTGCATATCTGTTAATCCCATATTCTTAATTTATCCCTCCTCCTCTTTCCCTTTTGGTAACCATAATTTTGTTTTTTGTGTC--TGAGTCTGTTTCTGTTTTGTGAGTTGGGTTTATTTATATTATGGTGCTGTGATTCTT
dolphin TTTTCTTTTAGGTTATTGTAAGATATTGAATATAATTTATTGTGCTATGTAGTAAATTCTTTT----CATCTATTTTACATGTAGTAGTGTGTATCTGTCATTCCCATACGCCTAATTTATCCCTTCTCCCCTTTCCCTTTGGGTAACCATAATTTTGTTTTCTGTGTCTATACGTCTGTTTCTGTTTTGTAAA-TAAGTTCATTTGTATTATGGTGCTAGGATTCTT
pig     TTGCATTATTTTGCAATATAAGATGTTGAGTATAGTTCCCTGTGCTTTGCAGTAAACCCTTTTTGCTAATCTATTTTTATGTATAGTAGTGTGTATCTGTTAATCCCATACTCCTACTTTATCTCTCC-CCCCTTTCCTTTTTGGTAATTATAATTTTGTTCTCTATGTCTATGAGTTTGTTT--GGTTTGATAAGTAAGTTTGTTTGCCTTATGGTG--------CTT
alpaca  ---------------------------------------------------------------------------------------------------------------------------------------------------------------------------------------------------------------------------------CTT
horse   ---------------------------------------------------------------------------------------------------------------------------------------------------------------------------------------------------------------------------------CTT
dog     ---------------------------------------------------------------------------------------------------------------------------------------------------------------------------------------------------------------------------------CTT
bat     ---------------------------------------------------------------------------------------------------------------------------------------------------------------------------------------------------------------------------------CTT

cow     AGGAAGGGACACTGGGACAGAATGTTCACAG---TTTATATGTGA-GAACCAGAGC---TCCAAAGTTTGTATTTCTTAACCAT-------CTTTCATCATTTAAAAAATTCCTTTGCCCCGAATTTCTTC---
dolphin AGGAAGGGCCATTAGGACAGAATCGTCAGGG--TTTTATACCTGA-GTTCCAGAAC---CCCAAAGTTTGTATTTCCTTACCAT-------CTCTCATC-TTTTTAAAATTCCTCTGCCCCAGATCTCTCC---
pig     AGGAAGGGCCATTAGGGCAGAATGGTCAGGG----ATTTATATGAGGAACCAAGAC---CACAAAATTTGTATTTCCTTATCAT------CTCTTTATCTTTTTAAAAATTCCACTGCCCAAAATCT-------
alpaca  AGGAAGGGCCATTAGGACAAAATGGTCATGGTAATTAATATGTGAGGAACCAGAAC---CCCAAAGTTACTAATTCCTTACCAC-------CTCTCATCTTTTTAAAAATTCCTCTGTCCTAAATATTTCT---
horse   AGAAAGGGCCATTAGGATAGAGTGGTCAGGG----TTAGACCTGGGGAACCAGAAC---TCCAAGGTTTATGTTTCCTTATCTCTCATTCTTTTTTTTTTTTTTTTTAAATTTCTCTGTTTAAAATCTTTCT---
dog     AGGAGGGGATCAT----TAGAGTGATCAGGG----TTATACCTGAGGGACTAGAATAGAACCGCAGTTTGCATTTCTTTACCACCTCTAATTTTTCTTTCTTTTTAAA----TTCTCTTTAAAATCTTTTTTTT
bat     ----AGGGCCATTAGGACAGACTGGTCAGGG----TTAGGCGTGAGGAGCAAGAAC---CTTAAATTGTATATCTCCTTGACTT---CTCTCGTTCTTATTTATAAAAATTCATCTGTTTAAAATCTTTCT---
```

Cetartiodactyla monophyly

C4-93 - L1-2_BT

```
cow     TTGGCTGCATAATTTTCCTATAGATATTACCT-AAAGTCCATATTATATCTTTTTAAAAAAATTAGTTACTT---------------ACTCATTTATTTTTATTAAGGTATAGTTGATGTACAATATTATTTAAGTTTCAGGTGGACAACATAGTGATTCTCAACTTTTCAAAATTATTACTCTGTTTATCATTATTGCATAATATGGGCTATATTCCCTGCACTATG
pig     TTGGTTGCAGAATTTTCCCGGAGACATTGTCT-AACATTCATGTTATTAATGAATTAATAGTTTATATTGCTCAATTAATTAGTTCAGTTAATTAGTTTATATTGAGGTATAGTTGATGCACAATACTATTTAAGGTTTAGGTGTACAATGTAGTGATTTTCAATATTTAAATATTG-TATTCCATTTATAGTTTTTATAATATATTGGTTATAGTCCCTGTCCTGTA
alpaca  TTGGCTGCAGAATTTTCCTGGAAATGTTGTCT-AATATCCATATTATTTCTTTTAAAAG------------------------------TTGTTTATTTTTATTGAGGTATAGTTAATGTACAATATTATTTAAGTTTCAGGTGTATGACATAGTGATTCACAATTTTTAAAGATTA-TATTCTATTTGTAATTATTGTAAAATATTAACTATAGTTCCTGTGCTGTA
horse   TTGGTTGCAGGATTTTCCTGGAGATGTTATCT-AAAACCCATATTATTTC----------------------------------------------------------------------------------------------------------------------------------------------------------------------------------
dog     TTGGTTGCAGAACTCTCCCGGAGACATGATCT-AAATCCTACTTTATTTC----------------------------------------------------------------------------------------------------------------------------------------------------------------------------------
cat     TTGGTTGCAGAATTCTC--AGAGATAGGATCT-AAAACCCACCTTATGCC----------------------------------------------------------------------------------------------------------------------------------------------------------------------------------
panda   TTGGTTGCAGAATTCTCTTGGAGATGTGCTCT-AAACCCCACATGACGTC----------------------------------------------------------------------------------------------------------------------------------------------------------------------------------
megabat TTGGTTGCAGAATTTTCCTGGAGATGTTGTCT-AAAATCTATATTATTTC----------------------------------------------------------------------------------------------------------------------------------------------------------------------------------
microbatTTGGCTGCAGAATTTTCCTGGAGATGTTGTCC-AAAATCCATATTATGTTTT--------------------------------------------------------------------------------------------------------------------------------------------------------------------------------
human   TGGGTTGCAGAACTTTCCCAGGGATGTTATCTAAAAAGCCATATTATTTC----------------------------------------------------------------------------------------------------------------------------------------------------------------------------------

cow     CAATATATCCTTGTAGCTTATTTATTTTATACATAGCAGTTTGTTCCTCTTAATCCCGTACCCTATGGTATCTTCCCCTACCCGCTCCCCTCTTCCCACTGGTCATCACTGGTTTGTTCTCTGTGAGTCTGCTTCTTAT----------TTGTTATATTCACTAGCTTGTTTTCTTTTTTAGATTCCACATAGAAGG----GTCATATAGTATAACATCTATGTTGTT
pig     TGACATATCCCTGTAGC----TTATTTTATACATAGTAGTTTGTACCTTGTAATCCCTGTCCCC----TATCTTGCCCCTCCCCCTCCC-TGTCCCTACTGATCACCACTGGTTTGTTCTCTATATCTGTGAGTCTTATTTCATTTTTGTTGCAGTCAGCCTAGGTTCTTTTTGTTCTTTAGATTTTCAAGAATAAGTAGTATCATACAGCATAAAATCTATACTGTT
alpaca  CAATACATCCTTGTGGCTTATTTATTTTATACATAGTACTTTGTACCTCTTAATCCCCTACCCC----TGTCTTGTCCCCCCCTCTCCCCTCCCCACATTGGTCCCCACTAGTCTGTTCTCTGTATCTGTGAGTCTGTTTCTTTT----TTGTTATTTTCACTAGTCTGTTGTACTTTTTAGATTCCACATA-TAAGTTATATTATACAGTATAAAAGCCATATTATT
horse   ------------------------------------------------------------------------------------------------------------------------------------------------------------------------------------------------------------------------------------
dog     ------------------------------------------------------------------------------------------------------------------------------------------------------------------------------------------------------------------------------------
cat     ------------------------------------------------------------------------------------------------------------------------------------------------------------------------------------------------------------------------------------
panda   ------------------------------------------------------------------------------------------------------------------------------------------------------------------------------------------------------------------------------------
megabat ------------------------------------------------------------------------------------------------------------------------------------------------------------------------------------------------------------------------------------
microbat------------------------------------------------------------------------------------------------------------------------------------------------------------------------------------------------------------------------------------
human   ------------------------------------------------------------------------------------------------------------------------------------------------------------------------------------------------------------------------------------

cow     AATTGATTTGTTTTAGGCAG--------AACAAAAAGCAGTATTAGTGTCCGTGGTCTGACTCATTTTG
pig     GATTGATGTGTTTTAGGCAG--------AACAAAAAGCAGTGCTAGTGCCCATGGCCTGGCTCATTTCA
alpaca  CCTCAATTTGTTTTACGTAG-------GGAAAAAAAGCCATCGTAGTGTCCGTGGTCTGGATCACTTGG
horse   --CTGGTTTGTTTTAGATAG----------AAAAAAGCAATATTAACGTTTA-GGTCTGACTCATGTCA
dog     --TTGATTTGTTCTAGGTAGAAAAAAAAAAAAAAAAGCAATATTAGTGCCCATGGTCTGGCTCATTTCA
cat     --TTGATTTGTTTTAGGTAG--------GAAAAAAAGCAATATTAGTGTCCATGGTCTGGCTCATTTCA
panda   --TTGATTTGTTTTAGGTAG------AAAAAAAAAAGCAATATTAGTGTCCATGGTCTGGCTCATTTCC
megabat --TGGATTTGCCTTAGGTAG-------AAAAAAATAGCAATCTTAGTGTTCATGGTCTGGCTCATTTCA
microbat--ATAATTTGTTTGAGGTAG-------AAAAAAACAACCATCTAAGTGTCCATGGTCTGGCTCATTTCA
human   --TTGATGTAATGTAG-------------AAAAAAAGCAGTATTGGTGTCCATGACCTGGCTCATTTCA
```

Conflicting retroposon insertions

#6017 (carnivora+chiroptera+perissodactyla) – (cetartiodactyla)

```
dog     GAATAATTGTTTGAAA-------TTTTTACTTAT------AACAATTTTTCACTACATGAAAATAACACTGGAGCCACCTACA------AATGTGTTTGTAGGTTTATATCTTGATCTAACAT---GTTACCTTAGTGCT---GTTAACTATTTAATGACAATTTTAAAAAATTTTATGTATGGCA---TAGGTG-----------TTT-ATTGAGGTATAATTGATA
horse   GAATAATCCTTTTAAA-------TTTTTATTTGTACTTATAATAATTTTTCACCATGTGGAAGTCATAGTGGAACCACCTACA-----GCATGTGTTTGTATGTTTATATCTTGACCTAACATTATGTTACCTTAGTTTTCTGTTAGGGTGTTTA-TGACAATTTT-CAAAATTTTGTGTATGACA---TATATATAA-CTATTTTTTTAATTGAGGTATAATTGACA
megabat CAATAATCTTTTAGAA-------CTTTTATTCTTGT-----AACTTTTACCATTAGATGGAAATAATAGTGGAGCTACCTACATATAAGCATTTGTTTATATGTTTATGTCCTGTCCTAA-------------------------------ATTT-TGAGAGCTTT-CAAAATTTTGTGTATGATG---TATGCGTAAGTTTTTCTTTTAATTGAGTTATAATTGATG
alpaca  AAATTATCCTTTTAAATAATTATTTGTTATTTACTCTTATAATAATTTTTTACCACATGGAAATAACAATGGAACCATATACA---------ATGTGTTTATGTTTATATCTTGACCTAACATTATGTTACCTT-GTTTTCTGTGAAGGTATTTAGTGACAGTTTT-CAAACTTTTGTGTATGATA---TACATGTAA------------------------------
cow     GAATGATACTTCTAA--------TTTTTATTTATTTTTAT----ACTCTTTTATATGTGAA-----------AGCCACCTATA-----GTATTTGTTTATATGTTTACAT-TTGACCTAACATTATGTT-CATTTACTATCTATTAAAGTGTTGACTGATAATTTT-CAAACTTTTATATATGATA-------TTAAA------------------------------
human   GAATTACCCTTTTACA-------TTTTTATTTATTTTTA-AATAACTTTTTATCATGTGG------------AGCCACCTATA-------GCATGTATTTATGTTTATAATTTGACCTAACATTATTTTACCTTGATTTTCTGTTAAGCTGTTAGGTGACAATTTT-CAAAATTTTGCATATGATGATTTGTGTATTA------------------------------

dog     TATA------CATTAGTTTCTGATGTATGAGGTAATAATTTGATATTTGTATACATTGTGAAATGATCACCACAGTAAGTCAGATTATAATCACATCCATCACCATACAGTGTTAAAAAAATTTTTTTTTCTTTTGAGAACTTCTAAGATTTATTTACTCTCTTAGCAACTTTAAAATAATGCACTAGAATATTATTAACTATAGTCTGTCGGTTT-----TAATTCA
horse   TATAAGATTCTATTAGTTTCAGGTGTGCAACATAATGATTCAATATTTGCATATGCTGCAAAATGATCACCACAGTAATTCTAGTTACCA-----TCCATCACCATACAGTGTTAC-GAATTTTTTTCTTATAATGAGAACTTTTAAG----ATTTACTCTCTTAGCAACTTTCAGAT-ATGCAATATGGTATTGTTAACTCGAGTATAT--GTTTTAAATTAATTTA
megabat TATAACATTATATTTGTTTCAGGCATACAGCATAATGACTCAATATTTGAATACACTGAGAAATGATCATCACAATAAGTTTAGATACCA--ACTGTCACAGTATTATAGTATTGCAAAGTTTTTTTCTTGTGATGAAAACTTTTAAG----ATTTACTCTCTTAGCAACTTTCAAAT-ATGAACTATAGTACTATTAACTATAGTATATAAGTTTTAAGTTAATTTA
alpaca  ----------------------------------------------------------------------------------------------------------------------------------------------------------------------------------------------------------------------G TTTTAAATTAATTTA
cow     ----------------------------------------------------------------------------------------------------------------------------------------------------------------------------------------------------------------------G TTTCAAATAATTTTA
human   ----------------------------------------------------------------------------------------------------------------------------------------------------------------------------------------------------------------------G TTTTAAAGTAATTTA

dog     TAAA-----CTTGATTTAAGAACCGAAGACTGAATGGAATTAAAT---------------ATGTTAACAGTTTCTGAAT----T
horse   TAAAGTAACTTTGATTTAAGAATTGAAGATTGAATGGAAGTAAAT----GTAAGTTCTTAACACAAAGTTTCTATGAGT----T
megabat TAAAGTAATCTTGATTTAAGATTTAAAGATTGAATGGAAGTAAAT----GTAAGTTCTTAACACAAAGTCTCTATGAGTTACAT
alpaca  TAAAGTACCCCTGATTTAAAAATTAAGGATTGAATGGAACTAA--------AAGTTCTTAACATAAAATTTCTA--AGTTACTT
cow     TAAAATAACCTTGATTTAAAAACTGAAGATTGAACAGAACTAAAA----GTAAGTTCTTAACATAAAATTTCTATGAGT----T
human   TAAAGTAGCC-----TTAAGAACTGAAGACTGAATGGTATCAAATCTAAGTTAGTTTTTAGTGCAAAATTCCTATAAGCTAATT
```

#7214 (carnivora+cetartiodactyla+chriroptera) - (perissodactyla)

```
panda   AAAACAAGAATTGTTC-CTGTCTTCTGTTGAGGTTATAAGA-TACTAATAT-CCTGGTTTATTT-----------TTAAACCTTTTTTAATGAAGTATGAT---------------TATTAGTTTTGGGCGTACA---ACATAATGATTTGATATTTGTGTCCATTGCAAAATGATCACTGTCCTGGTTGATTTTGGGTTTTTTTGGGTTTTTTTTGTTTTTTTTTTT
cat     AGAATAAGACCTGTTC-CTCTCTTCTGTTGAGGTTATGAAACTACTCATGT-CCTGGTTAATTT-----------CAAATTCTTTTAAATCGAGGCATAATTGACCTACAGCATTATATTCGCTTGAGGTGTGCA---ACATAACGATTCGATGTTTGTGTACGTTGCAAAACGATCACAG-----------------------------------------------
dog     AAAACAAGAATT-----CTCTCTTCTGTTGAGGTTATGATG-TACTAATGT-TCTGGTTTATTTT----------TAAGCCTTTTTTAATTGAGGCATAGTTGACATATAACATTATATTAGTTTCAGGTGTACA---GCATAATGATTTGGGATTTGTATACATTGCAAAATGATC---------------------------------------------------
cow     GAAACAAAAATTGTTCCTTCTCTTCTGTTGAGGTTAG-ACA-TACTAATGT-TGTGGTTTGTTCAGTTATTAAAGTAA---CTTTTTAATTGAGGTGTAATTGAC----------ATATTGCTTTTGAGTGTACA---GCATAATGATGCAATATTTGTACGCATTGTGAAATGATC---------------------------------------------------
dolphin AAAACAAGAATTGATCCTTCTCTTCTGGTGCGGTTAG-AGG-TACTAATGT-CCTGGTTTATTTATTTATTAAAAGAAATTTTTTTAAACTGAGGTATAATTGACATAAA--GTTATATTGGTTTTGGGTGTACA---ACATAATGATGCGATATTTGTATACATTGTGAAATGATC---------------------------------------------------
alpaca  AAGAC-----TTTTCCTTCTTCTTCTGTTGAAGGTAGGGGG-TACTAATGTCCCTGGTTTATTTATTTATTAAAAGAAG-TTTTTTAAATTGAGGTATAATGGACATACAAGATTATATTTGTTTCCTGTATACA---GTATAATGATGCAATATTTGTATACATTGTGAAATGATC---------------------------------------------------
megabat AGAACAAGACGTGTTC-----CTTCTGGTAGGGTGT--TAG-CTGCAATGT-CCTGGTTTATTTATTTA-----------ATTTTTAAATTGAGGTATAATTGAAATATA-----ACATTAGTTTTAGGTATATATTTAGGCAATGATTCAGTATTTGTTTACATTGTGAAATTATC---------------------------------------------------
horse   AAAGCAAGAATTGTTC----TTTTCTGTTGAGGTTGT-TGC-TGCTAATGT-----------------------------------------------------------------------------------------------------------------------------------------------------------------------------------------------------------------------------
shrew   AAAACAAAAATCCCGCCCACTCTTCTTCTGAGGTTAC-AGG-CTCT----------------------------------------------------------------------------------------------------------------------------------------------------------------------------------------------------------------------------------
hedgehogAAAACGAGAACCATTTCCTACCTTTTG--GGGCTTAT-AGG-TGCT----------------------------------------------------------------------------------------------------------------------------------------------------------------------------------------------------------------------------------
human   AAAAAAAAAAAGATTCCTTGTTTTCTGTTGAGGTTAT-AGA-TACC----------------------------------------------------------------------------------------------------------------------------------------------------------------------------------------------------------------------------------

panda   TTTTTAAAGATTTTATTTATTTATTTAACAGAGATAGACACAGCCAGCGAGAGAGGGAACACAAGCAGGGGGAGTGGGAGAGGAAGAAGCAGGCTCACAGCAGAAGAGCCTGACGTGGGGCTCGATCCCATAACGCCGGGATCACGCCCTGAGCCGAAGGCAGACGCTTAACCGCTGTGCCACCCAGGCGCCCCTGTCCTGGTTGATTTTGATCCGGTTTATTTCGTC
cat     ------------------------------------------------------------------------------------------------------------------------------------------------------------------------------------------------ACTGCCTTGGATTATTTTGATCCAGTTTATTTGATA
dog     ------------------------------------------------------------------------------------------------------------------------------------------------------------------------------------------------ACTGTCCTCGTTTATTCTGATCTAGTTTATTTTATA
cow     ------------------------------------------------------------------------------------------------------------------------------------------------------------------------------------------------ACTGTTTTGG----TTTTAACTCAATTTATTTTACA
dolphin ------------------------------------------------------------------------------------------------------------------------------------------------------------------------------------------------ACTGTTCTGG-----TTTAATTCAATTTATTTTACA
alpaca  ------------------------------------------------------------------------------------------------------------------------------------------------------------------------------------------------ACTGTTCTGGTTTATTT--ATCCAATTTATTTTCTA
megabat ------------------------------------------------------------------------------------------------------------------------------------------------------------------------------------------------ACTATCCTGGTTTATTTTAATCTAATTTTTTTTATA
horse   -------------------------------------------------------------------------------------------------------------------------------------------------------------------------------------------------------CTTGGTTTATTTTAATCCAATTTATTTTA--
shrew   ------------------------------------------------------------------------------------------------------------------------------------------------------------------------------------------------ACTATCCTGGTTTATTTTAACCCGGTATAGGTGATA
hedgehog------------------------------------------------------------------------------------------------------------------------------------------------------------------------------------------------AATATCCTAATTCATTTTAATCCAATTTGTTTTTAT
human   ------------------------------------------------------------------------------------------------------------------------------------------------------------------------------------------------AATTTCCTAGTTTATTTTAATCCAGTGTATTTTGTA

panda   ACTTCAGCAGTTGGAAATTAGGATGAAACATGTAAA-TGT
cat     ACTTCAGCAGTGTGGAATTGGGATGAAATGTGTAAG-TGT
dog     ACTTCAGCAGTTTGGAATTAGGATGAAACATGTAAATTGT
cow     GCTTCAGCAGCTTGGAATTAGAATAAAAGATGTAAA-TGT
dolphin GCTTCAGCAGTTTGGAATTAGGATGAAAGATGTAAA-TGT
alpaca  CCTTCAGCAGTTTGGAATTAGAGTGAAAGATGTAAA-TGT
megabat GCTTCAGCAGTTTAAGGTTAGGATGAAAAATGGAAA-TAT
horse   ------------------TAGGATGAAAGGTGTAAA-TGA
shrew   GTTTCAGC--TTGGGGGTTGGGATGGAAGATATGCA-TGG
hedgehogACTTCAGCATTTTGGGATTAAGATAAGAGATGTACA-TGT
human   GCTTCAGCAGTTTGGAATTAGGATACAAGGTATAAA-TGT
```

#8382 (cetartiodactyla+perissodactyla+chiroptera) - (carnivora)

```
cow      CTAAAAGCCATCTATGTTGAGTAAGTT-TCTATTGA--CTATTTAATTGAAGTAATTTTTTTTT-ATCAGTTCAGGTATACAACACAGTGATTCAGTGTATGTCTATATTGTGAAATGATCACAGTGG---ATACAAT------------------TAACATGCATC-------------------------------------------------------------
alpaca   CTAAAGGCCATTTACGTTGAGTAAGTT-TCTATTAATGCTGTTTAATTGGAGTAAGCTTTTTAT--CCATTTCAGATGTACCACATTATGACTCAGTATACGTCTACATTGTGAAATGATCACAATTA---GTAAAGT------------------TAACGTGTATC-------------------------------------------------------------
pig      CTAAATGCCATCTATGTTGAGTAAGTT-TCTATTGACTGCATTTAATTGAAGTAATTTTTTTTAA-TCAGTTTCGGGTGTACGACATAATGATTCAGTGTATA--TGTATTGTGAAATGATCCCAATGA---GTACAGC------------------TAACATGCATCCCACACGTAATAATATTTTTTTTTCTTTCTTTTTCTTTTTTTAGGGCTACTCCTGTGGCAT
horse    CTAAAGGCCATCTATGTTGAGTAAGTT-TCTATTAACTCTCTTTAACTGAGGTAATTTTTTTTTA-TTAGTTTCAAATGTACAACATAATGATTCAATGTATGTATATATTTTGAAATGATCGCCACAATAAGTCTGGC------------------TAACCTGTATC-------------------------------------------------------------
microbatCTAAAGGCCATCTACGTTGAGTAAGTT-TCTATTGA--TTATTGAATTGAAGTAATATAGTTTGATTAGTTTCATGTGTACAATGTAATGATTCAATATGTGTATATATTGGGACATGGTTGCCACAAT--AAGTCGT------------------TAACATACATT-------------------------------------------------------------
megabat  CTAAAGGCCATCTATGTTGAGTAAGTT-TCTATTGACTCTACATAACTGAAATAATATTTTTTA-TTAGTTTCAGGTGTACAGCACAGTGATTCGGTATATGTATATATTATGACATGATTGCTATAA---GTCTATTGCATGCATCAGTCTATTACTACATGCATC------------------------------------------------------------
dog      CTAAAGGCCATTTATGTTGAGTACGTT-TCTATTAACTCTGTTTAATTGAAATAATACTTTTTA---------------------------------------------------------------------------------------------------------------------------------------------------------------------
panda    CTGAAGGCCATCTATGTTGAGTAAGTC-TCTATTAACTCTGTTCAATTGAAGTAACAGTTTTCG---------------------------------------------------------------------------------------------------------------------------------------------------------------------
shrew    CTAAAGGCCATCTATGTGGAGTAAGTTCCCTACTGACTGTGCTTAATCAAAATAACCCGTATTT-TTGGATCCA-----------------------------------------------------------------------------------------------------------------------------------------------------------
hedgehogCTAAAGGCCATCTATGTTGAGTAAGTT-TCTGTTAACTCTGGTTAATTATAATGACACAATATT---------------------------------------------------------------------------------------------------------------------------------------------------------------------
human    CTAAAGGCCATTTATGTGGAGTAAGTT-GTTATTGACTCTGTTGGATTGAAATAATATTTT------------------------------------------------------------------------------------------------------------------------------------------------------------------------
```

```
cow      -------------------------------------------------------------------------------------------------------------------------------------------------------------------------------------------------------------------CCCACACAGGAATATTTTT
alpaca   -------------------------------------------------------------------------------------------------------------------------------------------------------------------------------------------------------------------ATCACACATAGTAATATTTTA
pig      ATGGAAGTTCCCAGGCTAAGGGTCGAATAGGATCCATAGCCGCTAGCCTAAGCCACAGCCACAGCAGCACGGAATTCGAGCCACATCTTTGACCTCCGCTACAGCTCATGGCAATGCCAGATCCTTAACCCACTGAGCAAAGCCAGGGATCAAACCCAACATCTCATGGATCCTAGTCGGGTTTGTTAACCCTTGAGCTGCAAAGGGAACTCCCATAATAATCCTTTT
horse    --------------------------------------------------------------------------------------------------------------------------------------------------------------------------------------------------------------------ACCGACATAGGGCTCTTTTT
microbat-------------------------------------------------------------------------------------------------------------------------------------------------------------------------------------------------------------------ACCACATGTGGCAATGTATTT
megabat  -------------------------------------------------------------------------------------------------------------------------------------------------------------------------------------------------------------------ACCACACGTAGTAATATTTTT
dog      ------------------------------------------------------------------------------------------------------------------------------------------------------------------------------------------------------------------------------------
panda    ------------------------------------------------------------------------------------------------------------------------------------------------------------------------------------------------------------------------------------
shrew    ------------------------------------------------------------------------------------------------------------------------------------------------------------------------------------------------------------------------TTTT
hedgehog----------------------------------------------------------------------------------------------------------------------------------------------------------------------------------------------------------------------------------TT
human    ----------------------------------------------------------------------------------------------------------------------------------------------------------------------------------------------------------------------------------G
```

```
cow      TAATGTTTTACTCTCTTCT----------TGTGCACCCGATAC-TCATATTCTTTCTGATGCTCTTGCTTTAGTT
alpaca   TAATGCTTTACTCTGTTCT----------TGTGCATGGGACAC-TAATGTTCTTTCTGATGCTCTTTCTTTAGTT
pig      AAATGTTTTACTCTGTTCT----------GATGCATGAGA--C-TAATATTCTTTCTGATACTCTCATTTTAGCT
horse    AAATGTTTTA---TGTTCT----------TTTGCATGAAACAG-TAATATTCTTTTGAATGCTCTTACTTTAGCT
microbatTAA--GTGTATTATGTTCT----------TGCGTATGAGATGC-TAATGTTCTTTCCAAAGCTCTGACTTTAGTT
megabat  AAATGTATTA---TGTACT----------TGTGCACAAGATAC-TAATATTCTTTCCAATGCTCTTACTTTAGTT
dog      -AAAGTTTTATTATGTTTT----------TATGTGT--GACAC-TAATA----TTCTAACCCTCTTACTTTGGGT
panda    --ATGTTTTATTATGTTTT----------TATGTGTCAGACACTTAATATTCTTTCTAATGCTC------TGGTT
shrew    TAACGGTTTATTATGCTCT----------TGTGTGTGTGATAC-TGATAGTCTCTCTAATGTCCTCACTTCAGTT
hedgehogGAATGTTTTTTTTTCTTTCTTTTTTTAATGTGTACTTGGGATAC-TAATCTTCTCTCAAGCATCCTTTCTTTGGTT
human    AAATGTTCTATTATATACT----------TATGCATGAGATAC-TAATATCCTTTCTAATGCTCTTATTTTGGTG
```

#41443 *(cetartiodactyla+carnivora+chiroptera) - (perissodactyla)*

```
cow      GAAAGAACAAGCCTAATATG-AATTATGACAAACTCAG-CCGAGCCCTCAGATACTACTATGT-GAAGGTAATACACGTCC--------------------------ACCTCGAAA-CAAAAATTGTTCCTTC-----------TCTTCTGTTGAGGTTAG--ACATACTAATGT-TGTGGTTTGTTCAGTTATTAAAGTAA---CTTTTTAATTGAGGTGTAATTGA
alpaca   GAAAGAACAAGCCTAATATGAAATTATGACAAACTCAGCCCGCGCCCTCAGATACTATTATGTAAAAGGAAATACGCTCCC--------------------------ACTCCAAGA------CTTTTCCTTCT-----------TCTTCTGTTGAAGGTAGG-GGGTACTAATGTCCCTGGTTTATTTATTTATTAAAAGAAG-TTTTTTAAATTGAGGTATAATGGA
dolphin  GAAAGAACAAGCCTAATATG-AATTATGACAAACTCAG-CCGAGCCCTCAGATACTATTATGT-GAAGGTAATACACATCC--------------------------ACCTCAAAA-CAAGAATTGATCCTTC-----------TCTTCTGGTGCGGTTAG--AGGTACTAATGT-CCTGGTTTATTTATTTATTAAAAGAAATTTTTTTAAACTGAGGTATAATTGA
cat      GTAAGAACAAGCCTAACATG-AATTACGACAAACTCAG-CCGAGCCCTCCGATACTATTATGT-AAAGGTAATACGCATCC--------------------------ACCTCAGAA-TAAGACCTGTTC-CTC-----------TCTTCTGTTGAGGTTATGAAACTACTCATGT-CCTGGTTAATTT-----------CAAATTCTTTTAAATCGAGGCATAATTGA
dog      GGAAGAACAAGCCTAATATG-AATTACGACAAACTCAG-CCGAGCCCTCAGATACTATTATGT-AAAGGTAATTCACATCC--------------------------ACCTCAAAA-CAAGAATT-----CTC-----------TCTTCTGTTGAGGTTATG-ATGTACTAATGT-TCTGGTTTATTTT----------TAAGCCTTTTTTAATTGAGGCATAGTTGA
megabat  GAAAGAACAAGCCTAATATG-AACTACGACAAACTCAG-CCGAGCACTCAGATACTATTATGT-AAAGGTAACACACATCC--------------------------ACTTCAGAA-CAAGACGTGTT----------------CCTTCTGGTAGGGTGT---TAGCTGCAATGT-CCTGGTTTATTTATTTA-----------ATTTTTAAATTGAGGTATAATTGA
horse    GAAAGAACAAGCCTAATATG-AACTATGACAAACTCAG-CCGAGCCCTCAGATACTACTACGT-AAAGGTAGTACACATCC--------------------------TCCTCAAAG-CAAGAATTGTTC---------------TTTTCTGTTGAGGTTGT--TGCTGCTAATGT-CTTGGTTTATT----------------------------------------
hedgehogGAAAGAATAAGCCAAACATG-AATTACGATAAGCTCAG-TCGAGCCCTCAGATACTATTATGT-AAAGGTAATGTGCATCTTGATGTTA----ACCCTGCACAGAAAAGCTCAAAA-CGAGAACCATTTCCTA-----------CCTTTTGG--GGCTTAT--AGGTGCTAATAT-CCTAATTCATT----------------------------------------
shrew    GGAAGAACAAGCCCAACATG-AACTACGATAAGCTCAG-CCGGGCCCTCCGATACTACTATGT-GAAGGTAACGCGTGTCCTTAAGTGACGGCTGCCTATATAGAGAACTTCAAAAACAAAAATCCCGCCCAC-----------TCTTCTTCTGAGGTTAC--AGGCTCTACTAT-CCTGGTTTATT----------------------------------------
human    GCAAGAACAAGCCTAACATG-AATTATGACAAACTCAG-CCGAGCCCTCAGATACTATTATGT-AAAGGTAGTACACATCCTGATCTTA-----TACTATACAGAGAACCTCCAGA-CAAGAACGATTCCTTCTTG/.../TTGTTTTCTGTTGAGGTTAT--AGATACCAATTT-CCTAGTTTATT----------------------------------------
```

```
cow      C----------ATATTGCTTTTGAGTGTACA---GCATAATGATGCAATATTTGTACGCATTGTGAAATGATC----ACTGTTTTGGTT----TTAACTCAATTTATTTTACAGCTTCAGCAGCTTGGAATTAGAATAAAAGATGTAAA-TGTTCTAAGTGTATTTCTGC
alpaca   CATACAAGATTATATTTGTTTCCTGTATACA---GTATAATGATGCAATATTTGTATACATTGTGAAATGATC----ACTGTTCTGGTTTA--TTTATCCAATTTATTTTCTACCTTCAGCAGTTTGGAATTAGAGTGAAAGATGTAAA-TGTTCTAGCTGTATTTCTGT
dolphin  CATAAA--GTTATATTGGTTTTGGGTGTACA---ACATAATGATGCGATATTTGTATACATTGTGAAATGATC----ACTGTTCTGGT-----TTAATTCAATTTATTTTACAGCTTCAGCAGTTTGGAATTAGGATGAAAGATGTAAA-TGTTCCAGGTGTATTTCTGC
cat      CCTACAGCATTATATTCGCTTGAGGTGTGCA---ACATAACGATTCGATGTTTGTGTACGTTGCAAAACGATCACAGACTGCCTTGGATTATTTTGATCCAGTTTATTTGATAACTTCAGCAGTGTGGAATTGGGATGAAATGTGTAAG-TGTGCCAGCTCTATTTCTGC
dog      CATATAACATTATATTAGTTTCAGGTGTACA---GCATAATGATTTGGGATTTGTATACATTGCAAAATGATC----ACTGTCCTCGTTTATTCTGATCTAGTTTATTTTATAACTTCAGCAGTTTGGAATTAGGATGAAACATGTAAATTGTTCCAGCTCTATTTCTGC
megabat  AATATA-----ACATTAGTTTTAGGTATATATTTAGGCAATGATTCAGTATTTGTTTACATTGTGAAATTATC----ACTATCCTGGTTTATTTTAATCTAATTTTTTTTATAGCTTCAGCAGTTTAAGGTTAGGATGAAAAATGGAAA-TATTTCAGCTGTATTTCTGC
horse    ---------------------------------------------------------------------------------------------TTAATCCAATTTATTTTA--------------------TAGGATGAAAGGTGTAAA-TGATCCAGCTATACTT-AGC
hedgehog---------------------------------------------------------------------------------------------TTAATCCAATTTGTTTTTATACTTCAGCATTTTGGGATTAAGATAAGAGATGTACA-TGTTGCAGCTGTATTTCTGA
shrew    ---------------------------------------------------------------------------------------------TTAACCCGGTATAGGTGATAGTTTCAGC--TTGGGGGTTGGGATGGAAGATATGCA-TGGTCCAGGTTTTTTTCTGC
human    ---------------------------------------------------------------------------------------------TTAATCCAGTGTATTTTGTAGCTTCAGCAGTTTGGAATTAGGATACAAGGTATAAA-TGTTCCGGCTGAATCTCTAC
```

#42785 *(carnivora+perissodactyla) - (artiodactyla)*

```
cat     GATTGTGTTGTATAGTAACTAAAATCAAGGAAAAGCATAAATGCCACTGGAGGACATAT----AATGCTG-ACCATGGGAGTTTAGAGA---------TAACCTTAAGCTAGATCGTTTTTTGTTTTTT------CATTGAGATATAATTGACGCAACAGTATACACTTCCAGGTGTACGACATAACGATTCGATATTTGTATATGTTGTAAAATGATCACCACAGTA
dog     GATTGTAGTATAAAGTAACTAAAATAAAGGAAAGGCATAAATGCCCCTGGAGGAAGTAT----AATGATGTGCAATGGAAGTTTAGAGA---------TAACTTTTAGCTAGATTGTTTTTTTGTTTTTTTTTTAAATTGAGGTATAATTGACACAACAGTATATACTTTTAGGAATACAGTGCAATGATTGGATGTTTGTATATGTTGCAAAATGATCACCACAGTA
horse   CATTGTA-ACTATAGTAACTAAAATAAAGGAGAGGCATAAATGCCA--GGGAGAGGTACAAAGAATGATGTGCCAGGGAAGTTTAGAGAATAGAGAGGTGACTTTTAGCTAGATTGTTATTTTTGTTTT------AATTGAGGTATTATTGACATTATATT----AGTTTCAGGTGTACAACATAATGAT--GATATTTGTATATATTGCAAAATGATCACCACAGTA
cow     GATTATA-TGTATAGTAACTAAAATAAAGGAAAGGCATAAATGCTG-TAGGAGAGCTGT----AATCATGTGCCTTGAAAGCTTAGAGAAAGAAGAGATGACTT----------------------------------------------------------------------------------------------------------------------------
alpaca  GATTGTA-TGTATGGTTACTAAAATAAAGGAAAGGCGTAAATGCCA-TGGGAGAGGTAT----AATAATGTGCCCTGAAAGTTTAGAGAAAGAAGAGGTGACTT----------------------------------------------------------------------------------------------------------------------------
dolphin GATTGTA-TGTATAGTACCTAAAATAAACGAAAGGCATAAATGCCA-TGGGAGAGGTAT----AATGATATGCCATGGAAGTTTAGAGAAAGGAAAGATGATTT----------------------------------------------------------------------------------------------------------------------------
human   GAGTGTG-TATATAGTAACTAAAACATAAGAAAGGCATAAGTGCCA-CAGGAGAGGTATA---AATGATGTG---TGGAAGTTTAGAGAAAGGAGAGATGACTT----------------------------------------------------------------------------------------------------------------------------

cat     AATCCAGCTAACAGCTGTCCCCATATATAGTGACAAAATTTGTGTGTGTGTGTGATGAGATCAGCTAGGTTGTTCAGTGAAAGTTTCATGAAGGAAGTTGTATTTGAATTGGGCCCTGAAAAATATGTCAGAGGTAGGCGTT
dog     AGTCCAGTT---AGCTGTTGCCATACACAGT---------------TATGCCTTTTCTTATTAGCTAGG-TGTTCAGTGGAAGTTTCATGAGAAAATTTGTATTTGCATTAAGCACTGAAAAATACGTAAGATGTAAGCATT
horse   AGTCCAGTTAACATCTGCCACCATACATGGT--TAACAATTTTTTTTTCCTATGATGAGATTAGCTAGATTGTTTAGTGAAAATTTCATGAAGGAAGTTGTATTTGAATTGGGCCCTGAAGAATATGTGAGATGTA-ACGTT
cow     ------------------------------------------------------------TCAGCCGCATTGTTTAGTGACGGTTTCATGATGGAAGTTGTATTTGAATTGGGCCCTGAAAAATATGTAAAATATAGACGTT
alpaca  ------------------------------------------------------------TTAGCTACATTGTTTAGTGGAAGGTTCCTGATGGAAGCTGCATTTGAATTGGGCCCTGAGAAACATTTAAGATGTAGATATT
dolphin ------------------------------------------------------------TCAGCTGCACTGTTTAGTGAACGTTTCATGATGGAAGTTGGGTTTGAATTGGGCCCTGAAAAATATGTAAAATGTAGACATT
human   ------------------------------------------------------------TTAGCCAGATTGTTTAGTGAGAGTTTTATAGAGGAGATGGTATTTGAAATGAACCCTGAAAAATATGTAAGACATACATATT
```

#44900 (carnivora+perissodactyla+chiroptera) - (cetartiodactyla)

```
cat     CACCTTAACTTCAGTCATACAGTGCAAA--ACCTGTGCAACTATGTGTGGCAATCCTGGCAA-------TTTCTTTAAAAGTAAAGAAAAAAATTTGTTGAGATATTATTGAC----------GTATTAATTTCAGGTGTACAACG---TGATTCCATATATGTATGTACTGCAAAATGGTCCCTGTGATAAATT----CCAAGTCCATCAACACACATAATTT----
dog     CACCTC--TCAGAGTTATACAGTGCACC--ACCTGTACAACTGGGTGTGGCAGTCCTGGGAAGGAGCAGTTTTTTTTAAATTAAAGAAAAAATT---TTGAGATACAATTAACA--TAACACTATATTAATTTCAGATACACAACATAATGATTTCA----TATATATGTTGCAAAATGGTTCCCACAATAAATC----TAACATCCATTATCACACATAGCTATAGT
horse   TACCTC--TTACAGTTGTACAGTACATC--ACCTGCACAACCATATGTGGCAGTTCTGGGAAGGAGTAGTTT-TTTAAAAATTAAAAAAATATTTTATTGAGATATGATTGACATATAACATTATGCTAGTTTCAGATGTACAACATAATGATTTGAGGTTTGGGTATATTGCAAAATGATCCCCACAATAAGTCTAGTTAACATCCATCACCACGCATAGTTACAAA
megabat CACCTC--TTAGAGTGATGCAGTGTACC--GCCCGCACAACTGTATGTGGCCCACCTAGAAAGAAGTAGCTT-----AAATTTTTTAAAAATTTTAATTGAGATATAATTGATATCTAACATTGCCTTAGTTTCAGGTGTACAATGTAATGATTCAATATTTGTATATGTTGCTAAACGATCCTCAAAATAAGTCTAGCTAAGAAAGATCACCACACTTAGATAAAAA
cow     CAGCCC--TTAGATTTACACAGGGCA-C--ATCTGCACAGCTGTATGCAGCAGTCCTAGAAAGA---AGTGT------------------------------------------------------------------------------------------------------------------------------------------------------------
alpaca  CATCTC--TTAGATTTGCGCAGTGCA-C--ACTTGTGCAACTGTATGTGGCGGTCCTAGAAAGAAGTAGTGC------------------------------------------------------------------------------------------------------------------------------------------------------------
dolphin CACCCC--TTAGG---GCACAGTGCA-C--ACCTGTACAACTGTATGCAGCAGTCCTAGAAAGAAGAAGTGT------------------------------------------------------------------------------------------------------------------------------------------------------------
hedgehogTACCTC--TTAGAGTTATGAAATGCAAC--ACCTACATAACCATATGTGGCAATCCCAGGAAGGAGAAGCTT------------------------------------------------------------------------------------------------------------------------------------------------------------
shrew   CGCCCC--TCAGAGCTCCACGGGGGGTCTAGCCTGCACACCCAGGTGTGGCCCTGCTGGAAATGCCTGGTCT------------------------------------------------------------------------------------------------------------------------------------------------------------
human   -GCATC--TCAGATCTACACAGTGTACC--GCCTATACAGCTGTCTGTGGTAGTCCTGGGAGGAGGTAGTTT------------------------------------------------------------------------------------------------------------------------------------------------------------

cat     ---TTTTTCT---TGTGATGAGAACTTTTCAGATCTACTCACTTAACAACTTTCAAATCTGCAACACAGCATTATTAACTGTAGTTAATAGGAGCTGCTTTTAAATCTCCTTTAGAATAGAAAGTTAGCACTAATCCAATGGTGTCTCTTTCTTGTT--CTGGTCT
dog     TTCTTTTTCT---TGTGATGAGAATTTTTAAGATCTGCTCACTTACTAACTTGCAGATATGCAATACAGTATTATTAACTATAGTTAACGGGAGTTACTTTTAAGTCTCCTTCGGAAGAGAAAGTTGGCATTAACACAA---TGTCTCCTCCTTGTT--CTAATCT
horse   T--TTTTTCT---TGTGATGAAAACGTTTAAGATCTACTCTCTTAGCAAATTTCTAATATATAATACAGTATTACTAACTAGAATTAATA---GTAGTTTTTAAATCTCCTTC-GAAGAGAAAGTTGG-ATTAATACAATGTTGTCTCCTCTTTGTTCCCTGATCT
megabat CTCTTTTTTTGTGTGTGACAAGAACTT---------------TTAGCAACTTTCAAATATATAATAGAGTATTATTAACTGTCGTTAACAGG-GTAGTTCTTAAATCTCCTTTGGAAGAGAAAGTTGGCATTAATCCAATGTCATTTCCTCTTTGTTCTTT-ATCT
cow     ----------------------------------------------------------------------------------------------------TTAAATCCTCTTTGGAAGAGGAAATTGACATTAACCCATTGTTGTCTCTTTTCCATTTCCTGATCT
alpaca  ----------------------------------------------------------------------------------------------------TTAAATCTTCTTTGGAAGAGAAAGTTGGCATTAATCGAATGTTGTCTTCCTCCCATTCCCTGATCT
dolphin ----------------------------------------------------------------------------------------------------TTAAATCTTCTTTGGAAGAGAAAGTTGGCATTAATCCACTGTTGTCTCCTTTCCATTTCCTGATCT
hedgehog----------------------------------------------------------------------------------------------------CTAGAGCTCTTTGGGAAGAGAAAATTGGCATTAACTTGAAAGTATCTCCTCTTTGCTTCCTGCTCT
shrew   ----------------------------------------------------------------------------------------------------TTAGGTCTTCTTTGTCTGGGGAAATTTGGCATTGATCGATGGTCTCTTTCCTCTGTGCCCTGATCT
human   ----------------------------------------------------------------------------------------------------TAAAATCTCCATTGGAATTGAAAGTTGGCATTAATCTCATGTTGCCTCCTCTTACTTCCCTGGCCT
```

#75125 *(carnivora+perissodactyla) - (chiroptera, cetartiodactyla)*

```
cat     GGACAGAGGAGAGGGGACTTCACCGAGCCACATCTCCCGGTAAGCAC------CAGGCAG-------------ACATGGGTGCTTCTGGGCACCTG-CTTTACTCAGTGA-TGCCGGGCAGGGAGGTCCATGAGGAAATGTCAGCCAGGGAAATCTGAGAATGCCCCTTATC--------------CATTTTCGCACAAGTCCTTTGGTCTCACTGTCCTCTCCTAT
dog     GGACAGAGGAGAGGGAACTGCACCAAGCCACATCTCCCGGTGAGCAC------CAGGCAGACACTACCAGGAAAACACGAGTGCTTCTGGGTACCTG-CTTTATTCACTGA-GGGGATGGAGGGAGATCCATTAGGAAATGTCACCCAGGGAAATTTGAGAATGCTCCTTCTC--------------CATTCTCTCACAAGTCCTTTGCTCTCACTGTCCTCTCCTGT
horse   GGACAGAGGAGAGAGGACTTCACCGAGGCTCTTGTCCCGGTAAGCAC------CAGGCAC-------------ATGCGAGTGTTTCTGAATATCT----CTGTCTAGTGATTGGGGTGGAGGGAGATCCATTAGGAAATATCA-CCAGGGAAATTTGAAAAT--TCGTTCTCTTTCACTGCCCCTTCGTTCTCTCACAAGTCCTTTGGTCTCGATGTCCCTTC----
microbatGAACAGAGGAGAGTGGATTTCCCCAAGGCACATGTCCCGGTAAGGAC------CTGCA---------------TCATAAGTGCTTCTGAGTAGTGG-CTATTCTCAGTTATTGGGATGGAGGGGGATCCATTAGGAAATTTCACCCAGGAATATAT-AAAATGCTCATTCTCTTTCATTGCTCCTCCATTCTCTGACACATTCTTTGGTTTCACTATCCTTTC----
cow     GGACAGAGGATCGGGAACTTCACCGAGGCCCTTGTCCCGGTAAGCAC------CAGGCAG-------------ACAGAAGTGCTGCCGAGAATCTG-CCCTTGCCAGCAATGGGGATGAATGGAGTTTCATTGGGAAACCTCACCCAGG-----TTGAAAATGCGCTTTCTCTTTCACTACACCTCCATCCTCTTAAAAGTCTTTTGGTTTCCGTGTCCCTTC----
dolphin GGACAGAGGAGCAGGAACTTCACCAAGGCCCTTGTCCCGGTAAGCAC------CAGGCAG-------------ACACAAGTGCTTTTGAGAATCTG-CTCTCTCCAGTGATTGGGATG-ACAGAGATCCATGAGGAAACGTCACCCAGGGAAATCTGAAAATGCTCTTTCTCTTTCACTGTCCCTCCATCCTCTCACAATTCTTTTGGTCTCCATGTCCCTTC----
shrew   GGGCCAAGGAGCAGGGGGATCAGCCCGGCGCCCGTCCGGGTAAGACGCCCCCGCGGGCAG-------------AAGGAAGAGTTTCAGGGGACCTG--------------CGGAGTGGGTGGGCACGGCCGAGGGGACC-------------------ATGCTGTCTCAGCACTGCCCGGGCGCCACTGGCTCTCAGCGGCCTGAGTTGAGGAATTCCTGC----
human   GGACAGAGGAGAGGGGCGCTCACCAGGGCGCTTGTGCAGGTGAGCGAGCCC--CAGGCAA----------------AGAGCGGTGCTGTGACTCTACCTCTATTCAGTGACTGGGGTGGAGGGAGGTAGGTTAGGGAGTGTCACCCAGGGAAATTGAGAGATGCTCCTTCCCTCTTTTTGCCCATCCATTCTCTGACAAATCTTTTGGTCCACACACTCCTTC----

cat     GTATGTATGTCTGTGTTTTTTCCTTTTCTCTTCCTTTTATCTATCTGCTGACTGACTTACTCACTTATTGGTTTACTTACTTTCCACTGAGATACTAACTGTGTTAGTTTCAGGTGTACAACATAGTGACTCAATATTTGTATGTATTGCAAAGTGATCAC-----------------------------------------------------------CACAGTAAATCT
dog     GTTGTATATGTTTCTTCCTTCATCTTTTTCATCTGTCTACGTATTCATTTGCCAACTTACTTAGTT----------TTGAGATGTAACTGACATGATACCATGTTAGTTTTAAGTATACGACATAATGGAAAAAAAAAAGTATACGACATAATGGTTCAATGTTTGAACCATTATGTGATCACCATGTTTAATGTGGATCACCATGTTTAATGTGGATCTTTAATGTG
horse   ------------------------------------------------------------------------------------------------------------------------------------------------------------------------------------------------------------------------------------ATCTTTACCTAT
microbat------------------------------------------------------------------------------------------------------------------------------------------------------------------------------------------------------------------------------------------------
cow     ------------------------------------------------------------------------------------------------------------------------------------------------------------------------------------------------------------------------------------------------
dolphin ------------------------------------------------------------------------------------------------------------------------------------------------------------------------------------------------------------------------------------------------
shrew   ------------------------------------------------------------------------------------------------------------------------------------------------------------------------------------------------------------------------------------------------
human   ------------------------------------------------------------------------------------------------------------------------------------------------------------------------------------------------------------------------------------------------

cat     GATTAACATCCGTCACCACACA-GGTACAAAACAATTTTTTTCTTGTGATGAGAACTTTTAAGATTTACTGTCTTAACTTTTTTGTTAAATTTATTTTGAGAGAACGAGTTGGGGAGGGGTGGAGAAAGCGTGAGGGAGAGAGAATCCCAAGCGGACTCTGCACCCTCAGTGCAGAGCCCGACGTGGGACTTGATTCCATGAACGATGAGACCATGACCTGAGCCGAA
dog     GATTAAGATCCATCACCACAGA-GTTAGAAAAATATTTTTTCCTTGTGATTAGAACATTTAAGATTTACCGTC------------------------------------------------------------------------------------------------------------------------------------------------------------------------
horse   AGTTAACGTCAATTACCGCACATAGTTACAAAATTTTGTGTGTGTGTGATGAGAATTTTTAAGGTTTACTCTC------------------------------------------------------------------------------------------------------------------------------------------------------------------------
microbat------------------------------------------------------------------------------------------------------------------------------------------------------------------------------------------------------------------------------------------------
cow     ------------------------------------------------------------------------------------------------------------------------------------------------------------------------------------------------------------------------------------------------
dolphin ------------------------------------------------------------------------------------------------------------------------------------------------------------------------------------------------------------------------------------------------
shrew   ------------------------------------------------------------------------------------------------------------------------------------------------------------------------------------------------------------------------------------------------
human   ------------------------------------------------------------------------------------------------------------------------------------------------------------------------------------------------------------------------------------------------

cat     ATCAAGAGTCAGACATTTAACCAGCTAAGCCACCCCACTCCCCTCTTAGCACTTTTCAGTATAATACAGACTTATTAATTATAGTCACCATGCTGTACCCTACAGCCC-AGGACTTACTTGTTTCCCAACTGGAAGTTTGTACCTTTTGACCCCCTTCAC--------ACCTATTTTGCCCACCCGCCACCTCTGACAACCATCA-CAGTCTGTTCTCTGTATCCACA
dog     ---------------------------------------------TTAGCAACCTTCAGTGCATTACAAAATTATTAACTATAGTCACCATGCTGTGTGTTACATCCCCAGGGCTTATCTCTTTCATAACTGGGAGT---TACCTTTTGACCCCCTTCAC-----------ACCCACATCCACCCACCGCCTCTGACAACCACCACCAATCTGTTCTGGGTATCCATG
horse   --------------------------------------------AGCAACTTTCAAATACGCAGTACAATGTTATTAACTATAGCCATCAGGCTGTACTTTATATCCCTAGGACTTATTTATTTTATAACTGGAAGTTTCTACCTTTTGACCCCCTTCACCAATTTCACCCACCCTCCACTCTCTCCCACCTCTGACAACCAT---CAGTCTGTTCTCTGTATCTGTG
microbat------------------------------------------------------------------------------------------------------------------------------------------------------------------------------------------------------------------------------------------------
cow     ------------------------------------------------------------------------------------------------------------------------------------------------------------------------------------------------------------------------------------------------
dolphin ------------------------------------------------------------------------------------------------------------------------------------------------------------------------------------------------------------------------------------------------
shrew   ------------------------------------------------------------------------------------------------------------------------------------------------------------------------------------------------------------------------------------------------
human   ------------------------------------------------------------------------------------------------------------------------------------------------------------------------------------------------------------------------------------------------

cat     AGCTCAGTTCTTTTGTTTTGTTTT----GTTTTGGGTGGTTGTTTTGTTTTGTTTTGTTTAGATTCTACATAGAAGTGAGATCATGCAGTCTGTCTGTCTCTGTCTTATCTTAGCATTATGCCCTCAAGACCCATCTGTGTGGTTGAGTGGGAAGATTTCCTTCTTTTTATTTTTTTTACGGCTGAATAATAATCTACTGTGTGTGTATGC----CACATTTTCTTTAT
dog     AGCTCAGTTGTTTTGAGTTTTTTTGGCGGGGCTGGGGGGAGGTTTGGTTTTTGTATTTCGGATTCCACACTGAAGTAAGATATGATACGTCTTTCTTTGTCTTATCTTACTTAGCAATATGCTCTC-------------TATTCCTGTTGCCAAAAATGGGAATATTCCCTTTTTTTATGGCTGAATAATACTCTGTTATATAT---TACATGCCATGTTTTCTTTAT
horse   AGCTTGGTTTTGCTTTGTTTTTTTTAAGATTCCACATATAAGTGAGATCATTCAATACTTGTCTT-----------------------TCTCTGTCTGACTTATTGCACTTAGCATAATATGCCCTCAA---GTTCCATCCGTTTTGTCAGAAATGGCAGGATGTCCTTCTTTTTTACGGCTGAATAATATTCCATTCTTTATATATACCACATTTTCTTTTTCCAT
microbat------------------------------------------------------------------------------------------------------------------------------------------------------------------------------------------------------------------------------------------------
cow     ------------------------------------------------------------------------------------------------------------------------------------------------------------------------------------------------------------------------------------------------
dolphin ------------------------------------------------------------------------------------------------------------------------------------------------------------------------------------------------------------------------------------------------
shrew   ------------------------------------------------------------------------------------------------------------------------------------------------------------------------------------------------------------------------------------------------
human   ------------------------------------------------------------------------------------------------------------------------------------------------------------------------------------------------------------------------------------------------

cat     TCATCTGTCAAAGGACACTTAGATTGATTCCA-TACCTCGGCTATTGTAAATAATGCTGCTGTGAACGTGGGGGTGTGGATATCTTTTCAAGGTAGTGTTTTTGCTTTCTTTGGGTAAATCCCCAGGAGCAGAATTGCTGGGCTTCGTGTGTAAATTTGACCTGTCCAC--ATGAG--CTTTGTCCCATTCTTTCATACTCTGCACCCCTGATTGTAC-CCTTCTGTT
dog     TCATATATTGAGGAACACTTAGATTGCTTCCATTGCCTTGACTATTGTAAATAATGCTGCTGTAAACATGGGAGTGTGGATATGTTTTTAAGGTAGTGTTTTCATTTTCTTGGGATAAATAGCCAGAAGCAGAGTTTCTGGGT---GTCATCAAACTTGACATGTCTAC--ACGAG--CTCTGTCCCATTCTTTCATATTCAACATTCCTGGTCATACTCTTTCTTTT
horse   TTATCTTTCAAAGGACACTTAGGTTGTTTCCA-TATCTTGGCTACTGTAAATAATGCTACAAGGAACGTGAGGGTGCAGATATCTTTTTGAGTTACTGTTTTTGTTTTCTTGGGATAAATACCCAGAAGTGGAATTGGTAGGCTTCATTTTTAAATGTCACATGTCCAC--ATGGTCTCTTTCTCCCATTTCTCTGTACTTAACATTCCTGATCATATTCCTTCTGTC
microbat------------------------------------------------------------------------------------------------------------------------------------------------------------ACCTTTAAATTTGACATGTCCAC--ATGGA-ACTTTTTTCTATCCTTTTAAATTCAACATTCCTGATTATATTTTTTCTTTC
cow     ------------------------------------------------------------------------------------------------------------------------------------------------------------ATCTTTAAATTTGAGGTGGCCAT--GTGGGCTCTTTGTCCCATTCTTTCCTATTCAACATTCCTGGTCATACTCTTAATATC
dolphin -------------------------------------------------------------------------------------------------------------------------------------------------------------TCTTTAAATTCGACATGTGTCCAAATGGGTTCTTCGTCCCATTCTTTCCTACTCNNNNNNNNNNNNNNNNNNNNNNNNNNNNN
shrew   -----------------------------------------------------------------CAGGGGTGAATTGACGGATCCCTCTGGCCTTGTCACTGCTTTCATGGGACAGACAGAGAGC----------------------------------------------------------------------------TGCCCGGTGTTCCTGGTCATGCCCTTA-----
human   -----------------------------------------------------------------------------------------------------------------------------------------------------------------TGACTTTCACATTCCCATTATTGGGCTCTTTGTCCCATTCTTTCATATTTGATGTTTCTGATCATCCGCTTTCTGTC

cat     GTTATCCGTGTT
dog     GTTATCACTGTC
horse   ATTATCAACGTC
microbatATTAATGTTG--
cow     ATTATCAATGTC
dolphin NNNNNNNNNNNN
shrew   ------------
human   ATTATCAACATC
```

B-1060 – L1MC5 *(carnivora+perissodactyla+chiroptera) – (cetartiodactyla)*

```
dog     ----TGTCTAGTGATATCATTTAGCATATATTTTGGAGTACACCTAATATCTAAATAATGCTTATTATCAGTATCACAAAATTCTCTCTAGAATGGCTGTATTAGTTTATATTTTCCCTAACAAGAGCAGAAGGCTTCTTTCATCCCTGTTTCCTTACCAGTGTTTGGCATTATCTAACTCTTCAGCATTTTCC---------AGTACAGAGTAACATATCA---CTG
panda   TATTTGTCTAGTGTTATCATTTAGTATATATTTTGGGGTACAGCTGATATCTAAATAATGCATATTAATAGTATCACAAAATTCTCTCCAGAATAGCTGTATCAGTGTATATTTTTGCTAGC-AGAGCAAAAGGCTTCTTTTCTCCCTGTTTCCCTACCAACATTTGGCATTATCTAACTCTC--ATGTTTTCCAGCCTAGTAGGTATAGAGTAACGTATCGCTGCTG
microbatTATCTGTCCTGTGATATCATATAGTAT-TATTCTTGGGTAAAGCTAAGTTCTCAGTGCTGCATATCAGCAGTTTCGTAAAGGTCTCTCCAGAATGGCTGGACTCGTTAACATT-TCACTAGC-AGAGCAGGAG--CTCCCTCTTCCCTGTTTCACCAGCA--CATGCGGCTTATCCAATTCTTTCATGTTTCCC-GTCTCATGGTTATAAAGTAATATGTCAGTGCTA
horse   TATTTGTCCAGTGGTATCATTTACTATATATTCTTGGGGATAGCTAATATCTAAATAATGTGTATTAACAGCATCACGACATGCTTTCCAGAAGGGCTGCACCGGTTTATCTTCTCACCAGC-AGAGCAGGAGGCTTCCTGCATCCCTGTTTCCTCACCAGCATCTGGTGTTACCTAACTCTTTAGTGTTTTCCAGTCTAATAGGTGTAAAGTAGCGTATCAGTACTG
cow     TATTTGTCTAGTAGTATCATTTAGTATACATTCTTGAATATAGCTAAGAGCTTAATAATACATGTTAACAATATAACAGGGTA-------------------------------------------------------------------------------------------------------------------------------------------------
dolphin T-TTTGTCTAGTGGTATCATCTAGTATACATTCTTGGGTATGGCTAAGAGCTCAAAAATACATATTAACAATGTAACACGTG--------------------------------------------------------------------------------------------------------------------------------------------------
alpaca  A-TTTGTGTAGTGATACCATTTAGTATACATCCTTGGGTAGAGCTGAGAGCTCAGTAATGCATATTACCATTATAACACATGG-------------------------------------------------------------------------------------------------------------------------------------------------
pig     TATCTGTTTAGTGGTATTATTTGGTAAACCTTTTAGGGTATAGCTAAGAGCACAATAATACATATTAA---TGTAACACCTG--------------------------------------------------------------------------------------------------------------------------------------------------
mouse   TATTTGCCCAGTGACATCTTTTA-TATACATTCTTAGACAGAGCTTGGTT----GGGATACATACTG------AAACACA----------------------------------------------------------------------------------------------------------------------------------------------------

dog     TTTAAATTTGTCTTACTGTGATTATTA----------------TCTTCAAATGCTTGTTACTTTTTTT--------TTCTGTGAAGTCCACGTGCATGGCCTTTGTGCATTTTTCTGTAGCAGTTGCTGCTGTTTTGTT-ATTGATTTGCAGGGGATCATTGTATATTTTGGATATTAGCCCTGGTTGGTTACAGACAATGC----AGATATCTTCTCCCATTCTGTT
panda   TTTATATTTGTCTTACTGTGGTTATTA----GTTTGATTACCTTCTTCAAATGCTTGTTACCTTTTTTGTT-----TTCTGTGAAGTCCATGTGCTTGGCCTTTG-----------------GTTGCTGCCTTTTTGTT-ATTGATTTGCAGGGGTTCACTGTATATTTTGGATATTAGCCCTGGTTGGTTTCAGACAATGC----AGATATCCTCTCGCATTCTGTT
microbatTTTCCATTTGTCTTACTGTGATTATTATTGAGTTCGATTATC-TTTTCAGATGCTTGTTACGGTTTTTGTTTTCTCTTATGTAAAGTCCATGTTTATATCTTTTGTGCGTTTTTCTCTAGAGGTTGCTGCCTTTTTGTTAATTGATTTGCAGGAGTTGGGTGTATATTCTGGATATTATTGCTGGCTGGTTTTAGACAATGCCAATAAGTACCACCTCTCATTCTAGT
horse   TTTCAATTTGTTTTACTCTGACAATTATTGAGTCTGATTATC-TCTTCAGGTGCTTGTTCCTTTTTCTGTTGTGTCTTCTGTAAAGTCTGTGTTTATGTCTTTCGTGCATTTTTCTCTAGGAGCTGCTGCCGTTTTGTT-ACTGATTTGCAGGAGTTCATTGTATATTCTGGATATTATCCCTGGTTGGTTTTAGACAATGC----AAATATCCTCTCCCATCCTTTT
cow     ------------------------------------------------------------------------------------------------------------------------------------------------------------------------------------------------------------------------------------
dolphin ------------------------------------------------------------------------------------------------------------------------------------------------------------------------------------------------------------------------------------
alpaca  ------------------------------------------------------------------------------------------------------------------------------------------------------------------------------------------------------------------------------------
pig     ------------------------------------------------------------------------------------------------------------------------------------------------------------------------------------------------------------------------------------
mouse   ------------------------------------------------------------------------------------------------------------------------------------------------------------------------------------------------------------------------------------

dog     ACCTGTTTATAAGAGTTGTCCATGGTCTACTTTAAAA--AAAAAAAATCTTTAATTTTGATGTAATGAAATAATAAAATGTTTTCCATTATGGTTTTAGAATT---GTGTATAGAGAAGTCTCTCCCTGTCCATGGTCACAGACATAATTCGTACGTTTTATTCTATTATATTTTAAGTCTTCACATTCTCTCTTATGGCTTCAGCTCAACCTGCTGTTATATATGGC
panda   ACTTGTTTATAAGAGCTGCCCATGGTTTACTTTGTTT---AAAAAAATCTTTAATTTTGATGTAATGAAATAGTA-----TTTTCCATTTTGGTTTTTGGGATT--ATATATAAAGAAGTCCCTTTTTGTCCATGGTCACAAACATAATTCATACATTTTATTCTATTAAATTTAAATCTTCACATTTCTCTTTTATGGCTTCAACTCAGCCTGCTGTTTTATGTGGT
microbatATTTGTTTATAAGAGTCGCCCATGGTCTACTTCGTTT---AAAAACATCTTTAATGTTGATGTAAGGAAATAATAGAATATTTCTCATTA-GGTATTTGGGGTTTCATAT-TTTAAAAGTCTTTCCCTATCCTTGGTCACAAAAATAATTCTTATATTTTATTC--CTTAATCTAAAGCTTTAC-TTTTTTTTTTATGACTTCAACTCA-GCTGCTGTTTTATATGGC
horse   ATTTGTTTATAAGAGTTGTCCAGGGTCTACTTTGTTTAAAAAAAAAATCTTCAATTTTGATATAATGAAATAATAAAATATTTTCCTTTATGGTTTTTGGGGTTTCTTATATAAAGAAGTCCTTCTCTGTCTCTGGTCACAAAGGTAATTCTTACGTTTTATTCTATTAAGTTTAAAGCTTCACATTTCTCTTTTATGGCTTCAACTCA-CCTGCTGTTATCTATGGC
cow     ------------------------------------------------------------------------------------------------------------------------------------------------------------------------------------------------------------------------------------
dolphin ------------------------------------------------------------------------------------------------------------------------------------------------------------------------------------------------------------------------------------
alpaca  ------------------------------------------------------------------------------------------------------------------------------------------------------------------------------------------------------------------------------------
pig     ------------------------------------------------------------------------------------------------------------------------------------------------------------------------------------------------------------------------------------
mouse   ------------------------------------------------------------------------------------------------------------------------------------------------------------------------------------------------------------------------------------

dog     ATTAGAACAGGATCCAGTTTTACTTTTCTTTATATAGTGAGCTATTTTTTT----CTAATAGCTATTGAGTAATCCATAATCCTTCCCATTCAAATACTTATTAACA---ATATAATATGCG--TATTCCTCAAATCCTTATTTTACATAT-------------------TCTATGCATTTCTTTCCT
panda   ATTAGATCAGGATCCAGTTTTATTTTTTTTTATATAGTGAATTATTTTTTTCCTAATAGTATCTGTTGAATAATCCATAATCCTTTCTGTTCAAATACATATTAACA---ATATAACACATG--TATTCCTCAACTCCTCATTTTATATAT-------------------TGTATACATTTCTTTCCT
microbatATTAGGTCAAGACCCAGTTTTATTTTTCGTTATATAGTGAGCCATTTTTTT-CTAATAACACCTATTAAAAAATCCATAATACCTCCC--TCAAATACACATTAATAATAATATAACACACGTGTGTTCTTTAAAGCCTCATTTTATATAC-------------------TGTATATGTTTCTTTCCA
horse   ATTAGGTCAGGATCCAGTTTTACTT----ATGTATAGTGAGGAATTTTTTC--TAATAACACCTATTAAATAATGCATAATCCCCCTC-----AAGTCATATTAAAA---ATATAACACATGTATATTCCTTAAATCCTTATTTTATATATATGTATATATACACACACACACACACATTTCTTTCCT
cow     ----------------------------------------------------------------------------------------------------------------------------TATTCCTCAAATCCTCATGCTACATG--------------------TTGATACATTTCTTTCCT
dolphin ----------------------------------------------------------------------------------------------------------------------------TATTCCTCAAATCCTCATTCTACATA--------------------TTGATACATTTCTTTCCT
alpaca  ---------------------------------------------------------------------------------------------------------------------------GTATTCCCCAGACCTCATGTTACATA--------------------TTGATACATTTCTCTACT
pig     ----------------------------------------------------------------------------------------------------------------------------TATTCCTCAAATCCACAT-TTACATA--------------------TTGTTACATTTCTTTCCT
mouse   ----------------------------------------------------------------------------------------------------------------------------TGTTTCACAATTCCCC---TTATATAAGT-----------------TGTTTGCATTTCTCCCCT
```

B-1676 – L1MA9 *(carnivora+cetartiodactyla+chiroptera) – (perissodactyla)*

```
cow     TTTCAAATCCCTAACCTTGAATGCAGGGTCCTGCCCTGTTTGACACCGCATTGCTTTGTGTG-----TGTGTGTGTGTGTGTGTGTGTGTGTGTGTGTGTTTTAATTGAGGTATAATTGATACAACAT-----TACCTTAGATTCAGGTGTACAACTTAATGATTTGGTATTTGTATACATTGCAAAATGATCACCAACAACAAACAAATAAATCCCACGAT-ACTCT
dolphin TTCCAAACCCCTGACCTTGAATGCAGGGCCCTGCCCCATTTGACACCACACGAATGCTTCT------------------------------TTTTTTTCTTTTTGTTGAAGTGTAGTTGATGTAACCTTAGAGTACCTTAGAGTCAGGTGTACAACTTAATGATTGGATATTTGTATACATTGCAAAATGACAACCA---------CAATAAATCCCATGATAACTCT
alpaca  TTCCAAATCCCTGACCCTGGATGCAGGGCTCGGTCCCATTTAGCCATGCAAAGGAACTCTTC-----------------------------TGTTTTTCTTTTTATAGAGGTATAACT-CTATAGCAT-----TATATTAGTTTTAGGTGTACGACTTAATGATTTGATATTTGTATACATTGC----------------------AAGTAAGTCCCACTATTACTTT
megabat TTCCAAACCCCTGACACAGAATGCTGGGCCCTGCCGCATTTGACCGTGATAATA-------------------------------------ATTCTTTTTTTTTATCGAAGTGTAATAGA-------------CATATTAGTTTCAGGTATACAGTATGATGTTTCAATATTTGTATATATTGCAAAATGATCA------------CAAGAAGTCCCACACTGATATT
micro   TTCCAAACCCCTGACACCGAATGCAGGTCCCTGCCCCATTTGGCATCACACTAA-------------------------------------CCTTTTTTTTTTAATTGAGGTGTAATTAACACAACAT-----TATATTAGTTTCAGGTGTACAATATAAGGATTCAATATCTGTATATATTGCAAAATGATCACCA---------CAAGAAGTTCTACACTGACTCT
dog     TTCCAAATGCCTGACAGTGAGTGC-GAGCCCTGCCCCATTCGGCACCGCACTAACTCTTCTTCTCCTTCCCCCCCCCCTTCCTCCTCCTCTTCCTTTTTTTCTTCTTCAAGTATAGTTGACATGACAT-----CACGCCAGTTTCAGGGGTACAATGTAATGATTCACTATT-----------GCAGACGTTCACCA---------CAGTAGGCCCCGCGCTCACTTC
panda   TTCCAAAAGCCTGGCAGTGCATGCGGGGCCCTGCCCCATTTGGCACCCTGGTAACTCTTCTT---------------CTTTTCCTTCTCCTCCCCTTTTTATTTCTTCAGGTATAATTGACATAACATTA---TATATGGGTTTCAGGTGTACAATGTAATGATTCAATATTTGTATATATTGCAAAATGTTCACCA---------CGATAAGTTCCATGCTAACTCT
horse   TTCCAGACCCCTGACACTGCATGCAGGGTGCCCCCCAGTTTGGCACTGGGC------------------------------------------------------------------------------------------------------------------------------------------------------------------------------------TAACTCT
human   TTCCAAACCCCTGATATTGAATGCAGGGCCCAGCACCATTTGGCACTGCAC------------------------------------------------------------------------------------------------------------------------------------------------------------------------------------------TAACTCC

cow     TCTTAATCTG-TCTTCCTTTATTCCACCTCTTTGTC
dolphin TCTTTGTTCA-TCTTCCTTTATTCTAACTGTTTGTC
alpaca  TCTTGTTCGAGATCTTTTTTATTCCAACTCTTTGTC
megabat TCTTAT-----TTCTTCTTAATTCTAAGTTCTTGAT
micro   TCTTAT-----TTTTGCTTTATTCCAACTCTTTGAC
dog     TCTTGCTCTT-TTGTTCTTTTTTCCAACTCTGTCTT
panda   TCTTACT----CTGTTCTTTATTCCAACTCTGTATT
horse   TCTTATTCTA-TCTTTCTTTATTCCAACTCTTTGTT
human   TCTTAT-----TCTTTCTTCATTCCAACTCTTTGTC
```

B-1522 - L1M3b

```
cow      CATAGAATACAGAGTTATACATTTTTCAA--------AGAGGCAACGGATCATGATAGTT-TCTAGAATTGTAATTTCTATTAAGAAATCACAGACAAGAG-------------------TGATGTCACCAAGTTGGAGACGTAGGTTGTTCCCAACTT-GCTTCCCCTTGCAGGA---ACAACTAACAACTATTCAGGGACAGGACACTATTGAAAACAATTCCAGA
dolphin  CCTAGAATAAAGAGTTATAAAATTTTCAA--------AGAGGCAACTTATCTTAATAGTT-TCTAGAATTGGAATTTCTATTGAGAAATCACAGAGACAAGAG-----------------TGATGTCACCAAGTTGGAGGCATAGGTTGTTCCCAACTT-GCTTCCCCTTGCAAGAAACACAACTAACAACTATTCAGGGACAAGACATCATTGAGAA-AATTATAGA
pig      CCTAGATTTCAGAGTTATAAAATCTTGAA--------AGAAGGAACTG-TCTTGGAATTT-TATATAATTGAAATTTCTATTAAGAAATCCCAGAAAGAGGGAATTCCCGTTGTGGCACAAAAATCCACCAAGTTGGAGACATAAGTTGTTCCCAACTT-GCTTCTCCTCATAAGA---AGAACTAACAACTATTTAGGGACAAGGCTTCATTGAGAA-AATCCTAGA
megabat  CCTAGAATGCAGAGTCATGAAATCTACAA--------AGAGG--------CCTTATAGTT-TCTATAATTAGAATTTCTATTAAGAAATCACAGAGACAGAAG-----------------TGACTTCACCAAGATAGTGACATACTTTGCTCCTGACCTTGCTCCCCATCACAAGA---AGAACTAACAACTATTCATGGATAAGACACCGCTGAGAA-AATCCTGGA
microbatCTTAGAATGCAGACTCATGAAACCTATAA--------AGTGGAGACTGATCTTGATAGTT-TCTAGGATTAGAATTTCAATTGGAA--------TTAACATTT-----------------CTGTGTCACCAAGATGGTGAAATAGTTCACTCTGGCCTTTGCTCCCCTTCACAAGA---AGAACTAACAGCTATTCATGGACAAGACATCACGGAGAA-AATCCTGGA
horse    CCTAGAACACAGAGTCACAAAATCTTTGA--------AGAGGCAACTGATCTTGACAGTT-TCTAGAATTAGAATTTCTATCAAGAAATCATAGAGAAAGAAG-----------------TGACATCACCAAGATGGTAACATAGGTCGTTCCTAACTTCTCTGCCCCTCGCAAGA---ACAACTAACAGCTATTCATGGACAAGACATCACTGAGAA-AATCATAGA
dog      CCCAGAATAT--AGTCATAAAATTTTCGGAGTTTTACAGAGTTAACAGAGTTTAGTAATT-TCTAGAATTATATTCCT------------------------------------------------------------------------------------------------------------------------------------------------ATTAAA
panda    TCTGGAATGT--AGTCATAAAATTTTCAA--------AGAATTAACAGATTTTAATAATT-TCTAGAATTAAATAGTTTCA------------------------------------------------------------------------------------------------------------------------------------------AGAATTAGA
human    CCTGGAGTATAGAGTCATAAAATCTTTAA--------GGAGGCAACTGATTTTGATAGTTGCTTAGAATTCGAATTTCTATTAAGAAATC------------------------------------------------------------------------------------------------------------------------------------------

cow      AATCACAG-AGATGACTTTTACGTTATTTTTT--CCTCTGGCCTTTCCTTGTCCTTTTGTGTGAAATTTACTAATCATCCCAGTCATCATATTTTATGCCA
dolphin  AATCACAG-ATACAACTTTTATGTAATCTTTT--CCTCTGACCCTTCCTTGTCCTTTTGTGTGAAATACACTAATCATCCTGGTCATCAAATTCTTTGCCA
pig      AATCACAG-AGATAACATTTATGTAATATTT---CCTCTGAC----CCTTGTCCTTTTTGGTGAAATATACTAATCATCCTGGTCTTCAAATTTTATGTCA
megabat  AATCACAG-GAATAACTTTTATGTAATTTTT---TTTCTGAC----CCTTGTCCTTTTGAGTAAAATATACTAATCATCCTGGTCATCAAATTTTATGCCA
microbatGATCACAG-AGATCATTGTTATGTAGTTTTTTTCCCCCTGAC----CCTTGGCCTTTTGGGTGAAATCTACCAATCATCCTTGTCATCAAATTTTATGCCA
horse    AAACACAGAAGATAACTTTCATGTAATTTGT---CCTCTGAC----ACTTGTCCTTTTGGGTGAAATATACTAATCATCCTGGTCATCAAATTTTATGCCA
dog      AACCACAG-AGATAACTTTTATGTAATTTTATT-CCTCAGAC----CTTTGTCCTTTTGGCTAAAG----------ATACTAATCATCAAATTTTATGCCA
panda    AACCACAG-ACATAACTTTTATGTAATTTTATT-GCTCAGAT----GTTTGTCCTTTTGGGTAAAA-ATATTAATCATCCTTATCATCAAATTTTATGCCA
human    --CCATAG-AGATAATGTTTACATAATTTTTT--CCTCCGAT----CATTGTCCTTTCCTGTGAAATATACTAATCAACATGGTCATCAAATTTTGTGTCA
```

B-901 — L1MA9

```
megabat TGTGATTAGAAACTTGGTAACATTAGAC-ATTCCTATATCAATTGAAGTTTTCT---GGGAACC-TAACATTTCACTTACATGTATGTAGAACACTAAGATTTTTTCCCCCAATTTTATTGAGAAATAATTGACATACAATACTA--TTTAAAGTGTACGGCATGATGGTTTGATTTACACATACTGTGAAATGATTGCCACAATAGGTTCAGCTAACATTTACCATT
microbatTATTATTAGAAATTTTGTAACATTAGAC-ATTCCCATATCAATTAAAATTTTCTAGTGGGAGCC-TAACATTT-TACTACATGTGTGTGGAGCACAGGGATTTTTT---CCAATTTTATTAAGAAACAATTGACATATATCACTA--T----AAGTTTAAAGTGTACAGCATGATTTATACATGTTGTGAAATGGTTACCACAGATGTAGCTA-----ACATCCATCT
dog     CATTATTAGAAATTTTATGACGTTGGAC-TTTCCCATATCAGTCAAAATTTTCTACTGGGTGACTTAACATTT-GAATATATATGTATGTAGAATACTAGAAATTTTTTCCCATT---TTGAGAAATAATTGACATTACTGTATAACTTTAAGGCATACAGCATGATGATTGGATTTACATGTATTATGAAATGGTTACCACAGCAGGTTCAGATAATATCCATCATG
panda   CTTTATGAGAAATTTTGTGACATTGGAC-TTTCCCATATCAATCAAAATTTTCTACTGGGAAAC-TAACATTT-GGATATATATGTATGTAGAATACTAGAAATTTTTTCCTATTTTATTGAGAAATAATTGATATTACTGTATAACTTTAAGGCATACAGCATGATGGTTGGATTTACATGTATTGTGAAATGGTTGCCACAGTAGGTTCAGCTAACATCCATCATG
cow     TGTTATTGGAAATGTTGCTATATTGGACAATTCCCATATCAATTAAATTTTTCTACCAGAAGTG-TAACATTT-CAATAGATGTATGTAG------------------------------------------------------------------------------------------------------------------------------------------
alpaca  TGTTATTAGAAATTTTGTAACATTAGAT-ATTCCTGTATCAATTAAAATTTTCTACAGGGAGCC-TAACATTT-TAATATACATATAGAG------------------------------------------------------------------------------------------------------------------------------------------
dolphin TGTTATTAGCAATTTTGCAACATTAGAC-ATTCCCATATCAATTAAAGTTTTCTACTGGGAGCC-TAACATTT-CAATGTACGTATGTAG------------------------------------------------------------------------------------------------------------------------------------------
horse   TGTTAATAGAAATTTTGTGACATTTGAC-ATTCCCATATCAATTACAGTTTTCTACAGGGAGCC-TAGCATTTCCAGTACATGTGTGTGG------------------------------------------------------------------------------------------------------------------------------------------
hedgehogTATTATTAGAAATT-------ATTAGAC-TTTTCCACTTGAATTAAAAATATCTAGATGGGTC--TGATA----------GTGTGTGTAG------------------------------------------------------------------------------------------------------------------------------------------
human   TGCCATTAGAAATTTTGTAATATTAAAC-ATTCCTATACCAATTAAAAATTTTTACTGGGAGCC-TATTATTT-CAATATATGTGTGTAG------------------------------------------------------------------------------------------------------------------------------------------


megabat TTGTATAGATGCAATAGAAAGAAACG--------AAAAAAAAATTTTTTTCTCCTTTTGATGAGAACTCCTAGGATTTACTCAC------------------------------------------------------------------------------------------------------------------------------------------------
microbatTCTCAGAGATACATTGAAAAGAAAGA--------GAAAAAAAATTTTTTTCTCCTTGTTATGAGAACTCTGAGGGTTTACTCAC------------------------------------------------------------------------------------------------------------------------------------------------
dog     TCATATGGATACAGTAAACAGAAAAG-------------AAAAAAGCTTTCTCCTTGTGATGAGAACTCCTAGGATTTACTCTC----------------------AACTTTTTTTTTTTGAGAGAGAGGGTGTGGGGTTGAGGAACAGAGGGAAAGGGAGAAAGAGAATCCATCCTAAGCAAGCTCCACACCCAGCACGGAGCCCAGTGCGGGGCTTGATCTCACAA
panda   TCAAATGGATACAGTAAACAGAAAAGAGAGAAGAAAAAAAGAAAAGTTTTCTCCTTGTGATAAGAACTCTTAGGGTTTGTTCTCTTAACTTTCTTTTTCTTTTCTTTTCTTTTTTTTTTTAGAGAGGGAGGGGTGGGGTTGAGAAGCAGAGGGAGAGGGAGAGAGAGA----AATCTAAGCAGGCTCCACGCCCAGTGTGGAGCCCACTGTGGGGCTTGATTTCACAA
cow     ------------------------------------------------------------------------------------------------------------------------------------------------------------------------------------------------------------------------------------
alpaca  ------------------------------------------------------------------------------------------------------------------------------------------------------------------------------------------------------------------------------------
dolphin ------------------------------------------------------------------------------------------------------------------------------------------------------------------------------------------------------------------------------------
horse   ------------------------------------------------------------------------------------------------------------------------------------------------------------------------------------------------------------------------------------
hedgehog------------------------------------------------------------------------------------------------------------------------------------------------------------------------------------------------------------------------------------
human   ------------------------------------------------------------------------------------------------------------------------------------------------------------------------------------------------------------------------------------


megabat ----------------------------------------------------------------------TTAACTTTTCTGTATATCATATGGCAATGTTAACTGTAGTTATCATGCTGTACGTTATATCTTTAGTACTTTTTTATCTTACAACTGAAAGTTTGTACCTTTTGACCACCTTCCTCCAA-TTCTCCCACCCCTCAACCTCTACCACCTCTGGTAACAA
microbat----------------------------------------------------------------------TTAACTTTCTTATATATCATACAGCAGTGTTAACTATAGTTACCATATTGTACATTACACCCCTAGTACTTATTTATCTGATAACTAGAAG---------TTTGATCATCTAATGCCAA-TTTTCCATCCTCCCTACCTCTCCGCTGCCTCCTAACCA
dog     CCCTGAGATCATGACCTGTGCCTAGTC---AAGAGTCAGATGCTCAGCTGAGCCACCCAGGCGCCCCCTCTTAACTTTCTTATATATCAGAAAGCAGTGTTAGCTGTAGTCATCAGGTTGTACATTGCATCCCTAGTACTTATTTGTCTTATGATTGGACATTTTTACCTTTTGACTGCTTTCTTCTTATTCCCCCCTTTCTCCACCCTCTGTCACCTCTGGTAATCA
panda   TCCTGAGATCACAACCTGAGCCGAAATCAAGAGACACGCTCAACCAACTGAGCCACCCAGTCGCCCCCTCTTAACCCTCTTGTATACCACACAGCAGTGTTAGCTATAGTCATGAGGTTGTACATTGTATTCCTAGTACGTATTTATCTTATACCTGGAAGTTCGTACCTTTTGATTACCTTCCTTTTC-TCCCCCCTCTCTCCACCCTCTGCCGCCTCTGGTAACCA
cow     ------------------------------------------------------------------------------------------------------------------------------------------------------------------------------------------------------------------------------------
alpaca  ------------------------------------------------------------------------------------------------------------------------------------------------------------------------------------------------------------------------------------
dolphin ------------------------------------------------------------------------------------------------------------------------------------------------------------------------------------------------------------------------------------
horse   ------------------------------------------------------------------------------------------------------------------------------------------------------------------------------------------------------------------------------------
hedgehog------------------------------------------------------------------------------------------------------------------------------------------------------------------------------------------------------------------------------------
human   ------------------------------------------------------------------------------------------------------------------------------------------------------------------------------------------------------------------------------------


megabat CAAGTCTGATCTCTTTTTCTGAGGTTTGTTT---------------TGTTTTTGTTTTTTTAAGATTCTACATATAAGTGAGATCATCAAGTATTTGTTTTTCTTTGTCTGACTTATTCCACTTAGCATG----TCTTCAGGGTCATCCGCATTGTTGCAAATGGTTGAATTCCCTCATTTTTT-------------TATTTGGCTAAAACATATTCCATTGTATTTA
microbatCAAGTCTGACCTCTT---------TCTGAGT---------------CTGGGTTTTGTTTTTAAGATTCCACATATAAGTAAGATCATACAATATTTGTTTTTCTCTGTTTGACTTATTCCACTTAGCATAATGTCTTTAAGGCCCATCTGTGTTGTCACAGTGGTAGGATTTCCTATTTTTCTATCTGGCTGAATAGTACTG---------------CGTTTTATTTG
dog     CAAGTCTGATCTCTTTTTCTGGGTTTGGGTTTTTGGTTTTGA----TTATTTTGTTTTGTATTGTTCCCTGATATAAATGAGATCCTACAGTATTTGTTTTTCTGTATATGACTTATTTCACTTAACATAATGCCTTCAAGATCCATCTGTGTTATCTCAAATTGTAGGATTTCCTCATGTTTTTATGTCTGAGTAATATGCATATAATATTAATATACATGTACACA
panda   CAAGTCTGATCTCTTTTTCTGGGTTTGGGTTTTTGGTTTTTGGTTTTAATTTTTCTTTTTTTAAATTTCTGCTGTAAGTGAGATCCTACAGTATTTGTTTTTCTGTGTATGACTTATTTCACTTAGCATAATGCCTTCAAGCTCCATATGTGTTATCTCAAATGGTAGGATTTCCTCATTTTTTTATAGCTGAGTAATATGCATATAATATTAACATACATGCACACA
cow     ------------------------------------------------------------------------------------------------------------------------------------------------------------------------------------------------------------------------------------
alpaca  ------------------------------------------------------------------------------------------------------------------------------------------------------------------------------------------------------------------------------------
dolphin ------------------------------------------------------------------------------------------------------------------------------------------------------------------------------------------------------------------------------------
horse   ------------------------------------------------------------------------------------------------------------------------------------------------------------------------------------------------------------------------------------
hedgehog------------------------------------------------------------------------------------------------------------------------------------------------------------------------------------------------------------------------------------
human   ------------------------------------------------------------------------------------------------------------------------------------------------------------------------------------------------------------------------------------


megabat CATACCACAACATCAGTGGACAC--------------------AGGTTGTTTCCATGTCGTGGCTATTGTAAATAATGCAGCTATGAACATAAAGATGCAGATATCTTTTCAAGTTAGACAACACTAAGATTTTGATGAATAAGGCTATTCCAGGTAAAAGGAATACTGAAGCAAAAGAAGTACAAAGGTCTAGAGTTCTGGGACTTGTTAGAAGAC-AT-GAGGCAT
microbatTGGACCACAA---TTCTATCCATTCATCTATCCATGGACAGTGAGGTTGTTTCCATGTCT-----ATTGTAAATAATGCTGTTATGAACATGAGGGTGAG--------------------GACACTAGGA-TTTGATGGATGAGGTTATTCCAGGTAAAAGGAACACTGAAGCAAA-----------GGTGTAGAGTTACAGGACATGTTAGAGGAC-GA-GAGCCAT
dog     CATGCCACAACTTCTTTATCCGTTCATCCATCCATGGACACTTAGATTGTTTCTATGTCTTG---ACTATACATAATGCTGCTATGAACATGAGGGTACAGATTTCTTTTCAAGTT----AGCACTAGGATTT-----GATAAGGCTATTCCACATAAAACGCATACTATAGCAAAAG---TGCAAAAATCCAGAGTTCTAGTACATGATAGAGGACTGGTCAGCCAT
panda   CACCCCACAA---CTTTATCCATTCATCCATCAGTGGACACTTAGGTTGTTTCTGTGTCTTGGCTGCTGTACATAATGCTGCTATGAACATGGGGGTACAGATATCTTTTCAAGTTAGAGAACACTAGGATTTTGATTGATAAGGCAATTCTGGGTAAAAGGAATACTAAAACAAAAGAAATGCAAAAATCT--AGTTATAGGCCATGATAGAGGACTGATGAGCCAT
cow     ------------------------------------------------------------------------------------------------------------------------AACACTAGGATTTTTATGGATAAGACTGTTCCAGGTAAAAGGAATACTGATGCAAAAGAAGTGCAAAGGTCTCGAGTTGCAGGACATTGTAGAGATT-GGTAAGCTGT
alpaca  ------------------------------------------------------------------------------------------------------------------------AGCACTAGGATTTTGATAGATAAGACTATTCTAGGTAAAAGGAATACTGATGCAAAAAAAGTGCCAGGGTCTAGAGTTGGAGGACGTTTTAGACACC-ACTGAGCCAT
dolphin ------------------------------------------------------------------------------------------------------------------------AACACTAGGATTTTTATGGATAAGAC---TTCATGTAAAGGGAATACTGACGCAAAAGAAGTGCCAAGGTCTAGAGTTGCAGGACATTTTAGAGACT-GGTGAGCCGT
horse   ------------------------------------------------------------------------------------------------------------------------AACACTAGGACTTTGATGGATCAGGCTGCTCCA-GTAGAAGGAATACTGGAGGGAAAGAAGGAGAAAGGTCTAGAGCTGCAGGACATGTTAGAGGAT-GG-GAGCCAT
hedgehog------------------------------------------------------------------------------------------------------------------------AACACTAGGATTTTGATGAGTAAGGCTATTCTGGGCAGAATGAATACCGAAATAAAAGAAGTACAAAGGTCTGGGGTTGCAGG------TAGTGGAT-GGAAAGTC--
human   ------------------------------------------------------------------------------------------------------------------------ATCCCTAAGGTTTTGGCGGGTAAGTCAATTCCAGGTAAAA-GCACACTGAAGCAAAAGAATTACAAAGGTTTAGAGTTGCAGGATTTGTTAGAAGACTGGTGAGACAT


megabat CTTTCATCTCTATATTGCTCATGGTTCCTTTCACAGAG
microbatCTTTCATCTCTGTCTTCTTCATGGTGCCTTTCACAGAG
dog     CTTGCATCTC--TATTCCTCATGGTACCTTTCATAGAG
panda   CTTGCATCTCTTTATTCCTCATGGTACCTTTCAAAGAG
cow     CTTTCACC----TGTTTCTCATGTTGCCTTTCATAGTG
alpaca  CTTTCACCTC--TGTTCCTCAGGGTGCCTTTCACAGTG
dolphin CTTTCATCTC--TGTTCCTCATGGTGCCTTTCATAGCG
horse   CTTTCATCTC--TGCTCCTCATGGTGCCTTTCACAGAG
hedgehogTTTGCATCTCCATACTTGTCCTGAGGCCACTTGCAGAT
human   TTTTTATCTTTGTGCTCCTCATGGTGCCTTACACAGAG
```
