# Supplementary material for: A Genomic Approach to Examine the Complex Evolution of Laurasiatherian Mammals
Source: PLoS One. 2011 Dec 2;6(12):e28199. doi: 10.1371/journal.pone.0028199 (PMC3229520; doi:10.1371/journal.pone.0028199)
Supplement: Table S1 — Upper and lower bounds of the calibration points used in the divergence time estimation. (DOC) [file pone.0028199.s003.doc]

| **Divergence** | | **Minimum age** | **Maximum age** |
| --- | --- | --- | --- |
| Dolphin | Cow | 52,4 | 65,8 |
| Dog | Cat | 39,7 | 63,8 |
| Hedgehog | Shrew | 61,5 | 131,5 |
| Euarchontoglires | Laurasiatheria | 92 | 92 |
| Human | Mouse | 61,5 | 131,5 |
| Lipotyphla | CCCP | 61,5 | 131,5 |
